# Seasonality and geography of diabetes mellitus in United States of America dogs

**DOI:** 10.1371/journal.pone.0272297

**Published:** 2022-08-05

**Authors:** Liang N. Y. Qiu, Stephen V. Cai, Dennis Chan, Rebecka S. Hess

**Affiliations:** Department of Clinical Sciences & Advanced Medicine, School of Veterinary Medicine, University of Pennsylvania, Philadelphia, Pennsylvania, United States of America; University of Lincoln - Brayford Campus: University of Lincoln, UNITED KINGDOM

## Abstract

The diagnosis of type 1 diabetes mellitus (DM) in humans is associated with high altitude, few sunshine hours, cold climate, and winter. The goals of this study were to investigate seasonal and geographic patterns of DM diagnosis in United States of America (USA) dogs with juvenile and mature onset DM. Data were collected by means of an online survey widely distributed in the USA through breed clubs, academic veterinary institutions, private veterinary referral practices, social media outlets, and the American Kennel Club. Juvenile DM (JDM) and mature onset DM were defined as DM with an age of onset <365 days and DM with an age of onset ≥365 days, respectively. Meteorological seasons were defined as: winter from December through February, spring from March through May, summer from June through August, and fall from September through November. Four geographic regions were also defined as the West, North, South, and Central regions of the USA. Nonoverlapping 95% confidence intervals (CI) for season, geographic region, and breed specific proportions of dogs with JDM were considered statistically significantly different. The study included 933 dogs with mature onset DM and 27 dogs with JDM. Dogs were diagnosed with DM significantly more in the winter and northern USA compared to all other seasons and all other geographic regions, respectively. The prevalence of JDM among dogs with DM was 2.8%. The proportion of dogs with JDM among pure breeds was not significantly different than the proportion of JDM in mixed breed dogs. It is concluded that winter and cold climate could be shared environmental factors influencing DM expression in dogs and humans. Additionally, pure breed dogs do not appear to be at increased risk for JDM compared to mixed breed dogs, indicating that factors other than genetics could influence spontaneous JDM development in dogs.

## Introduction

Dogs develop a spontaneous form of diabetes mellitus (DM) that resembles type 1 DM in humans and is characterized by extreme loss of pancreatic beta cells and insulin deficiency [1, 2]. Most dogs with DM require exogenous insulin treatment, and diabetic ketoacidosis can develop in dogs with poorly controlled DM, as in humans with type 1 DM [3]. The diagnosis of type 1 DM in humans has been linked to high altitude, small number of sunshine hours per day, cold climate, and winter [4–6]. The reasons for these associations are incompletely understood, but low duration sunshine and low temperatures could influence vitamin D synthesis, insulin sensitivity, lifestyle, and diet. There are conflicting data regarding the association between vitamin D and DM in humans and some studies suggest that vitamin D concentration is unlikely to have a large effect on the risk of type 1 DM [7]. However, some of the literature, such as a study of 459 patients with type 1 DM and 208 age and sex matched non-diabetic controls, report significantly decreased plasma 25-hydroxyvitamin D concentration at the time of type 1 DM diagnosis compared to 25-hydroxyvitamin D concentration in matched controls [8]. A meta-analysis examining vitamin D receptor gene polymorphisms in patients with type 1 DM, from 19 geographic regions across the world, found that vitamin D receptor polymorphisms in regions with low ultraviolet radiation exposure differed from vitamin D receptor polymorphisms in locations with year-round ultraviolet radiation [9]. These findings of differences across geographical areas could provide an explanation for conflicting data from different countries regarding the association between vitamin D and type 1 DM in humans. A study of 1,117 non-diabetic men from Uppsala, Sweden investigated insulin sensitivity with the aid of euglycemic insulin clamps and oral glucose tolerance tests and found that insulin sensitivity decreased in the winter and with a drop of outdoor temperatures that was unrelated to seasonality [10].

Seasonality has also been examined in dogs with DM. A study of 250 Wisconsin dogs with DM found that DM diagnosis was significantly more common in January and February compared to other months [11]. Two other studies from the United States of America (USA) and the United Kingdom have pointed to a possible link between winter and the diagnosis of DM in dogs, however these findings lacked statistical significance testing [12, 13]. A study of 860 dogs from Sweden reported a statistically significant increase in incidence of DM diagnosis of females in the spring, but no seasonality was reported for male dogs [14]. Neuter status was not reported in this study, but most females were assumed to be intact [14]. An association between geography and the diagnosis of DM has not been reported in dogs and the seasonality of DM diagnosis across the entire USA has also not been reported in dogs. The goals of this study were therefore to investigate geographic and seasonal patterns of DM diagnosis in USA dogs with juvenile and mature onset DM. These patterns, reported here, could point to shared environmental influences in dogs with DM and humans with type 1 DM.

## Methods

Data were assembled from an online survey that was designed to investigate spontaneous DM in dogs across the USA (S1–S4 Appendixes). The survey was promoted by the American Kennel Club, numerous breed clubs, 29 academic veterinary institutions, 14 private veterinary referral practices, and various social media outlets, as previously reported [15, 16]. The request to complete the survey was mailed out every month between April 2017 until October 2019, to different venues. Data collected included date of birth, date at the time of DM diagnosis, insulin dose with which the dog was treated, breed, sex, neuter status, USA state of residence of the dog, and owner contact information. Neuter status and state of residence were recorded from the time the survey was completed. Entries were carefully reviewed, and duplicate or erroneous entries of dogs with diabetes insipidus were deleted as were entries for deceased dogs or dogs without DM. Erroneous age entries in which date of DM diagnosis preceded the date of birth were also deleted. Owners of dogs with an age of DM diagnosis greater than 16 years were contacted for verification and were excluded if the owners could not be contacted. Mixed breed dogs with a predominant pure breed were included with the predominant pure breed category. Juvenile DM (JDM) was defined as DM with an age of onset <365 days and mature onset DM was defined as DM with an age of onset ≥365 days. For the calculation of the proportion of dogs with JDM within each of the most represented breeds only breeds represented by more than 10 dogs with mature onset DM were analyzed.

Meteorological seasons were defined according to National Oceanic and Atmospheric Administration guidelines (https://www.ncei.noaa.gov/news/meteorological-versus-astronomical-seasons) as follows: winter from December through February, spring from March through May, summer from June through August, and fall from September through November. Four geographic regions were also defined based on National Oceanic and Atmospheric Administration guidance (https://www.ncdc.noaa.gov/monitoring-references/maps/us-climate-regions.php) and state of residence. The West included the Northwest (Washington State, Oregon, and Idaho) and West (California and Nevada). The North included the Northern Rockies and Plains (Montana, Wyoming, North Dakota, South Dakota, and Nebraska), the Upper Midwest (Minnesota, Iowa, Wisconsin, and Michigan), and the Northeast (Maine, New Hampshire, Vermont, New York, Pennsylvania, Massachusetts, Rhode Island, Connecticut, New Jersey, Delaware, and Maryland). The South included the Southwest (Utah, Arizona, Colorado, and New Mexico), South (Kansas, Oklahoma, Texas, Arkansas, Louisiana, and Mississippi), and Southeast (Virginia, North Carolina, South Carolina, Georgia, Alabama, and Florida). The Central region included the Ohio Valley (Missouri, Illinois, Indiana, Ohio, Tennessee, Kentucky, and West Virginia). Alaska and Hawaii were not included in the geographic regions.

The total number of dogs in each of these geographic regions was calculated based on data provided by the Pew Research Center which reports the number of households per state (https://www.pewresearch.org/fact-tank/2021/10/12/u-s-household-growth-over-last-decade-was-the-lowest-ever-recorded/) and a 2020 American Veterinary Medical Association survey which estimated that 45% of USA households had a dog with 1.46 dogs per such household [17].

Descriptive counts and frequencies are reported separately or collectively for dogs with JDM and mature onset DM. Continuous variables such as age and insulin dose were not normally distributed and are therefore described as median and range, and for age also with interquartile range. The Mann-Whitney test was used to compare the medians of these variables in dogs with JDM and mature onset DM. Sex and neuter status were analyzed as a four-category variable including neutered or intact females and males and as a two-category variable combining all males and all females. The proportions of each sex category with 95% CI were calculated for all dogs with DM and JDM. Nonoverlapping 95% CI for sex category, breed specific proportions of dogs with JDM among all dogs with DM, season, and geographic regions were considered statistically significantly different. A p value of <0.05 was considered significant for all tests. All statistical evaluations were performed using a statistical software package (Stata 14.0 for Mac, Stata Corporation, College Station, TX).

## Results

The initial number of study entries was 8,588. However, entries were excluded because they were duplicates (6,652 entries, 6,627 of these due to a single nonsensical entry appeared to be duplicated by a bot), did not have DM (898 entries), were entries of deceased dogs (23 entries), or had diabetes insipidus and not DM (13 entries). Thirty-six entries were deleted because the date of DM diagnosis preceded the date of birth. Six additional entries were deleted because the age of DM diagnosis, which spanned 16–113 years, was greater than 16 years and owners could not be contacted for verification. Five other entries in which the age of diagnosis was greater than 16 years were corrected and retained after contacting the owners. Ultimately, a total of 960 entries of dogs with DM were retained for analysis.

Juvenile DM was reported in 27 of 960 diabetic dogs with a JDM study prevalence of 2.8% among dogs with DM. Breed distribution of 27 dogs with JDM spanned 19 breed categories and included mixed breed dogs (7 dogs, 26%), German Shepherd (2 dogs, 7.4%), Labrador Retriever (2 dogs, 7.4%), and 1 each (3.7%) of the following 16 breeds: American Eskimo, Bichon Frise, Border Collie, Brittany Spaniel, Cardigan Welsh Corgi, Chow Chow, English Setter, Golden Retriever, Jack Russell Terrier, Kerry Blue Terrier, Maltese, Miniature Schnauzer, Old English Sheepdog, Tibetan Spaniel, Weimaraner, and West Highland White Terrier. Seven of the 19 breed categories (32%) with JDM were also represented by more than 10 dogs with mature onset DM. The proportion of dogs with JDM within each of these breeds was not significantly different than the proportion of JDM in mixed breed dogs as determined by nonoverlapping 95% CI (Table 1). Owners and breeders of pure breed dogs with JDM were contacted to inquire about JDM in offspring or other relatives of the dog, and none were aware of related dogs with JDM or mature onset DM. Breeds of dogs with mature onset DM represented in the study by >10 dogs are listed in Table 2.

**Table 1:** The proportion of dogs with juvenile diabetes mellitus in breeds represented by >10 dogs with mature onset diabetes mellitus.

Breed	Number of dogs with mature onset diabetes mellitus	Number of dogs with juvenile diabetes mellitus	Breed specific proportion of dogs with juvenile diabetes mellitus (%)	Standard error	95% confidence interval
**Mixed breed dogs**	242	7	2.9	1.1	1.4–6.0
**Labrador Retriever**	64	2	3.1	2.2	0.7–12.1
**Miniature Schnauzer**	46	1	2.2	2.2	0.3–14.8
**West Highland White Terrier**	34	1	2.9	2.9	0.4–19.2
**American Eskimo**	25	1	4.0	4.0	0.5–26.4
**Bichon Frise**	21	1	4.8	4.8	0.6–30.9
**Maltese**	11	1	9.1	9.1	0.8–53.7

**Table 2:** Breeds of dogs with mature onset diabetes mellitus represented in the study by >10 dogs.

Breed	Number of dogs	Percent of dogs with mature onset diabetes mellitus (total of 933 dogs)
**Mixed breed**	235	25.2
**Labrador Retriever**	62	6.6
**Miniature Schnauzer**	45	4.8
**Pug**	44	4.7
**Miniature Pinscher**	38	4.1
**West Highland White Terrier**	33	3.5
**Australian Terrier**	31	3.3
**Pembroke Welsh Corgi**	25	2.7
**American Eskimo**	24	2.6
**Dachshund**	23	2.5
**Bichon Frise**	20	2.1
**Siberian Husky**	17	1.8
**Yorkshire Terrier**	15	1.6
**Chihuahua**	15	1.6
**Toy Poodle**	13	1.4
**Cairn Terrier**	11	1.2
**Havanese**	11	1.2
**Pomeranian**	11	1.2
**Australian Shepherd**	11	1.2

Among dogs with JDM, there were no significant differences in sex and neuter status, when sex and neuter status was analyzed as a four or two-category variable (Table 3). However, among dogs with mature onset DM, there were significantly more neutered females and neutered males compared to intact females and intact males (Table 3). When analyzing sex and neuter status as a two-category variable, there was no significant difference in the proportion of all females compared to the proportion of all males in dogs with mature onset DM (Table 3).

**Table 3:** The proportion of different sex and neuter status categories in dogs with juvenile diabetes mellitus and mature onset diabetes mellitus.

Type of diabetes by age of onset	Sex and neuter status	Number of dogs with diabetes mellitus	Proportion of dogs with diabetes mellitus (%)	Standard error	95% confidence interval
**Juvenile diabetes mellitus (27 dogs)**	Neutered females	9	33.3	9.2	17.5–54.0
	Neutered males	9	33.3	9.2	17.5–54.0
	Intact females	2	7.4	5.1	1.7–27.2
	Intact males	7	26	8.6	12.2–46.7
	All females	11	40.7	9.6	23.2–61.0
	All males	16	59.3	9.6	39.0–76.8
**Mature onset diabetes mellitus (916 dogs)**	Neutered females	417	45.5	1.6	42.3–48.8
	Neutered males	451	49.2	1.6	46.0–52.5
	Intact females	22	2.40	0.5	1.6–3.6
	Intact males	26	2.8	0.5	1.9–4.1
	All females	439	48	1.6	44.7–51.2
	All males	477	52	1.6	48.8–55.3

Median age of 27 dogs with JDM was 88 days (range 3–267, interquartile range 124 days) and the median age of dogs with mature onset DM reported in 861 dogs was 3,189 days (8.7 years) with a range of 365–5842 days (1–16 years), and interquartile range of 1,356 days (3.7 years). Median insulin doses reported in 26 dogs with JDM (0.5 units/kg, range 0.04–1.4 units/kg) were not significantly different than median insulin doses reported in 849 dogs with mature onset DM (0.6 units/kg, range 0.05–4.1 units/kg).

Dogs were diagnosed with DM significantly more in the winter compared to all other seasons as determined by nonoverlapping 95% CI (Table 4). Seasonality was available for analysis in 650 dogs with mature onset DM and 19 dogs with JDM. There was no significant difference in the seasonality of mature onset DM diagnosis and JDM diagnosis, nor was there a significant difference in seasonality of JDM diagnosis among 19 dogs with JDM.

**Table 4:** Seasonality of diabetes mellitus diagnosis in 669 dogs with diabetes mellitus.

Season	Number of dogs with diabetes mellitus	Proportion of dogs with diabetes mellitus (%)	Standard error	95% confidence interval
**Winter**	218	32.6	1.8	29.1–36.2
**Spring**	162	24.2	1.7	21.1–27.6
**Summer**	159	23.8	1.6	20.7–27.1
**Fall**	130	19.4	1.5	16.6–22.6

Dogs were diagnosed with DM significantly more in the northern USA compared to all other geographic regions as determined by nonoverlapping 95% CI (Table 5). Geographic region was available for analysis in 844 dogs with mature onset DM and 24 dogs with JDM. There was no significant difference in the geographic region of mature onset DM diagnosis and JDM diagnosis, nor was there a significant difference in geographic region of JDM diagnosis among 24 dogs with JDM. Based on data provided by the Pew Research Center and the American Veterinary Medical Association it is estimated that the number of dogs in the South, North, Central, and West regions are 31,740,197, 24,453,921, 13,296,486, and 13,124,333, respectively.

**Table 5:** Geographic region of 868 dogs with diabetes mellitus.

Geographic region	Number of dogs with diabetes mellitus	Proportion of dogs with diabetes mellitus (%)	Standard error	95% confidence interval
**West**	103	11.9	1.1	9.9–14.2
**North**	395	45.5	1.7	42.2–48.8
**South**	237	27.3	1.5	24.4–30.4
**Central**	133	15.3	1.2	13.1–17.9

## Discussion

Results of this study suggest that in USA dogs, DM is diagnosed more commonly during the winter season and in the northern part of the USA. These findings are well documented in humans with type 1 DM and are reported here for the first time in USA dogs [4–6]. The winter season and the northern USA are associated with colder temperatures and fewer hours of sunshine compared to other seasons and geographic regions, respectively. Cold temperatures and few hours of sunshine are associated with decreased insulin sensitivity and mutations in the vitamin D receptor in humans and could account for the increased incidence of type 1 DM diagnosis observed in humans [8–10]. Future studies of the effect of temperature on insulin sensitivity in dogs could help determine if cold influences the pathophysiology of canine DM. Although there is some evidence that extremely cold temperatures can induce hyperglycemia and possible insulin resistance in experimental dog models, the clinical significance of these findings in dogs with spontaneous disease is not known [18, 19]. Future studies of polymorphisms of the vitamin D receptor located on *Canis familiaris* chromosome 27:6,852,915–6,909,466 could also help elucidate the role of the vitamin D receptor in the etiology of canine DM. While hypocalcemia has been reported in some dogs with naturally occurring DM, it is not known if this documented hypocalcemia is related to alterations in vitamin D [3, 20]. Furthermore, unlike humans, dogs are unable to adequately synthesize vitamin D in the skin and rely on dietary intake as the main source of vitamin D [21]. Therefore, the effect of sunshine on vitamin D synthesis is likely not an important contributor to disease pathophysiology in dogs. Viral infections have been another suggested explanation for the association between cold temperatures, winter, and type 1 DM in humans. A recent meta-analysis of 38 case control studies including approximately 3000 people with type 1 DM and a similar number of controls found that enterovirus infection was associated with type 1 diabetes globally and in each of 5 different continents [22]. The mechanisms by which enteroviruses are associated with type 1 diabetes are unknown. However, coxsackie and adenovirus, both enteroviruses, can infect insulin producing beta pancreatic cells by binding the coxsackie and adenovirus receptor and replicating in the cells. These enterovirus infections can then induce beta pancreatic cell death and decrease insulin synthesis and secretion [22–24]. Another study examining antibodies against enteroviruses and respiratory viruses in children with and without type 1 DM found a significant association between the presence of such antibodies and type 1 DM and noted that most of the children with type 1 DM were diagnosed during the fall or winter seasons when outdoor temperatures were relatively cold [25]. This study also found that 90% of viral infections occurred in the moderate to cold temperature months between November-April [25]. Future studies of viral infections as a possible contributing etiology in the pathogenesis of DM in dogs could be of particular interest at a time of heightened attention to zoonotic diseases [26]. Although the association between type 1 DM, winter, and cold climate regions is not fully understood in humans, and has yet to be studied in dogs, the shared DM risk associated with winter and cold climate in humans and dogs suggests that similar environmental factors could be influencing disease expression in both species.

The findings reported here are different than the findings reported in 860 dogs with DM from Sweden in which females, but not males, were more likely to develop DM in the spring compared to other seasons [14]. It is possible that the reason for the difference between the two studies is that most female dogs in Sweden are intact, whereas most of the dogs and females in this study were neutered. Intact female dogs can develop a form of DM which is different than type 1 DM because higher growth hormone concentrations contribute to insulin resistance [27].

Juvenile DM is a rare condition in dogs and has been reported anecdotally in a small number of dogs [1, 28–42]. To the authors knowledge, this is the largest collection of dogs with JDM reported in the literature. Several epidemiologic studies excluded young dogs, and therefore the 2.8% prevalence of JDM among USA dogs with DM, is reported here for the first time in over three decades [14, 41, 43, 44]. In 1988, the prevalence of JDM among dogs with DM was estimated to be 1.2% based on a review of 16 studies which included 2,141 dogs with DM [41]. Results of our study suggest that the proportion of dogs with JDM in pure breed dogs is not different than the proportion of JDM in mixed breed dogs. Historically, JDM has also been reported in many different breeds [1, 28–42]. In this study, none of the owners or breeders of pure breed dogs with JDM were aware of other related dogs with JDM or mature inset DM. This is consistent with previous reports which studied healthy littermates of dogs with spontaneous JDM [42]. This pattern of disease expression in multiple different breeds is different than the pattern observed in mature onset DM, in which certain breeds are at increased risk for the disease [14, 43–46]. These findings suggest that factors other than genetic determinants could be influencing the expression of spontaneous DM in young dogs, although genetic JDM has been reported in purpose bred Keeshonds and Golden Retrievers [36, 37, 47, 48]. Although JDM remains rare in dogs, there appears to be an increase in the prevalence of canine JDM over the past 30 years [41]. It is possible that shared human and canine environmental factors have influenced the increase in canine JDM and human type 1 DM [49].

The sex distribution of dogs with mature onset DM in this study was different from what has been reported in other studies, which have also differed from one another. In our study, there were significantly more neutered dogs compared to intact dogs, and there was no significant difference in the proportion of all females compared to the proportion of all males. In a Swedish study, dogs were almost exclusively female in some breeds, and neuter status was unknown [14]. In an Australian study, neutered males had a higher risk for mature onset DM compared to intact males and neutered females, and in a United Kingdom study, intact females and neutered males had a higher risk than intact males [43, 44]. These study differences could reflect population differences or be linked to varying disease risks in separate countries. The differences could also be due to different study designs, as the current study had no non-diabetic comparison group.

This study has several limitations. One limitation is that it included a small number of dogs with JDM. Therefore, some of the 95% CI for the proportion of dogs with JDM in breeds represented by >10 dogs in the study are wide, indicating that the estimations might not be precise. Furthermore, the lack of differences in seasonality and geographic region among dogs with JDM might be because of the small number of JDM dogs available for analysis. Due to the nature of data collection, which relied on dog owners completing a survey, owner recall bias could contribute to incorrect data. To decrease the risk of recall bias, entries of deceased dogs were excluded. Future studies may wish to exclude deceased dogs only if they died years ago. It is possible that centralized veterinary companion animal surveillance systems such as those employed in the United Kingdom and Australia or data from large insurance companies used to study canine DM in Sweden would provide more abundant and accurate data, but such data sources are currently unavailable in the USA [14, 43, 44]. Despite these limitations, a comparable number of 960 dogs with DM was reported in this study, compared to 418–1,272 dogs with DM reported from countries with centralized data sources [14, 43, 44].

In addition to recall bias, the use of a survey for data collection has other limitations. The survey was widely distributed in many venues throughout the USA, and as many owners as possible were encouraged to complete it. However, sample collection might not have been random and therefore might not accurately reflect the USA population of diabetic dogs. For example, it is possible that people in the northern USA are more likely to respond to surveys, leading to an erroneous conclusion that dogs are diagnosed with DM significantly more in the northern USA compared to all other geographic regions. The absolute number of dogs in each of the four geographic regions did not appear to influence the results in that the South has the highest estimated number of total dogs, but the largest number of diabetic dogs was reported in the North. Some of the venues that promoted the survey (such as breed club web pages, social media outlets, and the American Kennel Club) made the survey equally available to everyone on their web sites and through routine outreach efforts, regardless of geographic location. However, private practices (throughout the USA) may have promoted the survey locally. Some universities distributed the survey to their student body (from across the country), and anyone receiving the survey was asked to pass it on. Therefore, it is not known whether the survey was distributed more widely in one area of the USA versus another, whether urban or rural dogs were overrepresented, whether the population of dogs reported here truly represents the population of USA dogs with DM, and what factors influenced some people to complete the survey and others not to do so. Neuter status and state of residence were recorded from the time the survey was completed, but it is possible that neuter status and state of residence were different at the time of DM diagnosis. Recording the neuter status and state of residence from the time the survey was completed decreased the risk of recall bias, but could have led to erroneous data collection in regard to risk factors influencing the onset of DM. Further follow up studies in which different methodologies are used to corroborate or refute these findings are needed.

In conclusion, in USA dogs, DM is diagnosed more commonly during the winter season and in the northern part of the USA. The incidence of type 1 diabetes in humans is also associated with high altitude, small number of sunshine hours per day, cold climate, and winter [4–6]. Future research can be focused on whether similar environmental factors influence the onset of diabetes in dogs and humans. The prevalence of JDM among dogs with DM has increased to 2.8% and remains rare. Spontaneous JDM develops in many breeds and no breed appears to be at increased risk for JDM compared to mixed breed dogs. Therefore, factors other than genetics are suspected to influence naturally occurring JDM in dogs.

## Supporting information

S1 Appendix Diabetes mellitus survey. (PDF)

S2 Appendix Academic and private veterinary hospitals which promoted the survey. (PDF)

S3 Appendix Survey and data legend. (PDF)

S4 Appendix Data. (PDF)
