# Supplementary material for: Seasonality and geography of diabetes mellitus in United States of America dogs
Source: PLoS One. 2022 Aug 5;17(8):e0272297. doi: 10.1371/journal.pone.0272297 (PMC9355170; doi:10.1371/journal.pone.0272297)
Supplement: S1 Appendix — (PDF) [file pone.0272297.s001.pdf]

Diabetes Mellitus Survey

Dear Dedicated Pet Owner,

THANK YOU (!) for participating in this survey about your pet. The survey will advance our understanding of diabetes in dogs, and will allow us to determine how common the disease is in the general population as well as in specific dog breeds. This survey will enable Drs. Cai and Hess, at the Veterinary School of the University of Pennsylvania, to focus future studies on the appropriate breeds. The survey could also provide insight into the mode of inheritance (and possible future prevention) of diabetes in specific breeds.

The information submitted in this survey is confidential. Your personal information will be used only for the University of Pennsylvania researchers to contact you, and will not be made public. Findings from this survey could be included in scientific publications. However, identifying information pertaining to you and your dog will not be published. The results of this survey will likely not benefit your dog, but results of this survey will allow us to conduct future genetic studies which could benefit pets, pet owners, and breeders, and could facilitate disease prevention.

The survey takes about 10 minutes to complete (longer if you have more than one diabetic dog in your household). You can save your answers and return to the survey at any point before final submission to our database. Your time and input are greatly appreciated.

Please do not hesitate to contact Dr. Cai (cais@upenn.edu) if you have any questions regarding this survey. All of the participants will be entered into one of several raffles for $100 Amazon gift cards! Thank you very much for your participation.

Please complete the survey below.

Thank you!

Your (Owner) Contact Information

First Name ______________________________

Last Name ______________________________

Phone Number ______________________________

Email Address ______________________________

State of Address:

◯ AK
◯ AL
◯ AR
◯ AZ
◯ CA
◯ CO
◯ CT
◯ DE
◯ FL
◯ GA
◯ HI
◯ IA
◯ ID
◯ IL
◯ IN
◯ KS
◯ KY
◯ LA
◯ MA
◯ ME
◯ MD
◯ MI
◯ MN
◯ MO
◯ MS
◯ MT
◯ NC
◯ ND
◯ NE
◯ NH
◯ NJ
◯ NM
◯ NV
◯ NY
◯ OH
◯ OK
◯ OK
◯ OR
◯ PA
◯ RI
◯ SC
◯ SD
◯ TN
◯ TX
◯ UT
◯ VT
◯ VA
◯ WA
◯ WI
◯ WV
◯ WY

Today's Date

____

Your Pet's Information

What is your pet's call name?

____

Does your pet have an AKC-registered name?

◯ Yes
◯ No

What is your pet's AKC-registered name?

____

What is your pet's breed?

◯ Samoyed
◯ Samoyed mix
◯ Australian Terrier
◯ Australian Terrier mix
◯ Pug
◯ Pug mix
◯ American Eskimo
◯ American Eskimo mix
◯ Mixed breed
◯ Other pure breed
◯ German Shepherd
◯ German Shepherd mix
◯ Golden Retriever
◯ Golden Retriever mix
◯ American Pit Bull Terrier
◯ American Pit Bull Terrier mix
◯ Labrador Retriever
◯ Labrador Retriever mix

What is your pet's breed?

____

What is your pet's date of birth (approximate if unknown)?

____

What is your pet's age today?

◯ Less than 6 months old
◯ 6 months to 1 year old
◯ 1 year 1 month
◯ 1 year 2 months
◯ 1 year 3 months
◯ 1 year 4 months
◯ 1 year 5 months
◯ 1 year 6 months
◯ 1 year 7 months
◯ 1 year 8 months
◯ 1 year 9 months
◯ 1 year 10 months
◯ 1 year 11 months
◯ 2 years
◯ 2 years 1 month
◯ 2 years 2 months
◯ 2 years 3 months
◯ 2 years 4 months
◯ 2 years 5 months
◯ 2 years 6 months
◯ 2 years 7 months
◯ 2 years 8 months
◯ 2 years 9 months
◯ 2 years 10 months
◯ 2 years 11 months
◯ 3 years
◯ 3 years 1 month
◯ 3 years 2 months
◯ 3 years 3 months
◯ 3 years 4 months
◯ 3 years 5 months
◯ 3 years 6 months
◯ 3 years 7 months
◯ 3 years 8 months
◯ 3 years 9 months
◯ 3 years 10 months
◯ 3 years 11 months
◯ 4 years
◯ 4 years 1 month
◯ 4 years 2 months
◯ 4 years 3 months
◯ 4 years 4 months
◯ 4 years 5 months
◯ 4 years 6 months
◯ 4 years 7 months
◯ 4 years 8 months
◯ 4 years 9 months
◯ 4 years 10 months
◯ 4 years 11 months
◯ 5 years
◯ 5 years 1 month
◯ 5 years 2 months
◯ 5 years 3 months
◯ 5 years 4 months
◯ 5 years 5 months
◯ 5 years 6 months
◯ 5 years 7 months
◯ 5 years 8 months
◯ 5 years 9 months
◯ 5 years 10 months
◯ 5 years 11 months
◯ 6 years
◯ 6 years 1 month
◯ 6 years 2 months
◯ 6 years 3 months
◯ 6 years 4 months
◯ 6 years 5 months
◯ 6 years 6 months
◯ 6 years 7 months

◯ 6 years 8 months
◯ 6 years 9 months
◯ 6 years 10 months
◯ 6 years 11 months
◯ 7 years
◯ 7 years 1 month
◯ 7 years 2 months
◯ 7 years 3 months
◯ 7 years 4 months
◯ 7 years 5 months
◯ 7 years 6 months
◯ 7 years 7 months
◯ 7 years 8 months
◯ 7 years 9 months
◯ 7 years 10 months
◯ 7 years 11 months
◯ 8 years
◯ 8 years 1 month
◯ 8 years 2 months
◯ 8 years 3 months
◯ 8 years 4 months
◯ 8 years 5 months
◯ 8 years 6 months
◯ 8 years 7 months
◯ 8 years 8 months
◯ 8 years 9 months
◯ 8 years 10 months
◯ 8 years 11 months
◯ 9 years
◯ 9 years 1 month
◯ 9 years 2 months
◯ 9 years 3 months
◯ 9 years 4 months
◯ 9 years 5 months
◯ 9 years 6 months
◯ 9 years 7 months
◯ 9 years 8 months
◯ 9 years 9 months
◯ 9 years 10 months
◯ 9 years 11 months
◯ 10 years
◯ 10 years 1 month
◯ 10 years 2 months
◯ 10 years 3 months
◯ 10 years 4 months
◯ 10 years 5 months
◯ 10 years 6 months
◯ 10 years 7 months
◯ 10 years 8 months
◯ 10 years 9 months
◯ 10 years 10 months
◯ 10 years 11 months
◯ 11 years
◯ 11 years 1 month
◯ 11 years 2 months
◯ 11 years 3 months
◯ 11 years 4 months
◯ 11 years 5 months
◯ 11 years 6 months
◯ 11 years 7 months
◯ 11 years 8 months
◯ 11 years 9 months
◯ 11 years 10 months
◯ 11 years 11 months
◯ 12 years
◯ 12 years 1 month
◯ 12 years 2 months
◯ 12 years 3 months
◯ 12 years 4 months
◯ 12 years 5 months
◯ 12 years 6 months

◯ 12 years 7 months
◯ 12 years 8 months
◯ 12 years 9 months
◯ 12 years 10 months
◯ 12 years 11 months
◯ 13 years
◯ 13 years 1 month
◯ 13 years 2 months
◯ 13 years 3 months
◯ 13 years 4 months
◯ 13 years 5 months
◯ 13 years 6 months
◯ 13 years 7 months
◯ 13 years 8 months
◯ 13 years 9 months
◯ 13 years 10 months
◯ 13 years 11 months
◯ 14 years
◯ 14 years 1 month
◯ 14 years 2 months
◯ 14 years 3 months
◯ 14 years 4 months
◯ 14 years 5 months
◯ 14 years 6 months
◯ 14 years 7 months
◯ 14 years 8 months
◯ 14 years 9 months
◯ 14 years 10 months
◯ 14 years 11 months
◯ 15 years
◯ 15 years 1 month
◯ 15 years 2 months
◯ 15 years 3 months
◯ 15 years 4 months
◯ 15 years 5 months
◯ 15 years 6 months
◯ 15 years 7 months
◯ 15 years 8 months
◯ 15 years 9 months
◯ 15 years 10 months
◯ 15 years 11 months
◯ 16 years
◯ 16 years 1 month
◯ 16 years 2 month
◯ 16 years 3 months
◯ 16 years 4 months
◯ 16 years 5 months
◯ 16 years 6 months
◯ 16 years 7 months
◯ 16 years 8 months
◯ 16 years 9 months
◯ 16 years 10 months
◯ 16 years 11 months
◯ 17 years
◯ 17 years 1 month
◯ 17 years 2 months
◯ 17 years 3 months
◯ 17 years 4 months
◯ 17 years 5 months
◯ 17 years 6 months
◯ 17 years 7 months
◯ 17 years 8 months
◯ 17 years 9 months
◯ 17 years 10 months
◯ 17 years 11 months
◯ 18 years
◯ 18 years 1 month
◯ 18 years 2 months
◯ 18 years 3 months
◯ 18 years 4 months
◯ 18 years 5 months

◯ 18 years 6 months
◯ 18 years 7 months
◯ 18 years 8 months
◯ 18 years 9 months
◯ 18 years 10 months
◯ 18 years 11 months
◯ 19 years
◯ Over 19 years old

What is your pet's sex?

◯ Intact male
◯ Intact female
◯ Neutered male
◯ Spayed female

What is your dog's approximate weight in pounds?

____

What diet (brand and/or type) are you feeding your dog?

____

Your Pet's Health Status

Is your pet diabetic?

◯ Yes
◯ No

Is your dog receiving insulin?

◯ Yes
◯ No

Which type of insulin is your dog receiving?

◯ NPH / Humulin-N / Novolin-N
◯ Lantus / Glargine
◯ Vetsulin
◯ PZI
◯ Levemir / Detemir
◯ Other

What is the name of insulin which your dog is receiving?

____

How many times a day does your dog receive insulin?

◯ Once daily
◯ Twice daily
◯ Three times daily

How many units of insulin does your dog receive with each injection?	◯ 1 units<br>◯ 2 units<br>◯ 3 units<br>◯ 4 units<br>◯ 5 units<br>◯ 6 units<br>◯ 7 units<br>◯ 8 units<br>◯ 9 units<br>◯ 10 units<br>◯ 11 units<br>◯ 12 units<br>◯ 13 units<br>◯ 14 units<br>◯ 15 units<br>◯ 16 units<br>◯ 17 units<br>◯ 18 units<br>◯ 19 units<br>◯ 20 units<br>◯ 21 units<br>◯ 22 units<br>◯ 23 units<br>◯ 24 units<br>◯ 25 units<br>◯ 26 units<br>◯ 27 units<br>◯ 28 units<br>◯ 29 units<br>◯ 30 units<br>◯ 31 units<br>◯ 32 units<br>◯ 33 units<br>◯ 34 units<br>◯ 35 units or more
When was your dog diagnosed with diabetes (approximate if unknown)?	________________
What is your veterinarian's name (if known)?	________________
What is your veterinarian's phone number (if known)?	________________
What were the clinical signs that you noticed when your dog was diagnosed with diabetes (if recallable)?	☐ Increased thirst or urination<br>☐ Increased hunger<br>☐ Weight loss
Has your dog been diagnosed with hypothyroidism?	◯ Yes<br>◯ No
Is your dog being treated with thyroid supplementation?	◯ Yes<br>◯ No
Has your dog been diagnosed with Addison's disease (hypoadrenocorticism)?	◯ Yes<br>◯ No

Is your dog currently being treated with any of the following medications?	☐ Prednisone<br>☐ DOCP<br>☐ Prednisone and DOCP<br>☐ Fludricortisone (e.g. Florinef)
Was the prednisone given before or after the diagnosis of diabetes?	☐ Before diagnosis of diabetes<br>☐ After diagnosis of diabetes
How much prednisone (in mg) is given per dose (each time you give the medication)?	______________________
How many times per day do you give this prednisone dose?	◯ Once daily<br>◯ Twice daily<br>◯ Other
Does your dog have any other concurrent illnesses?	◯ Yes<br>◯ No
Please list the concurrent conditions/illnesses.	______________________
Did you obtain your pet from a breeder?	◯ Yes<br>◯ No
What is your pet's breeder's name (if known)?	______________________
What is your pet's breeder's phone number (if known)?	______________________
What is your pet's breeder's email?	______________________
Was your dog obtained from a rescue group?	◯ Yes<br>◯ No
What is the name of your dog's rescue?	______________________

Your Pet's Relatives

Are you in contact with the owner of your dog's mother?	◯ Yes<br>◯ No
Who is the owner of your dog's mother (if known)?	______________________
What is this owner's email address (if known)?	______________________
What is the name of your dog's mother (if known)?	______________________

Is your dog's mother still alive (if known)?
◯ Yes
◯ No
◯ Unsure

Are you in contact with the owner of your dog's father?
◯ Yes
◯ No

Who is the owner of your dog's father (if known)?
____

What is this owner's email address (if known)?
____

What is the name of your dog's father (if known)?
____

Is your dog's father still alive?
◯ Yes
◯ No
◯ Unsure

Does your dog have any littermates?
◯ Yes
◯ No
◯ Unknown

Are you able to give any information regarding your pet's littermates?
◯ Yes
◯ No

How many littermates did your dog have?
____

What is the name(s) of your dog's littermates (list all that are known)?
____

What is the email address of the owner(s) of your dog's littermates (list all known)?
____

Does your dog have any full siblings (shares same father and mother) from a DIFFERENT litter(s)?
◯ Yes
◯ No
◯ Unknown

Are you able to give any information pertaining to these full siblings?
◯ Yes
◯ No

Approximately how many other full siblings (from other litters) does your dog have?
◯ 1-5
◯ 6-10
◯ 11-15
◯ 16-20
◯ Greater than 20

What is the name(s) of your dog's full siblings (list any that are known)
____

What is the email address of the owner(s) of your dog's full siblings (list if known):
____

Does your dog have any offspring?	◯ Yes<br>◯ No<br>◯ Unknown
How many litters did your pet produce?	__________________________________
Are you able to provide any information pertaining to your pet's offspring?	◯ Yes<br>◯ No
What is the name(s) of your dog's offspring?	__________________________________
What is the email address of the owner(s) of your dog's offspring?	__________________________________

Your Pet's Housemates

Are there any other dogs living in the household?	◯ Yes<br>◯ No
How many other dogs live in the household?	◯ 1<br>◯ 2<br>◯ 3<br>◯ 4<br>◯ 5<br>◯ 6<br>◯ 7<br>◯ 8<br>◯ 9<br>◯ 10<br>◯ 11<br>◯ 12<br>◯ 13<br>◯ 14<br>◯ 15<br>◯ Greater than 15
Are any of these household dogs diabetic?	◯ Yes<br>◯ No
How many household dogs are diabetic?	◯ 1<br>◯ 2<br>◯ 3<br>◯ 4<br>◯ 5<br>◯ Greater than 5
What is the diabetic household dog's call name?	__________________________________
Does THIS diabetic household dog have an AKC-registered name?	◯ Yes<br>◯ No
What is THIS diabetic household dog's AKC-registered name?	__________________________________

What is THIS diabetic household dog's breed?

- ◯ Samoyed
- ◯ Samoyed mix
- ◯ Australian Terrier
- ◯ Australian Terrier mix
- ◯ Pug
- ◯ Pug mix
- ◯ American Eskimo
- ◯ American Eskimo mix
- ◯ Mixed breed
- ◯ Other pure breed
- ◯ German Shepherd
- ◯ German Shepherd mix
- ◯ Golden Retriever
- ◯ Golden Retriever mix
- ◯ American Pit Bull Terrier
- ◯ American Pit Bull Terrier mix
- ◯ Labrador Retriever
- ◯ Labrador Retriever mix

What is THIS diabetic household dog's breed?

____

What is THIS diabetic household dog's date of birth?

____

What is THIS diabetic household dog's age today?

◯ Less than 6 months old
◯ 6 months to 1 year old
◯ 1 year 1 month
◯ 1 year 2 months
◯ 1 year 3 months
◯ 1 year 4 months
◯ 1 year 5 months
◯ 1 year 6 months
◯ 1 year 7 months
◯ 1 year 8 months
◯ 1 year 9 months
◯ 1 year 10 months
◯ 1 year 11 months
◯ 2 years
◯ 2 years 1 month
◯ 2 years 2 months
◯ 2 years 3 months
◯ 2 years 4 months
◯ 2 years 5 months
◯ 2 years 6 months
◯ 2 years 7 months
◯ 2 years 8 months
◯ 2 years 9 months
◯ 2 years 10 months
◯ 2 years 11 months
◯ 3 years
◯ 3 years 1 month
◯ 3 years 2 months
◯ 3 years 3 months
◯ 3 years 4 months
◯ 3 years 5 months
◯ 3 years 6 months
◯ 3 years 7 months
◯ 3 years 8 months
◯ 3 years 9 months
◯ 3 years 10 months
◯ 3 years 11 months
◯ 4 years
◯ 4 years 1 month
◯ 4 years 2 months
◯ 4 years 3 months
◯ 4 years 4 months
◯ 4 years 5 months
◯ 4 years 6 months
◯ 4 years 7 months
◯ 4 years 8 months
◯ 4 years 9 months
◯ 4 years 10 months
◯ 4 years 11 months
◯ 5 years
◯ 5 years 1 month
◯ 5 years 2 months
◯ 5 years 3 months
◯ 5 years 4 months
◯ 5 years 5 months
◯ 5 years 6 months
◯ 5 years 7 months
◯ 5 years 8 months
◯ 5 years 9 months
◯ 5 years 10 months
◯ 5 years 11 months
◯ 6 years
◯ 6 years 1 month
◯ 6 years 2 months
◯ 6 years 3 months
◯ 6 years 4 months
◯ 6 years 5 months
◯ 6 years 6 months
◯ 6 years 7 months

◯ 6 years 8 months
◯ 6 years 9 months
◯ 6 years 10 months
◯ 6 years 11 months
◯ 7 years
◯ 7 years 1 month
◯ 7 years 2 months
◯ 7 years 3 months
◯ 7 years 4 months
◯ 7 years 5 months
◯ 7 years 6 months
◯ 7 years 7 months
◯ 7 years 8 months
◯ 7 years 9 months
◯ 7 years 10 months
◯ 7 years 11 months
◯ 8 years
◯ 8 years 1 month
◯ 8 years 2 months
◯ 8 years 3 months
◯ 8 years 4 months
◯ 8 years 5 months
◯ 8 years 6 months
◯ 8 years 7 months
◯ 8 years 8 months
◯ 8 years 9 months
◯ 8 years 10 months
◯ 8 years 11 months
◯ 9 years
◯ 9 years 1 month
◯ 9 years 2 months
◯ 9 years 3 months
◯ 9 years 4 months
◯ 9 years 5 months
◯ 9 years 6 months
◯ 9 years 7 months
◯ 9 years 8 months
◯ 9 years 9 months
◯ 9 years 10 months
◯ 9 years 11 months
◯ 10 years
◯ 10 years 1 month
◯ 10 years 2 months
◯ 10 years 3 months
◯ 10 years 4 months
◯ 10 years 5 months
◯ 10 years 6 months
◯ 10 years 7 months
◯ 10 years 8 months
◯ 10 years 9 months
◯ 10 years 10 months
◯ 10 years 11 months
◯ 11 years
◯ 11 years 1 month
◯ 11 years 2 months
◯ 11 years 3 months
◯ 11 years 4 months
◯ 11 years 5 months
◯ 11 years 6 months
◯ 11 years 7 months
◯ 11 years 8 months
◯ 11 years 9 months
◯ 11 years 10 months
◯ 11 years 11 months
◯ 12 years
◯ 12 years 1 month
◯ 12 years 2 months
◯ 12 years 3 months
◯ 12 years 4 months
◯ 12 years 5 months
◯ 12 years 6 months

◯ 12 years 7 months
◯ 12 years 8 months
◯ 12 years 9 months
◯ 12 years 10 months
◯ 12 years 11 months
◯ 13 years
◯ 13 years 1 month
◯ 13 years 2 months
◯ 13 years 3 months
◯ 13 years 4 months
◯ 13 years 5 months
◯ 13 years 6 months
◯ 13 years 7 months
◯ 13 years 8 months
◯ 13 years 9 months
◯ 13 years 10 months
◯ 13 years 11 months
◯ 14 years
◯ 14 years 1 month
◯ 14 years 2 months
◯ 14 years 3 months
◯ 14 years 4 months
◯ 14 years 5 months
◯ 14 years 6 months
◯ 14 years 7 months
◯ 14 years 8 months
◯ 14 years 9 months
◯ 14 years 10 months
◯ 14 years 11 months
◯ 15 years
◯ 15 years 1 month
◯ 15 years 2 months
◯ 15 years 3 months
◯ 15 years 4 months
◯ 15 years 5 months
◯ 15 years 6 months
◯ 15 years 7 months
◯ 15 years 8 months
◯ 15 years 9 months
◯ 15 years 10 months
◯ 15 years 11 months
◯ 16 years
◯ 16 years 1 month
◯ 16 years 2 month
◯ 16 years 3 months
◯ 16 years 4 months
◯ 16 years 5 months
◯ 16 years 6 months
◯ 16 years 7 months
◯ 16 years 8 months
◯ 16 years 9 months
◯ 16 years 10 months
◯ 16 years 11 months
◯ 17 years
◯ 17 years 1 month
◯ 17 years 2 months
◯ 17 years 3 months
◯ 17 years 4 months
◯ 17 years 5 months
◯ 17 years 6 months
◯ 17 years 7 months
◯ 17 years 8 months
◯ 17 years 9 months
◯ 17 years 10 months
◯ 17 years 11 months
◯ 18 years
◯ 18 years 1 month
◯ 18 years 2 months
◯ 18 years 3 months
◯ 18 years 4 months
◯ 18 years 5 months

	◯ 18 years 6 months<br>◯ 18 years 7 months<br>◯ 18 years 8 months<br>◯ 18 years 9 months<br>◯ 18 years 10 months<br>◯ 18 years 11 months<br>◯ 19 years<br>◯ Over 19 years old
What is THIS diabetic household dog's sex?	◯ Intact male<br>◯ Intact female<br>◯ Neutered male<br>◯ Spayed female
What is THIS diabetic household dog's approximate weight in pounds?	__________________________________
What diet (brand and type) are you feeding THIS diabetic household dog?	__________________________________
Is THIS diabetic household dog receiving insulin?	◯ Yes<br>◯ No
Which type of insulin is THIS diabetic household dog receiving?	◯ NPH / Humulin-N / Novolin-N<br>◯ Lantus / Glargine<br>◯ Vetsulin<br>◯ PZI<br>◯ Levemir / Detemir<br>◯ Other
What is the name of insulin which THIS diabetic household dog is receiving?	__________________________________
How many times a day does THIS household dog receive insulin?	◯ Once daily<br>◯ Twice daily<br>◯ Three times daily

How many units of insulin does THIS household dog receive with each injection?	◯ 1 units<br>◯ 2 units<br>◯ 3 units<br>◯ 4 units<br>◯ 5 units<br>◯ 6 units<br>◯ 7 units<br>◯ 8 units<br>◯ 9 units<br>◯ 10 units<br>◯ 11 units<br>◯ 12 units<br>◯ 13 units<br>◯ 14 units<br>◯ 15 units<br>◯ 16 units<br>◯ 17 units<br>◯ 18 units<br>◯ 19 units<br>◯ 20 units<br>◯ 21 units<br>◯ 22 units<br>◯ 23 units<br>◯ 24 units<br>◯ 25 units<br>◯ 26 units<br>◯ 27 units<br>◯ 28 units<br>◯ 29 units<br>◯ 30 units<br>◯ 31 units<br>◯ 32 units<br>◯ 33 units<br>◯ 34 units<br>◯ 35 units or more
When was THIS diabetic household dog diagnosed with diabetes (approximate if unknown)?	________________________
What is the second diabetic household dog's call name?	________________________
What is THIS diabetic household dog's AKC-registered name?	________________________

What is THIS diabetic household dog's breed?

- ◯ Samoyed
- ◯ Samoyed mix
- ◯ Australian Terrier
- ◯ Australian Terrier mix
- ◯ Pug
- ◯ Pug mix
- ◯ American Eskimo
- ◯ American Eskimo mix
- ◯ Mixed breed
- ◯ Other pure breed
- ◯ German Shepherd
- ◯ German Shepherd mix
- ◯ Golden Retriever
- ◯ Golden Retriever mix
- ◯ American Pit Bull Terrier
- ◯ American Pit Bull Terrier mix
- ◯ Labrador Retriever
- ◯ Labrador Retriever mix

What is THIS diabetic household dog's date of birth?

____

What is THIS diabetic household dog's age today?

- ◯ Less than 6 months old
- ◯ 6 months to 1 year old
- ◯ 1 year 1 month
- ◯ 1 year 2 months
- ◯ 1 year 3 months
- ◯ 1 year 4 months
- ◯ 1 year 5 months
- ◯ 1 year 6 months
- ◯ 1 year 7 months
- ◯ 1 year 8 months
- ◯ 1 year 9 months
- ◯ 1 year 10 months
- ◯ 1 year 11 months
- ◯ 2 years
- ◯ 2 years 1 month
- ◯ 2 years 2 months
- ◯ 2 years 3 months
- ◯ 2 years 4 months
- ◯ 2 years 5 months
- ◯ 2 years 6 months
- ◯ 2 years 7 months
- ◯ 2 years 8 months
- ◯ 2 years 9 months
- ◯ 2 years 10 months
- ◯ 2 years 11 months
- ◯ 3 years
- ◯ 3 years 1 month
- ◯ 3 years 2 months
- ◯ 3 years 3 months
- ◯ 3 years 4 months
- ◯ 3 years 5 months
- ◯ 3 years 6 months
- ◯ 3 years 7 months
- ◯ 3 years 8 months
- ◯ 3 years 9 months
- ◯ 3 years 10 months
- ◯ 3 years 11 months
- ◯ 4 years
- ◯ 4 years 1 month
- ◯ 4 years 2 months
- ◯ 4 years 3 months
- ◯ 4 years 4 months
- ◯ 4 years 5 months
- ◯ 4 years 6 months
- ◯ 4 years 7 months
- ◯ 4 years 8 months
- ◯ 4 years 9 months
- ◯ 4 years 10 months
- ◯ 4 years 11 months
- ◯ 5 years
- ◯ 5 years 1 month
- ◯ 5 years 2 months
- ◯ 5 years 3 months
- ◯ 5 years 4 months
- ◯ 5 years 5 months
- ◯ 5 years 6 months
- ◯ 5 years 7 months
- ◯ 5 years 8 months
- ◯ 5 years 9 months
- ◯ 5 years 10 months
- ◯ 5 years 11 months
- ◯ 6 years
- ◯ 6 years 1 month
- ◯ 6 years 2 months
- ◯ 6 years 3 months
- ◯ 6 years 4 months
- ◯ 6 years 5 months
- ◯ 6 years 6 months
- ◯ 6 years 7 months

- ◯ 6 years 8 months
- ◯ 6 years 9 months
- ◯ 6 years 10 months
- ◯ 6 years 11 months
- ◯ 7 years
- ◯ 7 years 1 month
- ◯ 7 years 2 months
- ◯ 7 years 3 months
- ◯ 7 years 4 months
- ◯ 7 years 5 months
- ◯ 7 years 6 months
- ◯ 7 years 7 months
- ◯ 7 years 8 months
- ◯ 7 years 9 months
- ◯ 7 years 10 months
- ◯ 7 years 11 months
- ◯ 8 years
- ◯ 8 years 1 month
- ◯ 8 years 2 months
- ◯ 8 years 3 months
- ◯ 8 years 4 months
- ◯ 8 years 5 months
- ◯ 8 years 6 months
- ◯ 8 years 7 months
- ◯ 8 years 8 months
- ◯ 8 years 9 months
- ◯ 8 years 10 months
- ◯ 8 years 11 months
- ◯ 9 years
- ◯ 9 years 1 month
- ◯ 9 years 2 months
- ◯ 9 years 3 months
- ◯ 9 years 4 months
- ◯ 9 years 5 months
- ◯ 9 years 6 months
- ◯ 9 years 7 months
- ◯ 9 years 8 months
- ◯ 9 years 9 months
- ◯ 9 years 10 months
- ◯ 9 years 11 months
- ◯ 10 years
- ◯ 10 years 1 month
- ◯ 10 years 2 months
- ◯ 10 years 3 months
- ◯ 10 years 4 months
- ◯ 10 years 5 months
- ◯ 10 years 6 months
- ◯ 10 years 7 months
- ◯ 10 years 8 months
- ◯ 10 years 9 months
- ◯ 10 years 10 months
- ◯ 10 years 11 months
- ◯ 11 years
- ◯ 11 years 1 month
- ◯ 11 years 2 months
- ◯ 11 years 3 months
- ◯ 11 years 4 months
- ◯ 11 years 5 months
- ◯ 11 years 6 months
- ◯ 11 years 7 months
- ◯ 11 years 8 months
- ◯ 11 years 9 months
- ◯ 11 years 10 months
- ◯ 11 years 11 months
- ◯ 12 years
- ◯ 12 years 1 month
- ◯ 12 years 2 months
- ◯ 12 years 3 months
- ◯ 12 years 4 months
- ◯ 12 years 5 months
- ◯ 12 years 6 months

◯ 12 years 7 months
◯ 12 years 8 months
◯ 12 years 9 months
◯ 12 years 10 months
◯ 12 years 11 months
◯ 13 years
◯ 13 years 1 month
◯ 13 years 2 months
◯ 13 years 3 months
◯ 13 years 4 months
◯ 13 years 5 months
◯ 13 years 6 months
◯ 13 years 7 months
◯ 13 years 8 months
◯ 13 years 9 months
◯ 13 years 10 months
◯ 13 years 11 months
◯ 14 years
◯ 14 years 1 month
◯ 14 years 2 months
◯ 14 years 3 months
◯ 14 years 4 months
◯ 14 years 5 months
◯ 14 years 6 months
◯ 14 years 7 months
◯ 14 years 8 months
◯ 14 years 9 months
◯ 14 years 10 months
◯ 14 years 11 months
◯ 15 years
◯ 15 years 1 month
◯ 15 years 2 months
◯ 15 years 3 months
◯ 15 years 4 months
◯ 15 years 5 months
◯ 15 years 6 months
◯ 15 years 7 months
◯ 15 years 8 months
◯ 15 years 9 months
◯ 15 years 10 months
◯ 15 years 11 months
◯ 16 years
◯ 16 years 1 month
◯ 16 years 2 month
◯ 16 years 3 months
◯ 16 years 4 months
◯ 16 years 5 months
◯ 16 years 6 months
◯ 16 years 7 months
◯ 16 years 8 months
◯ 16 years 9 months
◯ 16 years 10 months
◯ 16 years 11 months
◯ 17 years
◯ 17 years 1 month
◯ 17 years 2 months
◯ 17 years 3 months
◯ 17 years 4 months
◯ 17 years 5 months
◯ 17 years 6 months
◯ 17 years 7 months
◯ 17 years 8 months
◯ 17 years 9 months
◯ 17 years 10 months
◯ 17 years 11 months
◯ 18 years
◯ 18 years 1 month
◯ 18 years 2 months
◯ 18 years 3 months
◯ 18 years 4 months
◯ 18 years 5 months

	◯ 18 years 6 months<br>◯ 18 years 7 months<br>◯ 18 years 8 months<br>◯ 18 years 9 months<br>◯ 18 years 10 months<br>◯ 18 years 11 months<br>◯ 19 years<br>◯ Over 19 years old
What is THIS diabetic household dog's sex?	◯ Intact male<br>◯ Intact female<br>◯ Neutered male<br>◯ Spayed female
What is THIS diabetic household dog's approximate weight in pounds?	__________________________________
What diet (brand and type) are you feeding THIS diabetic household dog?	__________________________________
Is THIS diabetic household dog receiving insulin?	◯ Yes<br>◯ No
Which type of insulin is THIS diabetic household dog receiving?	◯ NPH / Humulin-N / Novolin-N<br>◯ Lantus / Glargine<br>◯ Vetsulin<br>◯ PZI<br>◯ Levemir / Detemir<br>◯ Other
What is the name of insulin which THIS diabetic household dog is receiving?	__________________________________
How many times a day does THIS household dog receive insulin?	◯ Once daily<br>◯ Twice daily<br>◯ Three times daily

How many units of insulin does THIS household dog receive with each injection?

◯ 1 units
◯ 2 units
◯ 3 units
◯ 4 units
◯ 5 units
◯ 6 units
◯ 7 units
◯ 8 units
◯ 9 units
◯ 10 units
◯ 11 units
◯ 12 units
◯ 13 units
◯ 14 units
◯ 15 units
◯ 16 units
◯ 17 units
◯ 18 units
◯ 19 units
◯ 20 units
◯ 21 units
◯ 22 units
◯ 23 units
◯ 24 units
◯ 25 units
◯ 26 units
◯ 27 units
◯ 28 units
◯ 29 units
◯ 30 units
◯ 31 units
◯ 32 units
◯ 33 units
◯ 34 units
◯ 35 units or more

When was THIS diabetic household dog diagnosed with diabetes (approximate if unknown)?

____

What is the third diabetic household dog's call name?

____

What is THIS diabetic household dog's AKC-registered name?

____

What is THIS diabetic household dog's breed?

- ◯ Samoyed
- ◯ Samoyed mix
- ◯ Australian Terrier
- ◯ Australian Terrier mix
- ◯ Pug
- ◯ Pug mix
- ◯ American Eskimo
- ◯ American Eskimo mix
- ◯ Mixed breed
- ◯ Other pure breed
- ◯ German Shepherd
- ◯ German Shepherd mix
- ◯ Golden Retriever
- ◯ Golden Retriever mix
- ◯ American Pit Bull Terrier
- ◯ American Pit Bull Terrier mix
- ◯ Labrador Retriever
- ◯ Labrador Retriever mix

What is THIS diabetic household dog's date of birth?

____

What is THIS diabetic household dog's age today?

◯ Less than 6 months old
◯ 6 months to 1 year old
◯ 1 year 1 month
◯ 1 year 2 months
◯ 1 year 3 months
◯ 1 year 4 months
◯ 1 year 5 months
◯ 1 year 6 months
◯ 1 year 7 months
◯ 1 year 8 months
◯ 1 year 9 months
◯ 1 year 10 months
◯ 1 year 11 months
◯ 2 years
◯ 2 years 1 month
◯ 2 years 2 months
◯ 2 years 3 months
◯ 2 years 4 months
◯ 2 years 5 months
◯ 2 years 6 months
◯ 2 years 7 months
◯ 2 years 8 months
◯ 2 years 9 months
◯ 2 years 10 months
◯ 2 years 11 months
◯ 3 years
◯ 3 years 1 month
◯ 3 years 2 months
◯ 3 years 3 months
◯ 3 years 4 months
◯ 3 years 5 months
◯ 3 years 6 months
◯ 3 years 7 months
◯ 3 years 8 months
◯ 3 years 9 months
◯ 3 years 10 months
◯ 3 years 11 months
◯ 4 years
◯ 4 years 1 month
◯ 4 years 2 months
◯ 4 years 3 months
◯ 4 years 4 months
◯ 4 years 5 months
◯ 4 years 6 months
◯ 4 years 7 months
◯ 4 years 8 months
◯ 4 years 9 months
◯ 4 years 10 months
◯ 4 years 11 months
◯ 5 years
◯ 5 years 1 month
◯ 5 years 2 months
◯ 5 years 3 months
◯ 5 years 4 months
◯ 5 years 5 months
◯ 5 years 6 months
◯ 5 years 7 months
◯ 5 years 8 months
◯ 5 years 9 months
◯ 5 years 10 months
◯ 5 years 11 months
◯ 6 years
◯ 6 years 1 month
◯ 6 years 2 months
◯ 6 years 3 months
◯ 6 years 4 months
◯ 6 years 5 months
◯ 6 years 6 months
◯ 6 years 7 months

◯ 6 years 8 months
◯ 6 years 9 months
◯ 6 years 10 months
◯ 6 years 11 months
◯ 7 years
◯ 7 years 1 month
◯ 7 years 2 months
◯ 7 years 3 months
◯ 7 years 4 months
◯ 7 years 5 months
◯ 7 years 6 months
◯ 7 years 7 months
◯ 7 years 8 months
◯ 7 years 9 months
◯ 7 years 10 months
◯ 7 years 11 months
◯ 8 years
◯ 8 years 1 month
◯ 8 years 2 months
◯ 8 years 3 months
◯ 8 years 4 months
◯ 8 years 5 months
◯ 8 years 6 months
◯ 8 years 7 months
◯ 8 years 8 months
◯ 8 years 9 months
◯ 8 years 10 months
◯ 8 years 11 months
◯ 9 years
◯ 9 years 1 month
◯ 9 years 2 months
◯ 9 years 3 months
◯ 9 years 4 months
◯ 9 years 5 months
◯ 9 years 6 months
◯ 9 years 7 months
◯ 9 years 8 months
◯ 9 years 9 months
◯ 9 years 10 months
◯ 9 years 11 months
◯ 10 years
◯ 10 years 1 month
◯ 10 years 2 months
◯ 10 years 3 months
◯ 10 years 4 months
◯ 10 years 5 months
◯ 10 years 6 months
◯ 10 years 7 months
◯ 10 years 8 months
◯ 10 years 9 months
◯ 10 years 10 months
◯ 10 years 11 months
◯ 11 years
◯ 11 years 1 month
◯ 11 years 2 months
◯ 11 years 3 months
◯ 11 years 4 months
◯ 11 years 5 months
◯ 11 years 6 months
◯ 11 years 7 months
◯ 11 years 8 months
◯ 11 years 9 months
◯ 11 years 10 months
◯ 11 years 11 months
◯ 12 years
◯ 12 years 1 month
◯ 12 years 2 months
◯ 12 years 3 months
◯ 12 years 4 months
◯ 12 years 5 months
◯ 12 years 6 months

◯ 12 years 7 months
◯ 12 years 8 months
◯ 12 years 9 months
◯ 12 years 10 months
◯ 12 years 11 months
◯ 13 years
◯ 13 years 1 month
◯ 13 years 2 months
◯ 13 years 3 months
◯ 13 years 4 months
◯ 13 years 5 months
◯ 13 years 6 months
◯ 13 years 7 months
◯ 13 years 8 months
◯ 13 years 9 months
◯ 13 years 10 months
◯ 13 years 11 months
◯ 14 years
◯ 14 years 1 month
◯ 14 years 2 months
◯ 14 years 3 months
◯ 14 years 4 months
◯ 14 years 5 months
◯ 14 years 6 months
◯ 14 years 7 months
◯ 14 years 8 months
◯ 14 years 9 months
◯ 14 years 10 months
◯ 14 years 11 months
◯ 15 years
◯ 15 years 1 month
◯ 15 years 2 months
◯ 15 years 3 months
◯ 15 years 4 months
◯ 15 years 5 months
◯ 15 years 6 months
◯ 15 years 7 months
◯ 15 years 8 months
◯ 15 years 9 months
◯ 15 years 10 months
◯ 15 years 11 months
◯ 16 years
◯ 16 years 1 month
◯ 16 years 2 month
◯ 16 years 3 months
◯ 16 years 4 months
◯ 16 years 5 months
◯ 16 years 6 months
◯ 16 years 7 months
◯ 16 years 8 months
◯ 16 years 9 months
◯ 16 years 10 months
◯ 16 years 11 months
◯ 17 years
◯ 17 years 1 month
◯ 17 years 2 months
◯ 17 years 3 months
◯ 17 years 4 months
◯ 17 years 5 months
◯ 17 years 6 months
◯ 17 years 7 months
◯ 17 years 8 months
◯ 17 years 9 months
◯ 17 years 10 months
◯ 17 years 11 months
◯ 18 years
◯ 18 years 1 month
◯ 18 years 2 months
◯ 18 years 3 months
◯ 18 years 4 months
◯ 18 years 5 months

	◯ 18 years 6 months<br>◯ 18 years 7 months<br>◯ 18 years 8 months<br>◯ 18 years 9 months<br>◯ 18 years 10 months<br>◯ 18 years 11 months<br>◯ 19 years<br>◯ Over 19 years old
What is THIS diabetic household dog's sex?	◯ Intact male<br>◯ Intact female<br>◯ Neutered male<br>◯ Spayed female
What is THIS diabetic household dog's approximate weight in pounds?	________________________
What diet (brand and type) are you feeding THIS diabetic household dog?	________________________
Is THIS diabetic household dog receiving insulin?	◯ Yes<br>◯ No
Which type of insulin is THIS diabetic household dog receiving?	◯ NPH / Humulin-N / Novolin-N<br>◯ Lantus / Glargine<br>◯ Vetsulin<br>◯ PZI<br>◯ Levemir / Detemir<br>◯ Other
What is the name of insulin which THIS diabetic household dog is receiving?	________________________
How many times a day does THIS household dog receive insulin?	◯ Once daily<br>◯ Twice daily<br>◯ Three times daily

How many units of insulin does THIS household dog receive with each injection?

◯ 1 units
◯ 2 units
◯ 3 units
◯ 4 units
◯ 5 units
◯ 6 units
◯ 7 units
◯ 8 units
◯ 9 units
◯ 10 units
◯ 11 units
◯ 12 units
◯ 13 units
◯ 14 units
◯ 15 units
◯ 16 units
◯ 17 units
◯ 18 units
◯ 19 units
◯ 20 units
◯ 21 units
◯ 22 units
◯ 23 units
◯ 24 units
◯ 25 units
◯ 26 units
◯ 27 units
◯ 28 units
◯ 29 units
◯ 30 units
◯ 31 units
◯ 32 units
◯ 33 units
◯ 34 units
◯ 35 units or more

When was THIS diabetic household dog diagnosed with diabetes (approximate if unknown)?

____

Miscellaneous information

Are you aware of a deceased diabetic dog that was directly related to the dog in this survey?

◯ Yes
◯ No

Would you like to share information regarding this deceased diabetic dog?

◯ Yes
◯ No

What was this deceased diabetic dog's call name?

____

Did this deceased diabetic dog have an AKC-registered name?

◯ Yes
◯ No

What was this deceased diabetic dog's AKC-registered name?

____

What was this deceased diabetic dog's breed?	◯ Samoyed<br>◯ Samoyed mix<br>◯ Australian Terrier<br>◯ Australian Terrier mix<br>◯ Pug<br>◯ Pug mix<br>◯ American Eskimo<br>◯ American Eskimo mix<br>◯ Mixed breed<br>◯ Other pure breed<br>◯ German Shepherd<br>◯ German Shepherd mix<br>◯ Golden Retriever<br>◯ Golden Retriever mix<br>◯ American Pit Bull Terrier<br>◯ American Pit Bull Terrier mix<br>◯ Labrador Retriever<br>◯ Labrador Retriever mix
What was this deceased diabetic dog's breed?	__________________________
What was this deceased diabetic dog's sex?	◯ Intact male<br>◯ Intact female<br>◯ Neutered male<br>◯ Spayed female
What was this deceased diabetic dog's date of birth (approximate if unknown)?	__________________________
When did this diabetic dog become deceased (approximate if unknown)?	__________________________
When was this deceased diabetic dog diagnosed with diabetes (approximate if unknown)?	__________________________
Approximately how much did this deceased diabetic dog weigh in pounds (at adulthood)?	__________________________
Which type of insulin did this deceased diabetic dog receive?	◯ NPH / Humulin-N / Novolin-N<br>◯ Lantus / Glargine<br>◯ Vetsulin<br>◯ PZI<br>◯ Levemir / Detemir<br>◯ Other<br>◯ Unknown
What is the name of the insulin that this deceased diabetic dog received?	__________________________
How many times a day did this deceased diabetic dog receive insulin?	◯ Once daily<br>◯ Twice daily<br>◯ Three times daily<br>◯ Unknown

How many units of insulin did this deceased diabetic dog receive with each injection?

- ◯ 1 units
- ◯ 2 units
- ◯ 3 units
- ◯ 4 units
- ◯ 5 units
- ◯ 6 units
- ◯ 7 units
- ◯ 8 units
- ◯ 9 units
- ◯ 10 units
- ◯ 11 units
- ◯ 12 units
- ◯ 13 units
- ◯ 14 units
- ◯ 15 units
- ◯ 16 units
- ◯ 17 units
- ◯ 18 units
- ◯ 19 units
- ◯ 20 units
- ◯ 21 units
- ◯ 22 units
- ◯ 23 units
- ◯ 24 units
- ◯ 25 units
- ◯ 26 units
- ◯ 27 units
- ◯ 28 units
- ◯ 29 units
- ◯ 30 units
- ◯ 31 units
- ◯ 32 units
- ◯ 33 units
- ◯ 34 units
- ◯ 35 units or more
- ◯ Unknown

Your input is greatly appreciated. Please provide us with any comments or feedback (optional):

____
