# Supplementary material for: Seasonality and geography of diabetes mellitus in United States of America dogs
Source: PLoS One. 2022 Aug 5;17(8):e0272297. doi: 10.1371/journal.pone.0272297 (PMC9355170; doi:10.1371/journal.pone.0272297)
Supplement: S2 Appendix — (PDF) [file pone.0272297.s002.pdf]

Appendix 2

Academic veterinary hospitals and private veterinary referral hospitals, which promoted the online survey:

1. University of Alaska Veterinary Medicine Program (Dr. Arleigh Reynolds) – Fairbanks, AK 99775
2. Angell Animal Medical Center (Dr. Megan Whelan) – Boston, MA 02130
3. Animal Specialty Group (Dr. Rhonda Schulman) – Los Angeles, CA 90039
4. Auburn University College of Veterinary Medicine (Dr. Ellen Behrend) – Auburn, AL 36849
5. Blue Pearl Veterinary Partners (Brenda Fulcher) – Tampa, Florida 33614
6. University of California Davis School of Veterinary Medicine (Dr. Chen Gilor, Dr. Kate Hopper, Dr. Carrie Palm) – Davis, CA 95616
7. Charleston Veterinary Referral Center (Dr. Derek Duval) – Charleston, SC 29414
8. Colorado State University Veterinary Teaching Hospital (Dr. Craig Webb) – Fort Collins, CO 80525
9. Cornell University College of Veterinary Medicine (Dr. Daniel Fletcher) – Ithaca, NY 14853
10. Dogs and Cats Veterinary Referral (Dr. William Pullen) – Bowie, MD 20715
11. University of Florida College of Veterinary Medicine (Dr. Allison O'Kell, Dr. Richard Hill) – Gainesville, FL 32608
12. Four Seasons Veterinary Specialists (Dr. Lauren Prause) – Loveland, CO 80538
13. University of Georgia College of Veterinary Medicine (Dr. Cynthia Ward) – Athens, GA 30602
14. University of Illinois College of Veterinary Medicine (Dr. Heidi Phillips) – Urbana, IL 61802
15. IndyVet (Dr. Tracey Gillespie) – Indianapolis, IN 46203
16. Kansas State University College of Veterinary Medicine (Dr. Thomas Schermerhorn) – Manhattan, KS 66502
17. Louisiana State University College of Veterinary Medicine (Dr. Frederic Gaschen) – Baton Rouge, LA 70803
18. Metropolitan Veterinary Association (Dr. James Dougherty) – Norristown, PA 19403
19. Michigan State University Veterinary Medical Center (Dr. Matthew Beal) – East Lansing, MI 48824
20. Midwestern University (Dr. Angela Mexas) – Glendale, AZ 85308

21. University of Minnesota College of Veterinary Medicine (Dr. Eva Furrow & Dr. David Polzin) – St. Paul, MN 55108
22. Mississippi State University College of Veterinary Medicine (Dr. Patty Lathan) – Mississippi State, MS 39762
23. University of Missouri Veterinary Health Center (Dr. Leah Cohn) – Columbia, MO 65211
24. New England Animal Medical Center (Dr. Patricia Walters) – Bridgewater, MA 02379
25. North Carolina State University College of Veterinary Medicine (Dr. Shelly Vaden) – Raleigh, NC 27606
26. The Ohio State University Veterinary Medical Center (Dr. Cathy Langston) – Columbus, OH 43210
27. Oklahoma State University Boren Veterinary Medical Hospital (Dr. Mark Neer, Dr. Daniel Burba) – Stillwater, OK 74078
28. Oregon State University Carlson College of Veterinary Medicine (Dr. Helio de Morais, Dr. Jana Gordon) – Corvallis, OR 97331
29. University of Pennsylvania School of Veterinary Medicine (Dr. Rebecka Hess)
30. Purdue University College of Veterinary Medicine (Dr. Nolie Parnell) – West Lafayette, IN 47907
31. Seattle Veterinary Specialists (Dr. Frances Hurrell) – Seattle, WA 98104
32. Southeast Veterinary Oncology and Internal Medicine (Dr. Abigail Bertalan) – Orange Park, FL 32073
33. Specialized Veterinary Services (Dr. Melissa Tollett) – Fort Myers, FL 33912
34. University of Tennessee College of Veterinary Medicine (Dr. Jacqueline Whittemore) – Knoxville, TN 37996
35. Texas A&M College of Veterinary Medicine (Dr. Jorg Steiner) – College Station, TX 77843
36. Tufts Cummings School of Veterinary Medicine (Dr. Elizabeth Rozanski) – North Grafton, MA 01536
37. Tuskegee University Veterinary Medical Teaching Hospital (Dr. Jeannine Bellamy) – Tuskegee, AL 36088
38. Veterinary Specialists and Emergency Services (Dr. Michael Koch) – Brighton, NY 14623
39. Virginia-Maryland College of Veterinary Medicine (Dr. David Panciera) – Blacksburg, VA 24060
40. Washington State University College of Veterinary Medicine (Dr. Rance Sellon) – Pullman, WA 99164

41. Western University of Health Sciences: College of Veterinary Medicine (Dr. Linda Kidd) – Pomona, CA 91766

42. WestVet (Dr. Daniel Hume) – Garden City, ID 83714

43. University of Wisconsin School of Veterinary Medicine (Dr. Lauren Trepanier) – Madison, WI 53706
