# Supplementary material for: Seasonality and geography of diabetes mellitus in United States of America dogs
Source: PLoS One. 2022 Aug 5;17(8):e0272297. doi: 10.1371/journal.pone.0272297 (PMC9355170; doi:10.1371/journal.pone.0272297)
Supplement: S3 Appendix — (PDF) [file pone.0272297.s003.pdf]

DM Prevalence and Incidence

Project Home | Project Setup | Online Designer | Data Dictionary | Codebook

Data Dictionary Codebook

01/08/2020 3:46pm

Collapse all instruments

#	Variable / Field Name	Field Label *Field Note*	Field Attributes (Field Type, Validation, Choices, Calculations, etc.)
Instrument: **Diabetes Mellitus Survey** (diabetes_mellitus_survey) Enabled as survey			Collapse
1	record_id	Record ID	text
2	first_name	Section Header: *Your (Owner) Contact Information*<br>First Name	text, Required, Identifier
3	last_name	Last Name	text, Required, Identifier
4	phone_number	Phone Number	text (phone), Identifier
5	email	Email Address	text (email), Identifier
6	own_add	State of Address:	dropdown<br>0 AK<br>1 AL<br>2 AR<br>3 AZ<br>4 CA<br>5 CO<br>6 CT<br>7 DE<br>8 FL<br>9 GA<br>10 HI<br>11 IA<br>12 ID<br>13 IL<br>14 IN<br>15 KS<br>16 KY<br>17 LA<br>18 MA<br>19 ME

			20 MD<br>21 MI<br>22 MN<br>23 MO<br>24 MS<br>25 MT<br>26 NC<br>27 ND<br>28 NE<br>29 NH<br>30 NJ<br>31 NM<br>32 NV<br>33 NY<br>34 OH<br>35 OK<br>36 OK<br>37 OR<br>38 PA<br>39 RI<br>40 SC<br>41 SD<br>42 TN<br>43 TX<br>44 UT<br>45 VT<br>46 VA<br>47 WA<br>48 WI<br>49 WV<br>50 WY
7	surv_date	Today's Date	text (date_mdy)
8	call_name	Section Header: *Your Pet's Information*<br>What is your pet's call name?	text, Required
9	akc_yn	Does your pet have an AKC-registered name?	yesno, Required<br>1 Yes<br>0 No
10	akc_name<br>Show the field ONLY if:<br>[akc_yn] = '1'	What is your pet's AKC-registered name?	text
11	breed_type	What is your pet's breed?	dropdown, Required<br>1 Samoyed<br>2 Samoyed mix<br>3 Australian Terrier

			4 Australian Terrier mix<br>5 Pug<br>6 Pug mix<br>7 American Eskimo<br>8 American Eskimo mix<br>9 Mixed breed<br>10 Other pure breed<br>11 German Shepherd<br>12 German Shepherd mix<br>13 Golden Retriever<br>14 Golden Retriever mix<br>15 American Pit Bull Terrier<br>16 American Pit Bull Terrier mix<br>17 Labrador Retriever<br>18 Labrador Retriever mix
12	mixedbreed<br>Show the field ONLY if:<br>[breed_type] = 10	What is your pet's breed?	text
13	dob	What is your pet's date of birth (approximate if unknown)?	text (date_mdy, Min: 1992-01-01, Max: 2022-01-01), Required, Identifier
14	pet_age	What is your pet's age today?	dropdown<br>0 Less than 6 months old<br>1 6 months to 1 year old<br>2 1 year 1 month<br>3 1 year 2 months<br>4 1 year 3 months<br>5 1 year 4 months<br>6 1 year 5 months<br>7 1 year 6 months<br>8 1 year 7 months<br>9 1 year 8 months<br>10 1 year 9 months<br>11 1 year 10 months<br>12 1 year 11 months<br>13 2 years<br>14 2 years 1 month<br>15 2 years 2 months<br>16 2 years 3 months<br>17 2 years 4 months<br>18 2 years 5 months<br>19 2 years 6 months<br>20 2 years 7 months<br>21 2 years 8 months<br>22 2 years 9 months

23	2 years 10 months
24	2 years 11 months
25	3 years
26	3 years 1 month
27	3 years 2 months
28	3 years 3 months
29	3 years 4 months
30	3 years 5 months
31	3 years 6 months
32	3 years 7 months
33	3 years 8 months
34	3 years 9 months
35	3 years 10 months
36	3 years 11 months
37	4 years
38	4 years 1 month
39	4 years 2 months
40	4 years 3 months
41	4 years 4 months
42	4 years 5 months
43	4 years 6 months
44	4 years 7 months
45	4 years 8 months
46	4 years 9 months
47	4 years 10 months
48	4 years 11 months
49	5 years
50	5 years 1 month
51	5 years 2 months
52	5 years 3 months
53	5 years 4 months
54	5 years 5 months
55	5 years 6 months
56	5 years 7 months
57	5 years 8 months
58	5 years 9 months
59	5 years 10 months
60	5 years 11 months
61	6 years
62	6 years 1 month
63	6 years 2 months
64	6 years 3 months
65	6 years 4 months
66	6 years 5 months

67	6 years 6 months
68	6 years 7 months
69	6 years 8 months
70	6 years 9 months
71	6 years 10 months
72	6 years 11 months
73	7 years
74	7 years 1 month
75	7 years 2 months
76	7 years 3 months
77	7 years 4 months
78	7 years 5 months
79	7 years 6 months
80	7 years 7 months
81	7 years 8 months
82	7 years 9 months
83	7 years 10 months
84	7 years 11 months
85	8 years
86	8 years 1 month
87	8 years 2 months
88	8 years 3 months
89	8 years 4 months
90	8 years 5 months
91	8 years 6 months
92	8 years 7 months
93	8 years 8 months
94	8 years 9 months
95	8 years 10 months
96	8 years 11 months
97	9 years
98	9 years 1 month
99	9 years 2 months
100	9 years 3 months
101	9 years 4 months
102	9 years 5 months
103	9 years 6 months
104	9 years 7 months
105	9 years 8 months
106	9 years 9 months
107	9 years 10 months
108	9 years 11 months
109	10 years

110	10 years 1 month
111	10 years 2 months
112	10 years 3 months
113	10 years 4 months
114	10 years 5 months
115	10 years 6 months
116	10 years 7 months
117	10 years 8 months
118	10 years 9 months
119	10 years 10 months
120	10 years 11 months
121	11 years
122	11 years 1 month
123	11 years 2 months
124	11 years 3 months
125	11 years 4 months
126	11 years 5 months
127	11 years 6 months
128	11 years 7 months
129	11 years 8 months
130	11 years 9 months
131	11 years 10 months
132	11 years 11 months
133	12 years
134	12 years 1 month
135	12 years 2 months
136	12 years 3 months
137	12 years 4 months
138	12 years 5 months
139	12 years 6 months
140	12 years 7 months
141	12 years 8 months
142	12 years 9 months
143	12 years 10 months
144	12 years 11 months
145	13 years
146	13 years 1 month
147	13 years 2 months
148	13 years 3 months
149	13 years 4 months
150	13 years 5 months
151	13 years 6 months
152	13 years 7 months

153	13 years 8 months
154	13 years 9 months
155	13 years 10 months
156	13 years 11 months
157	14 years
158	14 years 1 month
159	14 years 2 months
160	14 years 3 months
161	14 years 4 months
162	14 years 5 months
163	14 years 6 months
164	14 years 7 months
165	14 years 8 months
166	14 years 9 months
167	14 years 10 months
168	14 years 11 months
169	15 years
170	15 years 1 month
171	15 years 2 months
172	15 years 3 months
173	15 years 4 months
174	15 years 5 months
175	15 years 6 months
176	15 years 7 months
177	15 years 8 months
178	15 years 9 months
179	15 years 10 months
180	15 years 11 months
181	16 years
182	16 years 1 month
183	16 years 2 month
184	16 years 3 months
185	16 years 4 months
186	16 years 5 months
187	16 years 6 months
188	16 years 7 months
189	16 years 8 months
190	16 years 9 months
191	16 years 10 months
192	16 years 11 months
193	17 years
194	17 years 1 month
195	17 years 2 months

			196 17 years 3 months<br>197 17 years 4 months<br>198 17 years 5 months<br>199 17 years 6 months<br>200 17 years 7 months<br>201 17 years 8 months<br>202 17 years 9 months<br>203 17 years 10 months<br>204 17 years 11 months<br>205 18 years<br>206 18 years 1 month<br>207 18 years 2 months<br>208 18 years 3 months<br>209 18 years 4 months<br>210 18 years 5 months<br>211 18 years 6 months<br>212 18 years 7 months<br>213 18 years 8 months<br>214 18 years 9 months<br>215 18 years 10 months<br>216 18 years 11 months<br>217 19 years<br>218 Over 19 years old
15	neut_stat	What is your pet's sex?	dropdown<br>1 Intact male<br>2 Intact female<br>3 Neutered male<br>4 Spayed female
16	pet_wt	What is your dog's approximate weight in pounds?	text (number)
17	diet	What diet (brand and/or type) are you feeding your dog?	text
18	dx_dm	Section Header: *Your Pet's Health Status*<br>Is your pet diabetic?	yesno, Required<br>1 Yes<br>0 No
19	ins_ans<br>Show the field ONLY if:<br>[dx_dm] = '1'	Is your dog receiving insulin?	yesno, Required<br>1 Yes<br>0 No
20	ins_type<br>Show the field ONLY if:<br>[ins_ans] = '1'	Which type of insulin is your dog receiving?	dropdown, Required<br>0 NPH / Humulin-N / Novolin-N<br>1 Lantus / Glargine<br>2 Vetsulin<br>3 PZI<br>4 Levemir / Detemir<br>5 Other

21	instype_other<br>Show the field ONLY if:<br>[ins_type] = '5'	What is the name of insulin which your dog is receiving?	text, Required
22	ins_freq<br>Show the field ONLY if:<br>[ins_ans] = '1'	How many times a day does your dog receive insulin?	dropdown<br>0 Once daily<br>1 Twice daily<br>2 Three times daily
23	ins_units<br>Show the field ONLY if:<br>[ins_ans] = '1'	How many units of insulin does your dog receive with each injection?	dropdown<br>1 1 units<br>2 2 units<br>3 3 units<br>4 4 units<br>5 5 units<br>6 6 units<br>7 7 units<br>8 8 units<br>9 9 units<br>10 10 units<br>11 11 units<br>12 12 units<br>13 13 units<br>14 14 units<br>15 15 units<br>16 16 units<br>17 17 units<br>18 18 units<br>19 19 units<br>20 20 units<br>21 21 units<br>22 22 units<br>23 23 units<br>24 24 units<br>25 25 units<br>26 26 units<br>27 27 units<br>28 28 units<br>29 29 units<br>30 30 units<br>31 31 units<br>32 32 units<br>33 33 units<br>34 34 units<br>35 35 units or more
24	dm_dxdate	When was your dog diagnosed with diabetes (approximate if	text (date_mdy, Min: 2000-01-01, Max: 2017-01-01)

	Show the field ONLY if: [dx_dm] = '1'	unknown)?	
25	vet_name<br>Show the field ONLY if: [dx_dm] = '1'	What is your veterinarian's name (if known)?	text
26	vet_phone<br>Show the field ONLY if: [dx_dm] = '1'	What is your veterinarian's phone number (if known)?	text (phone)
27	dm_cs<br>Show the field ONLY if: [dx_dm] = '1'	What were the clinical signs that you noticed when your dog was diagnosed with diabetes (if recallable)?	checkbox<br>1 dm_cs___1 Increased thirst or urination<br>2 dm_cs___2 Increased hunger<br>3 dm_cs___3 Weight loss
28	hypo_dx	Has your dog been diagnosed with hypothyroidism?	yesno<br>1 Yes<br>0 No
29	hypo_tx<br>Show the field ONLY if: [hypo_dx] = 1	Is your dog being treated with thyroid supplementation?	yesno<br>1 Yes<br>0 No
30	add_dx	Has your dog been diagnosed with Addison's disease (hypoadrenocorticism)?	yesno<br>1 Yes<br>0 No
31	add_tx<br>Show the field ONLY if: [add_dx] = 1	Is your dog currently being treated with any of the following medications?	checkbox<br>0 add_tx___0 Prednisone<br>1 add_tx___1 DOCP<br>2 add_tx___2 Prednisone and DOCP<br>3 add_tx___3 Fludricortisone (e.g. Florinef)
32	pred_timing<br>Show the field ONLY if: [add_tx(0)] = "1" or [add_tx(2)] = "1"	Was the prednisone given before or after the diagnosis of diabetes?	checkbox<br>0 pred_timing___0 Before diagnosis of diabetes<br>1 pred_timing___1 After diagnosis of diabetes
33	preddose<br>Show the field ONLY if: [add_tx(0)] = "1" or [add_tx(2)] = "1"	How much prednisone (in mg) is given per dose (each time you give the medication)?	text
34	preddose_freq<br>Show the field ONLY if: [add_tx(0)] = "1" or [add_tx(2)] = "1"	How many times per day do you give this prednisone dose?	dropdown<br>0 Once daily<br>1 Twice daily<br>2 Other
35	conc_dx	Does your dog have any other concurrent illnesses?	yesno<br>1 Yes<br>0 No
36	conc_illness<br>Show the field ONLY if: [conc_dx] = 1	Please list the concurrent conditions/illnesses.	text
37	breeder_yn	Did you obtain your pet from a breeder?	yesno

			1 Yes<br>0 No
38	breeder_name<br>Show the field ONLY if:<br>[breeder_yn] = 1	What is your pet's breeder's name (if known)?	text
39	breeder_phone<br>Show the field ONLY if:<br>[breeder_yn] = 1	What is your pet's breeder's phone number (if known)?	text (phone)
40	breeder_email<br>Show the field ONLY if:<br>[breeder_yn] = 1	What is your pet's breeder's email?	text (email)
41	rescue_yn<br>Show the field ONLY if:<br>[breeder_yn] = 0	Was your dog obtained from a rescue group?	yesno<br>1 Yes<br>0 No
42	petrescue<br>Show the field ONLY if:<br>[rescue_yn] = 1	What is the name of your dog's rescue?	text
43	petmom_yn	Section Header: *Your Pet's Relatives*<br>Are you in contact with the owner of your dog's mother?	yesno<br>1 Yes<br>0 No
44	petmom_owner<br>Show the field ONLY if:<br>[petmom_yn] = 1	Who is the owner of your dog's mother (if known)?	text
45	petmom_oemail<br>Show the field ONLY if:<br>[petmom_yn] = 1	What is this owner's email address (if known)?	text (email)
46	petmom_name<br>Show the field ONLY if:<br>[petmom_yn] = 1	What is the name of your dog's mother (if known)?	text
47	petmom_vit<br>Show the field ONLY if:<br>[petmom_yn] = 1	Is your dog's mother still alive (if known)?	dropdown<br>0 Yes<br>1 No<br>2 Unsure
48	petdad_yn	Are you in contact with the owner of your dog's father?	yesno<br>1 Yes<br>0 No
49	petdad_owner<br>Show the field ONLY if:<br>[petdad_yn] = 1	Who is the owner of your dog's father (if known)?	text
50	petdad_email<br>Show the field ONLY if:<br>[petdad_yn] = 1	What is this owner's email address (if known)?	text (email)
51	petdad_name<br>Show the field ONLY if:<br>[petdad_yn] = 1	What is the name of your dog's father (if known)?	text
52	petdad_vit	Is your dog's father still alive?	dropdown

	Show the field ONLY if: [petdad_yn] = 1		0 Yes<br>1 No<br>2 Unsure
53	petlit_yn	Does your dog have any littermates?	radio<br>1 Yes<br>0 No<br>2 Unknown
54	litinfo_yn<br>Show the field ONLY if: [petlit_yn] = 1	Are you able to give any information regarding your pet's littermates?	yesno<br>1 Yes<br>0 No
55	litcount<br>Show the field ONLY if: [litinfo_yn] = 1	How many littermates did your dog have?	text (number)
56	litinfo_names<br>Show the field ONLY if: [litinfo_yn] = 1	What is the name(s) of your dog's littermates (list all that are known)?	text
57	litinfo_owner<br>Show the field ONLY if: [litinfo_yn] = 1	What is the email address of the owner(s) of your dog's littermates (list all known)?	text
58	difflit_yn	Does your dog have any full siblings (shares same father and mother) from a DIFFERENT litter(s)?	radio<br>1 Yes<br>0 No<br>2 Unknown
59	difflitinfo_yn<br>Show the field ONLY if: [difflit_yn] = 1	Are you able to give any information pertaining to these full siblings?	yesno<br>1 Yes<br>0 No
60	fullsibcount<br>Show the field ONLY if: [difflitinfo_yn] = 1	Approximately how many other full siblings (from other litters) does your dog have?	dropdown<br>0 1-5<br>1 6-10<br>2 11-15<br>3 16-20<br>4 Greater than 20
61	fullsib_info<br>Show the field ONLY if: [difflitinfo_yn] = 1	What is the name(s) of your dog's full siblings (list any that are known)	text
62	fullsib_owner<br>Show the field ONLY if: [difflitinfo_yn] = 1	What is the email address of the owner(s) of your dog's full siblings (list if known):	text
63	offspring_yn	Does your dog have any offspring?	radio<br>1 Yes<br>0 No<br>2 Unknown
64	offspringcount<br>Show the field ONLY if: [offspring_yn] = 1	How many litters did your pet produce?	text (number)

65	offinfo_yn<br>Show the field ONLY if:<br>[offspring_yn] = 1	Are you able to provide any information pertaining to your pet's offspring?	yesno<br>1 Yes<br>0 No
66	offnames<br>Show the field ONLY if:<br>[offinfo_yn] = 1	What is the name(s) of your dog's offspring?	text
67	offowner<br>Show the field ONLY if:<br>[offinfo_yn] = 1	What is the email address of the owner(s) of your dog's offspring?	text
68	hh_yn	Section Header: *Your Pet's Housemates*<br>Are there any other dogs living in the household?	yesno<br>1 Yes<br>0 No
69	hh_count<br>Show the field ONLY if:<br>[hh_yn] = 1	How many other dogs live in the household?	dropdown<br>1 1<br>2 2<br>3 3<br>4 4<br>5 5<br>6 6<br>7 7<br>8 8<br>9 9<br>10 10<br>11 11<br>12 12<br>13 13<br>14 14<br>15 15<br>16 Greater than 15
70	hhdm_yn<br>Show the field ONLY if:<br>[hh_yn] = 1	Are any of these household dogs diabetic?	yesno<br>1 Yes<br>0 No
71	hhdm_count<br>Show the field ONLY if:<br>[hhdm_yn] = 1	How many household dogs are diabetic?	dropdown<br>1 1<br>2 2<br>3 3<br>4 4<br>5 5<br>6 Greater than 5
72	hhdmcall_name<br>Show the field ONLY if:<br>[hhdm_count] = '1' or [hhdm_count] = '2' or [hhdm_count] = '3' or [hhdm_count] = '4' or [hhdm_count] = '5' or [hhdm_c	What is the diabetic household dog's call name?	text

	ount] = '6'		
73	hhdmakc_yn<br>Show the field ONLY if:<br>[hhdm_count] = '1' or [hhdm_count] = '2' or [hhdm_count] = '3' or [hhdm_count] = '4' or [hhdm_count] = '5' or [hhdm_count] = '6'	Does THIS diabetic household dog have an AKC-registered name?	yesno, Required<br>1 Yes<br>0 No
74	hhdmakc_name<br>Show the field ONLY if:<br>[hhdm_count] = '1' or [hhdm_count] = '2' or [hhdm_count] = '3' or [hhdm_count] = '4' or [hhdm_count] = '5' or [hhdm_count] = '6' and ([hhdmakc_yn] = '1')	What is THIS diabetic household dog's AKC-registered name?	text
75	hhdm_breed<br>Show the field ONLY if:<br>[hhdm_count] = '1' or [hhdm_count] = '2' or [hhdm_count] = '3' or [hhdm_count] = '4' or [hhdm_count] = '5' or [hhdm_count] = '6'	What is THIS diabetic household dog's breed?	dropdown<br>1 Samoyed<br>2 Samoyed mix<br>3 Australian Terrier<br>4 Australian Terrier mix<br>5 Pug<br>6 Pug mix<br>7 American Eskimo<br>8 American Eskimo mix<br>9 Mixed breed<br>10 Other pure breed<br>11 German Shepherd<br>12 German Shepherd mix<br>13 Golden Retriever<br>14 Golden Retriever mix<br>15 American Pit Bull Terrier<br>16 American Pit Bull Terrier mix<br>17 Labrador Retriever<br>18 Labrador Retriever mix
76	hhdm_breedtype<br>Show the field ONLY if:<br>[hhdm_breed] = '10'	What is THIS diabetic household dog's breed?	text
77	hhdm_dob<br>Show the field ONLY if:<br>[hhdm_count] = '1' or [hhdm_count] = '2' or [hhdm_count] = '3' or [hhdm_count] = '4' or [hhdm_count] = '5' or [hhdm_count] = '6'	What is THIS diabetic household dog's date of birth?	text (date_mdy)
78	hhdm_age<br>Show the field ONLY if:<br>[hhdm_count] = '1' or [hhdm_count] = '2' or [hhdm_count] = '3' or [hhdm_count] = '4' or [	What is THIS diabetic household dog's age today?	dropdown<br>0 Less than 6 months old<br>1 6 months to 1 year old<br>2 1 year 1 month

	hhdm_count] = '5' or [hhdm_count] = '6'		3	1 year 2 months	
			4	1 year 3 months	
			5	1 year 4 months	
			6	1 year 5 months	
			7	1 year 6 months	
			8	1 year 7 months	
			9	1 year 8 months	
			10	1 year 9 months	
			11	1 year 10 months	
			12	1 year 11 months	
			13	2 years	
			14	2 years 1 month	
			15	2 years 2 months	
			16	2 years 3 months	
			17	2 years 4 months	
			18	2 years 5 months	
			19	2 years 6 months	
			20	2 years 7 months	
			21	2 years 8 months	
			22	2 years 9 months	
			23	2 years 10 months	
			24	2 years 11 months	
			25	3 years	
			26	3 years 1 month	
			27	3 years 2 months	
			28	3 years 3 months	
			29	3 years 4 months	
			30	3 years 5 months	
			31	3 years 6 months	
			32	3 years 7 months	
			33	3 years 8 months	
			34	3 years 9 months	
			35	3 years 10 months	
			36	3 years 11 months	
			37	4 years	
			38	4 years 1 month	
			39	4 years 2 months	
			40	4 years 3 months	
			41	4 years 4 months	
			42	4 years 5 months	
			43	4 years 6 months	
			44	4 years 7 months	
			45	4 years 8 months	
			46	4 years 9 months	

47	4 years 10 months
48	4 years 11 months
49	5 years
50	5 years 1 month
51	5 years 2 months
52	5 years 3 months
53	5 years 4 months
54	5 years 5 months
55	5 years 6 months
56	5 years 7 months
57	5 years 8 months
58	5 years 9 months
59	5 years 10 months
60	5 years 11 months
61	6 years
62	6 years 1 month
63	6 years 2 months
64	6 years 3 months
65	6 years 4 months
66	6 years 5 months
67	6 years 6 months
68	6 years 7 months
69	6 years 8 months
70	6 years 9 months
71	6 years 10 months
72	6 years 11 months
73	7 years
74	7 years 1 month
75	7 years 2 months
76	7 years 3 months
77	7 years 4 months
78	7 years 5 months
79	7 years 6 months
80	7 years 7 months
81	7 years 8 months
82	7 years 9 months
83	7 years 10 months
84	7 years 11 months
85	8 years
86	8 years 1 month
87	8 years 2 months
88	8 years 3 months
89	8 years 4 months

90	8 years 5 months
91	8 years 6 months
92	8 years 7 months
93	8 years 8 months
94	8 years 9 months
95	8 years 10 months
96	8 years 11 months
97	9 years
98	9 years 1 month
99	9 years 2 months
100	9 years 3 months
101	9 years 4 months
102	9 years 5 months
103	9 years 6 months
104	9 years 7 months
105	9 years 8 months
106	9 years 9 months
107	9 years 10 months
108	9 years 11 months
109	10 years
110	10 years 1 month
111	10 years 2 months
112	10 years 3 months
113	10 years 4 months
114	10 years 5 months
115	10 years 6 months
116	10 years 7 months
117	10 years 8 months
118	10 years 9 months
119	10 years 10 months
120	10 years 11 months
121	11 years
122	11 years 1 month
123	11 years 2 months
124	11 years 3 months
125	11 years 4 months
126	11 years 5 months
127	11 years 6 months
128	11 years 7 months
129	11 years 8 months
130	11 years 9 months
131	11 years 10 months
132	11 years 11 months

133	12 years
134	12 years 1 month
135	12 years 2 months
136	12 years 3 months
137	12 years 4 months
138	12 years 5 months
139	12 years 6 months
140	12 years 7 months
141	12 years 8 months
142	12 years 9 months
143	12 years 10 months
144	12 years 11 months
145	13 years
146	13 years 1 month
147	13 years 2 months
148	13 years 3 months
149	13 years 4 months
150	13 years 5 months
151	13 years 6 months
152	13 years 7 months
153	13 years 8 months
154	13 years 9 months
155	13 years 10 months
156	13 years 11 months
157	14 years
158	14 years 1 month
159	14 years 2 months
160	14 years 3 months
161	14 years 4 months
162	14 years 5 months
163	14 years 6 months
164	14 years 7 months
165	14 years 8 months
166	14 years 9 months
167	14 years 10 months
168	14 years 11 months
169	15 years
170	15 years 1 month
171	15 years 2 months
172	15 years 3 months
173	15 years 4 months
174	15 years 5 months
175	15 years 6 months
176	15 years 7 months

177	15 years 8 months
178	15 years 9 months
179	15 years 10 months
180	15 years 11 months
181	16 years
182	16 years 1 month
183	16 years 2 month
184	16 years 3 months
185	16 years 4 months
186	16 years 5 months
187	16 years 6 months
188	16 years 7 months
189	16 years 8 months
190	16 years 9 months
191	16 years 10 months
192	16 years 11 months
193	17 years
194	17 years 1 month
195	17 years 2 months
196	17 years 3 months
197	17 years 4 months
198	17 years 5 months
199	17 years 6 months
200	17 years 7 months
201	17 years 8 months
202	17 years 9 months
203	17 years 10 months
204	17 years 11 months
205	18 years
206	18 years 1 month
207	18 years 2 months
208	18 years 3 months
209	18 years 4 months
210	18 years 5 months
211	18 years 6 months
212	18 years 7 months
213	18 years 8 months
214	18 years 9 months
215	18 years 10 months
216	18 years 11 months
217	19 years
218	Over 19 years old

79	hhdm_sex<br>Show the field ONLY if:<br>[hhdm_count] = '1' or [hhdm_count] = '2' or [hhdm_count] = '3' or [hhdm_count] = '4' or [hhdm_count] = '5' or [hhdm_count] = '6'	What is THIS diabetic household dog's sex?	dropdown<br>1 Intact male<br>2 Intact female<br>3 Neutered male<br>4 Spayed female
80	hhdm_weight<br>Show the field ONLY if:<br>[hhdm_count] = '1' or [hhdm_count] = '2' or [hhdm_count] = '3' or [hhdm_count] = '4' or [hhdm_count] = '5' or [hhdm_count] = '6'	What is THIS diabetic household dog's approximate weight in pounds?	text (number)
81	hhdm_diet<br>Show the field ONLY if:<br>[hhdm_count] = '1' or [hhdm_count] = '2' or [hhdm_count] = '3' or [hhdm_count] = '4' or [hhdm_count] = '5' or [hhdm_count] = '6'	What diet (brand and type) are you feeding THIS diabetic household dog?	text
82	hhdm_ins<br>Show the field ONLY if:<br>[hhdm_count] = '1' or [hhdm_count] = '2' or [hhdm_count] = '3' or [hhdm_count] = '4' or [hhdm_count] = '5' or [hhdm_count] = '6'	Is THIS diabetic household dog receiving insulin?	yesno<br>1 Yes<br>0 No
83	hhdm_instype<br>Show the field ONLY if:<br>[hhdm_ins] = 1	Which type of insulin is THIS diabetic household dog receiving?	dropdown<br>0 NPH / Humulin-N / Novolin-N<br>1 Lantus / Glargine<br>2 Vetsulin<br>3 PZI<br>4 Levemir / Detemir<br>5 Other
84	hhdm_instypeother<br>Show the field ONLY if:<br>[hhdm_instype] = '5'	What is the name of insulin which THIS diabetic household dog is receiving?	text
85	hhdm_insfreq<br>Show the field ONLY if:<br>[hhdm_ins] = 1	How many times a day does THIS household dog receive insulin?	dropdown<br>0 Once daily<br>1 Twice daily<br>2 Three times daily
86	hhdm_insamnt<br>Show the field ONLY if:<br>[hhdm_ins] = 1	How many units of insulin does THIS household dog receive with each injection?	dropdown<br>1 1 units<br>2 2 units<br>3 3 units<br>4 4 units<br>5 5 units<br>6 6 units<br>7 7 units

			8 8 units<br>9 9 units<br>10 10 units<br>11 11 units<br>12 12 units<br>13 13 units<br>14 14 units<br>15 15 units<br>16 16 units<br>17 17 units<br>18 18 units<br>19 19 units<br>20 20 units<br>21 21 units<br>22 22 units<br>23 23 units<br>24 24 units<br>25 25 units<br>26 26 units<br>27 27 units<br>28 28 units<br>29 29 units<br>30 30 units<br>31 31 units<br>32 32 units<br>33 33 units<br>34 34 units<br>35 35 units or more
87	hhdm_dx<br>Show the field ONLY if:<br>[hhdm_ins] = 1	When was THIS diabetic household dog diagnosed with diabetes (approximate if unknown)?	text (date_mdy)
88	hhdmcall_name2<br>Show the field ONLY if:<br>[hhdm_count] = '2' or [hhdm_count] = '3' or [hhdm_count] = '4' or [hhdm_count] = '5' or [hhdm_count] = '6'	What is the second diabetic household dog's call name?	text
89	hhdmakc_name2<br>Show the field ONLY if:<br>[hhdm_count] = '2' or [hhdm_count] = '3' or [hhdm_count] = '4' or [hhdm_count] = '5' or [hhdm_count] = '6'	What is THIS diabetic household dog's AKC-registered name?	text
90	hhdm_breed2<br>Show the field ONLY if:<br>[hhdm_count] = '2' or [hhdm_count] = '3' or [hhdm_count]	What is THIS diabetic household dog's breed?	dropdown<br>1 Samoyed<br>2 Samoyed mix

	= '4' or [hhdm_count] = '5' or [hhdm_count] = '6'		3 Australian Terrier<br>4 Australian Terrier mix<br>5 Pug<br>6 Pug mix<br>7 American Eskimo<br>8 American Eskimo mix<br>9 Mixed breed<br>10 Other pure breed<br>11 German Shepherd<br>12 German Shepherd mix<br>13 Golden Retriever<br>14 Golden Retriever mix<br>15 American Pit Bull Terrier<br>16 American Pit Bull Terrier mix<br>17 Labrador Retriever<br>18 Labrador Retriever mix
91	hhdm_dob2<br>Show the field ONLY if:<br>[hhdm_count] = '2' or [hhdm_ count] = '3' or [hhdm_count] = '4' or [hhdm_count] = '5' or [hhdm_count] = '6'	What is THIS diabetic household dog's date of birth?	text (date_mdy)
92	hhdm_age2<br>Show the field ONLY if:<br>[hhdm_count] = '2' or [hhdm_ count] = '3' or [hhdm_count] = '4' or [hhdm_count] = '5' or [hhdm_count] = '6'	What is THIS diabetic household dog's age today?	dropdown<br>0 Less than 6 months old<br>1 6 months to 1 year old<br>2 1 year 1 month<br>3 1 year 2 months<br>4 1 year 3 months<br>5 1 year 4 months<br>6 1 year 5 months<br>7 1 year 6 months<br>8 1 year 7 months<br>9 1 year 8 months<br>10 1 year 9 months<br>11 1 year 10 months<br>12 1 year 11 months<br>13 2 years<br>14 2 years 1 month<br>15 2 years 2 months<br>16 2 years 3 months<br>17 2 years 4 months<br>18 2 years 5 months<br>19 2 years 6 months<br>20 2 years 7 months<br>21 2 years 8 months

22	2 years 9 months
23	2 years 10 months
24	2 years 11 months
25	3 years
26	3 years 1 month
27	3 years 2 months
28	3 years 3 months
29	3 years 4 months
30	3 years 5 months
31	3 years 6 months
32	3 years 7 months
33	3 years 8 months
34	3 years 9 months
35	3 years 10 months
36	3 years 11 months
37	4 years
38	4 years 1 month
39	4 years 2 months
40	4 years 3 months
41	4 years 4 months
42	4 years 5 months
43	4 years 6 months
44	4 years 7 months
45	4 years 8 months
46	4 years 9 months
47	4 years 10 months
48	4 years 11 months
49	5 years
50	5 years 1 month
51	5 years 2 months
52	5 years 3 months
53	5 years 4 months
54	5 years 5 months
55	5 years 6 months
56	5 years 7 months
57	5 years 8 months
58	5 years 9 months
59	5 years 10 months
60	5 years 11 months
61	6 years
62	6 years 1 month
63	6 years 2 months
64	6 years 3 months
65	6 years 4 months

66	6 years 5 months
67	6 years 6 months
68	6 years 7 months
69	6 years 8 months
70	6 years 9 months
71	6 years 10 months
72	6 years 11 months
73	7 years
74	7 years 1 month
75	7 years 2 months
76	7 years 3 months
77	7 years 4 months
78	7 years 5 months
79	7 years 6 months
80	7 years 7 months
81	7 years 8 months
82	7 years 9 months
83	7 years 10 months
84	7 years 11 months
85	8 years
86	8 years 1 month
87	8 years 2 months
88	8 years 3 months
89	8 years 4 months
90	8 years 5 months
91	8 years 6 months
92	8 years 7 months
93	8 years 8 months
94	8 years 9 months
95	8 years 10 months
96	8 years 11 months
97	9 years
98	9 years 1 month
99	9 years 2 months
100	9 years 3 months
101	9 years 4 months
102	9 years 5 months
103	9 years 6 months
104	9 years 7 months
105	9 years 8 months
106	9 years 9 months
107	9 years 10 months
108	9 years 11 months

109	10 years
110	10 years 1 month
111	10 years 2 months
112	10 years 3 months
113	10 years 4 months
114	10 years 5 months
115	10 years 6 months
116	10 years 7 months
117	10 years 8 months
118	10 years 9 months
119	10 years 10 months
120	10 years 11 months
121	11 years
122	11 years 1 month
123	11 years 2 months
124	11 years 3 months
125	11 years 4 months
126	11 years 5 months
127	11 years 6 months
128	11 years 7 months
129	11 years 8 months
130	11 years 9 months
131	11 years 10 months
132	11 years 11 months
133	12 years
134	12 years 1 month
135	12 years 2 months
136	12 years 3 months
137	12 years 4 months
138	12 years 5 months
139	12 years 6 months
140	12 years 7 months
141	12 years 8 months
142	12 years 9 months
143	12 years 10 months
144	12 years 11 months
145	13 years
146	13 years 1 month
147	13 years 2 months
148	13 years 3 months
149	13 years 4 months
150	13 years 5 months
151	13 years 6 months

			152	13 years 7 months	
			153	13 years 8 months	
			154	13 years 9 months	
			155	13 years 10 months	
			156	13 years 11 months	
			157	14 years	
			158	14 years 1 month	
			159	14 years 2 months	
			160	14 years 3 months	
			161	14 years 4 months	
			162	14 years 5 months	
			163	14 years 6 months	
			164	14 years 7 months	
			165	14 years 8 months	
			166	14 years 9 months	
			167	14 years 10 months	
			168	14 years 11 months	
			169	15 years	
			170	15 years 1 month	
			171	15 years 2 months	
			172	15 years 3 months	
			173	15 years 4 months	
			174	15 years 5 months	
			175	15 years 6 months	
			176	15 years 7 months	
			177	15 years 8 months	
			178	15 years 9 months	
			179	15 years 10 months	
			180	15 years 11 months	
			181	16 years	
			182	16 years 1 month	
			183	16 years 2 month	
			184	16 years 3 months	
			185	16 years 4 months	
			186	16 years 5 months	
			187	16 years 6 months	
			188	16 years 7 months	
			189	16 years 8 months	
			190	16 years 9 months	
			191	16 years 10 months	
			192	16 years 11 months	
			193	17 years	
			194	17 years 1 month	

			195 17 years 2 months<br>196 17 years 3 months<br>197 17 years 4 months<br>198 17 years 5 months<br>199 17 years 6 months<br>200 17 years 7 months<br>201 17 years 8 months<br>202 17 years 9 months<br>203 17 years 10 months<br>204 17 years 11 months<br>205 18 years<br>206 18 years 1 month<br>207 18 years 2 months<br>208 18 years 3 months<br>209 18 years 4 months<br>210 18 years 5 months<br>211 18 years 6 months<br>212 18 years 7 months<br>213 18 years 8 months<br>214 18 years 9 months<br>215 18 years 10 months<br>216 18 years 11 months<br>217 19 years<br>218 Over 19 years old
93	hhdm_sex2<br>Show the field ONLY if:<br>[hhdm_count] = '2' or [hhdm_count] = '3' or [hhdm_count] = '4' or [hhdm_count] = '5' or [hhdm_count] = '6'	What is THIS diabetic household dog's sex?	dropdown<br>1 Intact male<br>2 Intact female<br>3 Neutered male<br>4 Spayed female
94	hhdm_weight2<br>Show the field ONLY if:<br>[hhdm_count] = '2' or [hhdm_count] = '3' or [hhdm_count] = '4' or [hhdm_count] = '5' or [hhdm_count] = '6'	What is THIS diabetic household dog's approximate weight in pounds?	text (integer)
95	hhdm_diet2<br>Show the field ONLY if:<br>[hhdm_count] = '2' or [hhdm_count] = '3' or [hhdm_count] = '4' or [hhdm_count] = '5' or [hhdm_count] = '6'	What diet (brand and type) are you feeding THIS diabetic household dog?	text
96	hhdm_ins2<br>Show the field ONLY if:<br>[hhdm_count] = '2' or [hhdm_count] = '3' or [hhdm_count] = '4' or [hhdm_count] = '5' or [hhdm_count] = '6'	Is THIS diabetic household dog receiving insulin?	yesno<br>1 Yes<br>0 No

97	hhdm_instype2<br>Show the field ONLY if:<br>[hhdm_ins2] = '1'	Which type of insulin is THIS diabetic household dog receiving?	dropdown<br>0 NPH / Humulin-N / Novolin-N<br>1 Lantus / Glargine<br>2 Vetsulin<br>3 PZI<br>4 Levemir / Detemir<br>5 Other
98	hhdm_instypeother2<br>Show the field ONLY if:<br>[hhdm_instype2] = '5'	What is the name of insulin which THIS diabetic household dog is receiving?	text
99	hhdm_insfreq2<br>Show the field ONLY if:<br>[hhdm_ins2] = 1	How many times a day does THIS household dog receive insulin?	dropdown<br>0 Once daily<br>1 Twice daily<br>2 Three times daily
100	hhdm_insamnt2<br>Show the field ONLY if:<br>[hhdm_ins2] = 1	How many units of insulin does THIS household dog receive with each injection?	dropdown<br>1 1 units<br>2 2 units<br>3 3 units<br>4 4 units<br>5 5 units<br>6 6 units<br>7 7 units<br>8 8 units<br>9 9 units<br>10 10 units<br>11 11 units<br>12 12 units<br>13 13 units<br>14 14 units<br>15 15 units<br>16 16 units<br>17 17 units<br>18 18 units<br>19 19 units<br>20 20 units<br>21 21 units<br>22 22 units<br>23 23 units<br>24 24 units<br>25 25 units<br>26 26 units<br>27 27 units<br>28 28 units<br>29 29 units

			30 30 units<br>31 31 units<br>32 32 units<br>33 33 units<br>34 34 units<br>35 35 units or more
101	hhdm_dx2<br>Show the field ONLY if:<br>[hhdm_ins2] = 1	When was THIS diabetic household dog diagnosed with diabetes (approximate if unknown)?	text (date_mdy)
102	hhdmcall_name3<br>Show the field ONLY if:<br>[hhdm_count] = '3' or [hhdm_count] = '4' or [hhdm_count] = '5' or [hhdm_count] = '6'	What is the third diabetic household dog's call name?	text
103	hhdmakc_name3<br>Show the field ONLY if:<br>[hhdm_count] = '3' or [hhdm_count] = '4' or [hhdm_count] = '5' or [hhdm_count] = '6'	What is THIS diabetic household dog's AKC-registered name?	text
104	hhdm_breed3<br>Show the field ONLY if:<br>[hhdm_count] = '3' or [hhdm_count] = '4' or [hhdm_count] = '5' or [hhdm_count] = '6'	What is THIS diabetic household dog's breed?	dropdown<br>1 Samoyed<br>2 Samoyed mix<br>3 Australian Terrier<br>4 Australian Terrier mix<br>5 Pug<br>6 Pug mix<br>7 American Eskimo<br>8 American Eskimo mix<br>9 Mixed breed<br>10 Other pure breed<br>11 German Shepherd<br>12 German Shepherd mix<br>13 Golden Retriever<br>14 Golden Retriever mix<br>15 American Pit Bull Terrier<br>16 American Pit Bull Terrier mix<br>17 Labrador Retriever<br>18 Labrador Retriever mix
105	hhdm_dob3<br>Show the field ONLY if:<br>[hhdm_count] = '3' or [hhdm_count] = '4' or [hhdm_count] = '5' or [hhdm_count] = '6'	What is THIS diabetic household dog's date of birth?	text (date_mdy)
106	hhdm_age3<br>Show the field ONLY if:<br>[hhdm_count] = '3' or [hhdm_count] = '4' or [hhdm_count]	What is THIS diabetic household dog's age today?	dropdown<br>0 Less than 6 months old<br>1 6 months to 1 year old

	= '5' or [hhdm_count] = '6'		2	1 year 1 month	
			3	1 year 2 months	
			4	1 year 3 months	
			5	1 year 4 months	
			6	1 year 5 months	
			7	1 year 6 months	
			8	1 year 7 months	
			9	1 year 8 months	
			10	1 year 9 months	
			11	1 year 10 months	
			12	1 year 11 months	
			13	2 years	
			14	2 years 1 month	
			15	2 years 2 months	
			16	2 years 3 months	
			17	2 years 4 months	
			18	2 years 5 months	
			19	2 years 6 months	
			20	2 years 7 months	
			21	2 years 8 months	
			22	2 years 9 months	
			23	2 years 10 months	
			24	2 years 11 months	
			25	3 years	
			26	3 years 1 month	
			27	3 years 2 months	
			28	3 years 3 months	
			29	3 years 4 months	
			30	3 years 5 months	
			31	3 years 6 months	
			32	3 years 7 months	
			33	3 years 8 months	
			34	3 years 9 months	
			35	3 years 10 months	
			36	3 years 11 months	
			37	4 years	
			38	4 years 1 month	
			39	4 years 2 months	
			40	4 years 3 months	
			41	4 years 4 months	
			42	4 years 5 months	
			43	4 years 6 months	
			44	4 years 7 months	
			45	4 years 8 months	

46	4 years 9 months
47	4 years 10 months
48	4 years 11 months
49	5 years
50	5 years 1 month
51	5 years 2 months
52	5 years 3 months
53	5 years 4 months
54	5 years 5 months
55	5 years 6 months
56	5 years 7 months
57	5 years 8 months
58	5 years 9 months
59	5 years 10 months
60	5 years 11 months
61	6 years
62	6 years 1 month
63	6 years 2 months
64	6 years 3 months
65	6 years 4 months
66	6 years 5 months
67	6 years 6 months
68	6 years 7 months
69	6 years 8 months
70	6 years 9 months
71	6 years 10 months
72	6 years 11 months
73	7 years
74	7 years 1 month
75	7 years 2 months
76	7 years 3 months
77	7 years 4 months
78	7 years 5 months
79	7 years 6 months
80	7 years 7 months
81	7 years 8 months
82	7 years 9 months
83	7 years 10 months
84	7 years 11 months
85	8 years
86	8 years 1 month
87	8 years 2 months
88	8 years 3 months

89	8 years 4 months
90	8 years 5 months
91	8 years 6 months
92	8 years 7 months
93	8 years 8 months
94	8 years 9 months
95	8 years 10 months
96	8 years 11 months
97	9 years
98	9 years 1 month
99	9 years 2 months
100	9 years 3 months
101	9 years 4 months
102	9 years 5 months
103	9 years 6 months
104	9 years 7 months
105	9 years 8 months
106	9 years 9 months
107	9 years 10 months
108	9 years 11 months
109	10 years
110	10 years 1 month
111	10 years 2 months
112	10 years 3 months
113	10 years 4 months
114	10 years 5 months
115	10 years 6 months
116	10 years 7 months
117	10 years 8 months
118	10 years 9 months
119	10 years 10 months
120	10 years 11 months
121	11 years
122	11 years 1 month
123	11 years 2 months
124	11 years 3 months
125	11 years 4 months
126	11 years 5 months
127	11 years 6 months
128	11 years 7 months
129	11 years 8 months
130	11 years 9 months
131	11 years 10 months
132	11 years 11 months

133	12 years
134	12 years 1 month
135	12 years 2 months
136	12 years 3 months
137	12 years 4 months
138	12 years 5 months
139	12 years 6 months
140	12 years 7 months
141	12 years 8 months
142	12 years 9 months
143	12 years 10 months
144	12 years 11 months
145	13 years
146	13 years 1 month
147	13 years 2 months
148	13 years 3 months
149	13 years 4 months
150	13 years 5 months
151	13 years 6 months
152	13 years 7 months
153	13 years 8 months
154	13 years 9 months
155	13 years 10 months
156	13 years 11 months
157	14 years
158	14 years 1 month
159	14 years 2 months
160	14 years 3 months
161	14 years 4 months
162	14 years 5 months
163	14 years 6 months
164	14 years 7 months
165	14 years 8 months
166	14 years 9 months
167	14 years 10 months
168	14 years 11 months
169	15 years
170	15 years 1 month
171	15 years 2 months
172	15 years 3 months
173	15 years 4 months
174	15 years 5 months
175	15 years 6 months

176	15 years 7 months
177	15 years 8 months
178	15 years 9 months
179	15 years 10 months
180	15 years 11 months
181	16 years
182	16 years 1 month
183	16 years 2 month
184	16 years 3 months
185	16 years 4 months
186	16 years 5 months
187	16 years 6 months
188	16 years 7 months
189	16 years 8 months
190	16 years 9 months
191	16 years 10 months
192	16 years 11 months
193	17 years
194	17 years 1 month
195	17 years 2 months
196	17 years 3 months
197	17 years 4 months
198	17 years 5 months
199	17 years 6 months
200	17 years 7 months
201	17 years 8 months
202	17 years 9 months
203	17 years 10 months
204	17 years 11 months
205	18 years
206	18 years 1 month
207	18 years 2 months
208	18 years 3 months
209	18 years 4 months
210	18 years 5 months
211	18 years 6 months
212	18 years 7 months
213	18 years 8 months
214	18 years 9 months
215	18 years 10 months
216	18 years 11 months
217	19 years
218	Over 19 years old

107	hhdm_sex3<br>Show the field ONLY if:<br>[hhdm_count] = '3' or [hhdm_count] = '4' or [hhdm_count] = '5' or [hhdm_count] = '6'	What is THIS diabetic household dog's sex?	dropdown<br>1 Intact male<br>2 Intact female<br>3 Neutered male<br>4 Spayed female
108	hhdm_weight3<br>Show the field ONLY if:<br>[hhdm_count] = '3' or [hhdm_count] = '4' or [hhdm_count] = '5' or [hhdm_count] = '6'	What is THIS diabetic household dog's approximate weight in pounds?	text (number)
109	hhdm_diet3<br>Show the field ONLY if:<br>[hhdm_count] = '3' or [hhdm_count] = '4' or [hhdm_count] = '5' or [hhdm_count] = '6'	What diet (brand and type) are you feeding THIS diabetic household dog?	text
110	hhdm_ins3<br>Show the field ONLY if:<br>[hhdm_count] = '3' or [hhdm_count] = '4' or [hhdm_count] = '5' or [hhdm_count] = '6'	Is THIS diabetic household dog receiving insulin?	yesno<br>1 Yes<br>0 No
111	hhdm_instype3<br>Show the field ONLY if:<br>[hhdm_ins3] = '1'	Which type of insulin is THIS diabetic household dog receiving?	dropdown<br>0 NPH / Humulin-N / Novolin-N<br>1 Lantus / Glargine<br>2 Vetsulin<br>3 PZI<br>4 Levemir / Detemir<br>5 Other
112	hhdm_instypeother3<br>Show the field ONLY if:<br>[hhdm_instype3] = '5'	What is the name of insulin which THIS diabetic household dog is receiving?	text
113	hhdm_insfreq3<br>Show the field ONLY if:<br>[hhdm_ins3] = 1	How many times a day does THIS household dog receive insulin?	dropdown<br>0 Once daily<br>1 Twice daily<br>2 Three times daily
114	hhdm_insamnt3<br>Show the field ONLY if:<br>[hhdm_ins3] = 1	How many units of insulin does THIS household dog receive with each injection?	dropdown<br>1 1 units<br>2 2 units<br>3 3 units<br>4 4 units<br>5 5 units<br>6 6 units<br>7 7 units<br>8 8 units<br>9 9 units<br>10 10 units<br>11 11 units<br>12 12 units

			13 13 units<br>14 14 units<br>15 15 units<br>16 16 units<br>17 17 units<br>18 18 units<br>19 19 units<br>20 20 units<br>21 21 units<br>22 22 units<br>23 23 units<br>24 24 units<br>25 25 units<br>26 26 units<br>27 27 units<br>28 28 units<br>29 29 units<br>30 30 units<br>31 31 units<br>32 32 units<br>33 33 units<br>34 34 units<br>35 35 units or more
115	hhdm_dx3<br>Show the field ONLY if:<br>[hhdm_ins3] = 1	When was THIS diabetic household dog diagnosed with diabetes (approximate if unknown)?	text (date_mdy)
116	deceased_dm	Section Header: *Miscellaneous information*<br>Are you aware of a deceased diabetic dog that was directly related to the dog in this survey?	yesno<br>1 Yes<br>0 No
117	deceased_info<br>Show the field ONLY if:<br>[deceased_dm] = 1	Would you like to share information regarding this deceased diabetic dog?	yesno<br>1 Yes<br>0 No
118	deceased_callname<br>Show the field ONLY if:<br>[deceased_info] = 1	What was this deceased diabetic dog's call name?	text
119	deceased_akcyn<br>Show the field ONLY if:<br>[deceased_info] = 1	Did this deceased diabetic dog have an AKC-registered name?	yesno<br>1 Yes<br>0 No
120	deceased_akcnames<br>Show the field ONLY if:<br>[deceased_akcyn] = 1	What was this deceased diabetic dog's AKC-registered name?	text
121	deceased_breed<br>Show the field ONLY if:<br>[deceased_info] = 1	What was this deceased diabetic dog's breed?	dropdown<br>1 Samoyed<br>2 Samoyed mix

			3 Australian Terrier<br>4 Australian Terrier mix<br>5 Pug<br>6 Pug mix<br>7 American Eskimo<br>8 American Eskimo mix<br>9 Mixed breed<br>10 Other pure breed<br>11 German Shepherd<br>12 German Shepherd mix<br>13 Golden Retriever<br>14 Golden Retriever mix<br>15 American Pit Bull Terrier<br>16 American Pit Bull Terrier mix<br>17 Labrador Retriever<br>18 Labrador Retriever mix
122	deceased_breed2<br>Show the field ONLY if:<br>[deceased_breed] = 10	What was this deceased diabetic dog's breed?	text
123	deceased_sex<br>Show the field ONLY if:<br>[deceased_info] = 1	What was this deceased diabetic dog's sex?	dropdown<br>1 Intact male<br>2 Intact female<br>3 Neutered male<br>4 Spayed female
124	deceased_dob<br>Show the field ONLY if:<br>[deceased_info] = 1	What was this deceased diabetic dog's date of birth (approximate if unknown)?	text (date_mdy)
125	deceased_dod<br>Show the field ONLY if:<br>[deceased_info] = 1	When did this diabetic dog become deceased (approximate if unknown)?	text (date_mdy)
126	deceased_dmdx<br>Show the field ONLY if:<br>[deceased_info] = 1	When was this deceased diabetic dog diagnosed with diabetes (approximate if unknown)?	text (date_mdy)
127	deceased_weight<br>Show the field ONLY if:<br>[deceased_info] = 1	Approximately how much did this deceased diabetic dog weigh in pounds (at adulthood)?	text (number)
128	deceased_insulin<br>Show the field ONLY if:<br>[deceased_info] = 1	Which type of insulin did this deceased diabetic dog receive?	dropdown<br>0 NPH / Humulin-N / Novolin-N<br>1 Lantus / Glargine<br>2 Vetsulin<br>3 PZI<br>4 Levemir / Detemir<br>5 Other<br>6 Unknown

129	deceased_insulinother<br>Show the field ONLY if:<br>[deceased_insulin] = 5	What is the name of the insulin that this deceased diabetic dog received?	text
130	deceased_insfreq<br>Show the field ONLY if:<br>[deceased_info] = 1	How many times a day did this deceased diabetic dog receive insulin?	dropdown<br>0 Once daily<br>1 Twice daily<br>2 Three times daily<br>3 Unknown
131	deceased_insamount<br>Show the field ONLY if:<br>[deceased_info] = 1	How many units of insulin did this deceased diabetic dog receive with each injection?	dropdown<br>1 1 units<br>2 2 units<br>3 3 units<br>4 4 units<br>5 5 units<br>6 6 units<br>7 7 units<br>8 8 units<br>9 9 units<br>10 10 units<br>11 11 units<br>12 12 units<br>13 13 units<br>14 14 units<br>15 15 units<br>16 16 units<br>17 17 units<br>18 18 units<br>19 19 units<br>20 20 units<br>21 21 units<br>22 22 units<br>23 23 units<br>24 24 units<br>25 25 units<br>26 26 units<br>27 27 units<br>28 28 units<br>29 29 units<br>30 30 units<br>31 31 units<br>32 32 units<br>33 33 units<br>34 34 units<br>35 35 units or more

			36 Unknown
132	comments	Your input is greatly appreciated. Please provide us with any comments or feedback (optional):	notes
133	diabetes_mellitus_survey_complete	Section Header: *Form Status*<br>Complete?	dropdown<br>0 Incomplete<br>1 Unverified<br>2 Complete
