# Supplementary material for: Seasonality and geography of diabetes mellitus in United States of America dogs
Source: PLoS One. 2022 Aug 5;17(8):e0272297. doi: 10.1371/journal.pone.0272297 (PMC9355170; doi:10.1371/journal.pone.0272297)
Supplement: S4 Appendix — (PDF) [file pone.0272297.s004.pdf]

own_add	surv_date	breed_type	mixedbreed	dob	pet_age	neut_stat
20	6/15/17	102	Kerry blue terrier	6/7/17	73	2
		1		11/15/04	148	3
46	2/26/18	1		1/1/18	98	1
38	8/10/17	36	english setter	7/25/17	97	2
14	7/5/17	23	weimaraner	8/24/16	1	1
47	4/27/17	5		5/12/04		3
		1		8/8/08	97	3
34	2/26/18	61	brittany	4/25/16	23	4
8	3/30/18	93	cardigan Welsh Cor	6/12/16	4	4
4	1/26/18	17		9/29/12	53	3
38	4/17/17	91	jack russell Terrier	1-Mar-16	2	4
43	9/14/17	16		4/15/12	55	4
5	8/14/17	103	Tibetan Spaniel	2/4/07	115	4
38	6/6/17	67	west highland whit	2/3/17	113	4
9	4/10/18	15		10/30/17	0	1
38	12/10/19	1		5/1/19	1	1
38	5/4/17	17		5/4/17	0	1
19	9/20/17	56	border collie	7/21/16	3	4
40	9/8/18	1		1/15/09	106	3
4	8/18/17	82	miniature schnauze	3/5/17	0	1
34	11/18/18	1		11/1/15	13	4
4	3/11/19	49	Old english sheepd	10/6/13	54	3
8	6/28/18	68	maltese	7/28/08	108	3
16	9/6/17	77	bichon Frise	6/8/15	16	3
43	6/25/18	1		4/15/17	3	3
38	7/12/17	15		1/16/16	8	4
	7/28/17	29	chow chow	3/28/15	18	1
42	3/11/18	97	miniature pinscher	3/1/14	37	4
33	10/30/19	8	french bulldog	5/1/18	6	4
13	2/28/18	31	akita	6/3/13	45	1
46	7/12/17	1		12/7/11	68	4
4	2/10/18	5		5/2/11	82	4
3	9/5/17	44	Cane corso	6/1/11	64	4
5	8/14/17	97	Miniature Pinscher	12/5/07	106	3
	3/13/18	16		3/21/10	84	3
38	5/10/17	15		3/21/15	15	4
43	1/4/18	61	brittany	4/1/06	130	4
46	9/5/18	56	border collie	7/16/16	15	3
15	8/12/17	75	Shih tzu	5/12/07	112	4
15	7/12/17	75	Shih tzu	1/1/06	116	4
33	5/20/17	82	Mini Schnauzer	4/1/08	99	3
48	1/26/18	27	doberman Pincher	6/10/14	32	4
30	8/13/18	71	cavalier King Charle	12/13/15	139	4
38	4/26/17	5		5/7/01		3

		15		7/15/14		3
48	2/27/18	26	Vizsla	1/21/12	62	3
5	5/22/17	84	schnauzer	3/26/14	38	4
34	9/7/17	1		6/1/11	64	3
4	1/8/18	80	australian shepherd	5/22/06	129	3
6	8/25/17	5		9/11/01	177	4
9	3/11/19	82	miniature schnauze	3/28/15	36	3
50	7/27/17	6		9/13/13	35	1
49	4/9/18	1		12/14/05	133	3
24	3/24/18	17		5/15/09	97	3
8		76	Lhaso apso	10/5/15	133	3
	7/25/17	17		1/31/08	91	3
18	9/1/17	94	dachshund	9/1/05	133	3
		17		4/28/11	62	3
37	10/28/17	5		11/27/12	48	3
46	6/16/18	3	australian terrier	4/1/12	63	3
3	8/3/17	1		6/19/13	39	3
0	1/24/18	97	miniature pinscher	12/11/13	50	3
	8/3/17	6		8/20/13	49	3
33	2/7/18	82	miniature schnauze	4/1/10	85	3
21	8/14/17	78	pomeranian	1/1/07	116	3
4	11/8/17	1		3/23/14	32	4
40	3/7/18	97	miniature pinscher	6/1/10	82	3
49		1		3/20/09	89	4
16	5/22/17	1		9/11/12	49	4
16	5/22/17	1		9/11/12	49	4
		97	Miniature Pinscher	10/6/07	120	4
14	3/12/19	83	Schnauzer	1/28/12	75	4
18	6/5/17	2	Polish Lowland She	3/5/13	40	2
32		1		6/12/13	45	4
38	7/27/17	2	Pharaoh Hound	8/28/07	120	3
30	2/7/18	2	Belgian Sheepdog	5/8/05	142	4
34	8/1/17	5		3/1/08		
19	9/27/17	78	pomeranian	7/3/13	51	3
13	2/5/18	1		5/30/10	81	4
14	1/26/18	1		8/29/13	42	3
14	7/6/17	16		3/27/13	40	1
40	2/27/18	78	pomeranian	8/21/12	43	4
25	7/28/17	5		7/4/04	145	4
13	2/5/18	1		4/1/13	46	3
4	1/22/18	48	english mastiff	8/1/11	66	1
30	5/27/18	2		7/4/13	48	3
48	6/5/17	97	miniature pinscher	3/18/09	88	3
3	1/24/18	97	miniature pinscher	2/16/04	149	4
30	12/18/18	1		11/15/14	38	3

29	7/1/17	34	Siberian husky	6/12/09	85	4
34	5/22/17	1		10/19/12	68	3
18	5/9/17	63	toy poodle	5/5/08	97	3
3	6/6/17	80	Australian Shepher	4/25/11	62	4
18	9/2/17	82	miniature schnauze	10/6/10	73	4
47	6/8/19	92	pembroke welsh co	4/1/08	111	3
31	8/15/19	1		5/1/11	88	4
38	4/18/17	27	doberman Pinscher	4/19/12	48	4
38	6/5/18	68	maltese	6/5/12	49	4
33	5/22/17	17		12/1/10	78	3
30	10/19/17	64	miniature poodle	5/15/13	150	3
42	8/14/17	17		5/10/05	136	4
7	3/12/19	1		8/31/07	128	3
23	3/19/18	92	pembroke Welsh C	1/12/07	124	3
31	11/11/18	1		9/11/10	87	1
34	5/22/17	1		12/12/12	42	3
20	11/1/19	1		5/31/11	90	4
18	10/1/18	1		6/21/12	64	3
38	11/6/18	18	Chesapeake bay ret	3/18/14	45	1
	7/30/17	63	toy poodle	3/20/09	89	4
38	12/4/18	6		3/27/14	46	3
22	6/19/17	84	schnauzer	9/20/06	130	4
11	3/10/18	32	Great dane	5/23/13	47	4
22	5/22/17	66	beagle	7/28/12	93	3
0	1/21/18	31	akita	7/4/12	55	4
43	8/3/17	97	miniature pinscher	2/12/11	66	4
6	4/26/17	5		9/11/11	56	3
4	1/8/18	58	english cocker Spar	7/1/12	67	4
18	3/11/19	1		2/22/11	74	3
35	4/28/17	1		4/28/12	49	3
8	2/5/18	6		10/6/12	49	3
30	10/11/18	92	pembroke Corgi	12/9/13	47	4
4	7/11/18	27	doberman (A Blue)	9/1/13	49	4
48	2/27/18	66	beagle	2/6/06	133	4
32	5/22/17	1		4/15/06	121	3
	7/27/17	1		7/24/09	85	4
		97	Miniature Pinscher	8/1/04	145	4
33	9/12/17	74	havanese	6/7/12	52	4
14	12/31/17	2	Llewellin Setter	1/1/10	84	4
38	7/31/17	39	Saint bernard	7/1/11	91	4
4	7/24/17	82	miniature schnauze	3/1/10	79	4
6	2/12/18	1		1/10/07	122	4
38	5/22/17	97	miniature pinscher	3/1/08	100	2
20	1/26/18	1		12/5/10	86	4
6	7/29/17	5		12/11/09	80	1

	2/27/18	1		4/7/12	59	4
8	8/3/17	27	doberman pinscher	7/15/09	85	3
50	3/2/18	80	Miniature australia	11/15/12	52	3
21	5/22/17	16		5/5/07	109	4
38	4/1/18	92	Pembroke Welsh Co	6/15/07	117	4
20	3/12/19	1		10/12/12	66	3
39	5/22/17	1		2/14/05	136	4
46	4/17/18	94	dachshund	8/6/12	57	3
47	9/19/17	86	greyhound	10/7/10	72	4
		82	miniature schnauze	11/20/09	83	3
26	2/27/18	97	miniature pinscher	3/10/08	108	3
38	7/29/17	84	schnauzer	8/25/04		4
15	2/5/18	67	west Highland Whi	7/11/10	79	4
29	1/25/18	63	toy poodle	7/22/10	91	3
30	11/8/17	70	cairn Terrier	12/21/91		3
23	8/22/17	82	miniature schnauze	4/15/11	65	4
42	10/31/19	75	Shih tzu	4/1/11	91	4
34	5/10/17	1		11/2/11	55	4
		1		10/31/11	64	4
	10/3/17	34	Siberian husky	1/13/12	58	4
33	1/25/18	17		6/4/11	68	3
47	3/11/19	81	Australian cattle do	5/13/13	59	3
39	3/8/18	17		7/17/06	128	3
		1		5/6/10	82	4
33	2/26/18	79	tibetan terrier	6/12/11	69	4
33	6/5/17	2		5/20/05	134	4
3	11/7/17	1		2/13/12	58	3
6	1/26/18	1		4/16/11	70	3
3	8/13/17	3		1/11/11	69	3
38		1		9/7/11	61	3
29	7/12/17	97	miniature pinscher	4/5/07	112	3
38	5/22/17	70	cairn Terrier	1/1/07	115	4
38	2/28/19	1		2/26/12	73	3
18	8/3/17	95	shiba Inu	6/8/10	77	4
4	8/1/17	37	irish setter	1/7/11	67	2
47	1/27/18	70	Cairn terrier	5/17/06	129	3
9	5/22/17	1		5/27/05	132	3
	9/6/17	95	shiba Inu	10/10/10	69	3
40	2/6/18	75	Shih tzu	4/30/04	155	3
8	5/10/19	99	basset Hound	12/15/11	78	3
35	9/12/17	27	doberman	3/20/11	66	3
8	7/8/18	94	Dachshund	4/11/10	87	4
38	12/5/17	97	miniature pinscher	3/23/07	117	4
4	5/25/17	2	Japanese Spitz	5/24/06	121	3
14	11/9/17	64	miniature poodle	11/2/11		3

40	3/12/19	81	Australian cattle dc	12/12/12	64	1
38	6/8/17	71	cavalier King Charle	3/2/06	124	4
8	9/5/17	70	cairn Terrier	4/2/07	114	3
46	9/12/17	6		2/15/07	116	4
7	2/1/18	78	pomeranian	4/1/07	107	3
43	2/5/18	1		2/5/04	157	3
4	3/11/19	1		4/1/11	84	3
50	6/29/17	34	Siberian husky	7/29/07	108	4
33	2/13/18	82	miniature schnauze	1/25/10	85	4
35	12/6/18	1		5/1/09	97	4
30	7/21/17	17		5/10/09	87	3
4	1/20/18	92	pembroke Welsh Co	9/10/07	113	4
8	7/28/17	56	border collie	9/7/10	72	4
38	8/1/17	28	rottweiler	1/21/11	67	4
18	1/19/19	28	Rottweiler	1/8/10	97	4
42	7/23/18	82	miniature schnauze	5/13/09	99	3
1	1/12/18	6		9/30/11	65	1
48	1/21/18	77	bichon Frise	7/11/10	80	3
	6/1/19	97	miniature pinscher	4/13/06	146	4
38	9/17/19	82	miniature schnauze	11/6/10	95	3
8	2/27/18	1		2/10/10	85	4
47	9/9/18	71	cavalier king charle	1/1/07	130	3
30	8/29/17	67	west highland whit	5/19/05	136	4
13	12/6/17	97	miniature pinscher	11/22/10	69	4
38	8/7/17	51	german short hair p	5/15/08	99	4
4	5/20/17	8	french bulldog	8/8/08	94	3
20	2/27/18	99	basset Hound	11/1/11	64	4
43	5/9/17	6		1/25/08	100	4
12	1/24/19	1		1/24/12	73	3
23		72	yorkshire Terrier	10/20/11	108	4
8	2/9/19	92	pembroke Welsh Co	9/14/12	60	4
	11/14/17	5		5/12/10	79	3
20	2/7/18	83	schnauzer	1/27/10	97	3
47	6/19/17	51	german shorthair P	11/5/08	85	4
		34	Siberian husky	6/3/10	81	3
1	1/17/18	69	keeshond	11/2/08	99	4
48	3/8/18	77	bichon	5/17/03	159	4
4	1/16/18	63	toy poodle	8/24/09	89	3
23		22	boxer	8/11/08	103	4
21	7/6/17	23	weimaraner	4/3/09	88	4
24	2/26/18	1		4/1/08	108	3
5	7/26/17	1		7/20/09	85	4
22	5/22/17	1		11/24/10	114	3
30	7/16/18	62	standard poodle	1/12/12	67	3
38	1/26/18	72	yorkshire terrier	6/5/07	140	4

38	8/3/17	27	doberman Pinscher	6/29/08	98	4
8	9/5/17	17		5/1/09	88	3
8	8/23/17	84	schnauzer	2/14/11	67	3
8	9/5/17	17		11/13/08	95	3
21	7/27/17	72	yorkshire Terrier	6/1/10	74	4
15	5/16/18	1		12/1/05	151	4
30	2/18/18	6		1/18/07	122	3
9	6/18/17	5		9/20/08	94	4
38	7/26/17	6		10/1/05	130	3
49	7/27/17	2	Schipperke	5/20/10	75	3
23	6/5/17	60	portuguese Water I	8/10/10	81	4
1	5/20/17	66	beagle	5/14/08	97	3
32	9/12/17	1		10/10/10	72	3
46	4/28/19	17		3/30/10	98	3
4	9/27/17	1		10/27/10	72	3
8	6/5/17	77	bichon Frise	6/12/07	108	3
38	11/6/17	68	maltese	8/27/05	135	3
3	5/28/18	1		5/1/08		4
21	6/3/17	6		4/17/09	87	3
29	7/10/18	71	cavalier King Charle	7/30/05	144	4
45	5/29/17	1		3/14/06	123	3
3	6/5/17	17		7/4/10	72	4
38	8/26/18	94	dachshund	8/28/11	73	3
	8/3/17	97	miniature pinscher	5/2/10	76	4
48	2/26/18	6		3/1/10	83	
8	3/12/19	28	rottweiler	3/7/12	73	4
26	1/8/18	1		6/1/06	128	3
21	10/7/17	6		6/23/08	101	3
23	3/12/19	6		6/1/11	143	4
8	11/5/17	3		1/23/04	152	3
21	7/31/17	17		7/17/10	73	3
11	7/18/18	97	miniature pinscher	8/1/11	192	3
14	3/6/18	49	Old english sheepd	2/28/11	73	3
46	7/17/17	74	havanese	8/1/08	96	3
21	1/4/18	17		5/1/07	109	4
46	7/12/17	97	miniature pinscher	6/26/02		3
20	12/30/19	1		1/1/13	73	3
38	12/21/17	1		1/16/10	84	
	3/11/19	6		4/20/09	108	3
20	5/25/17	10		6/16/07	108	3
30	8/24/18	1		5/10/10		4
43	2/8/18	1		1/10/11	74	4
48	9/12/17	1		6/1/08	100	3
17	7/12/17	97	miniature pinscher	12/1/09	80	4
4	3/11/19	1		1/1/10	97	1

30	7/21/17	6		12/30/08	92	3
38	8/25/18	16		7/26/10	86	3
4	5/22/17	1		4/4/07	110	4
43	8/16/17	82	miniature schnauze	5/27/08	100	4
47	12/5/17	1		9/27/10	112	4
8	6/5/17	91	jack russell Terrier	11/29/09	104	4
3	7/28/17	17		6/19/09	86	4
47	11/9/17	74	havanese	8/22/10	75	3
21	6/29/17	34	Siberian husky	10/17/07	105	4
17	2/26/18	94	Long haired dachsh	10/25/10	77	4
42	2/12/19	32	Great dane	10/28/11	77	4
43	4/30/17	5		2/12/03		4
38	7/24/18	10		8/1/09	97	4
48	9/13/17	16		2/22/10	79	3
5	10/23/17	16		7/14/09	87	4
30	11/29/17	15		8/13/03	157	3
9	2/18/18	5		7/17/07	116	2
4	2/5/18	1		11/15/08	99	3
42	1/5/18	67	west highland terri	5/8/09	97	4
23	2/5/18	1		12/8/07	110	3
43	6/30/17	34	Siberian husky	4/10/08	99	4
35	6/8/19	1		1/1/12	79	4
13	1/25/18	74	havanese	7/29/09	115	4
26	8/28/17	17		12/31/09	93	3
32	6/29/17	34	Siberian husky	8/22/06		1
42	9/12/17	72	yorkshire terrier	9/9/09	85	4
23	2/27/18	1		4/15/09	95	4
46	6/15/18	82	miniature schnauze	7/3/10	84	4
4	9/13/17	2	Wirehaired Pointing	8/24/09	85	1
42	5/20/17	67	west Highland Whi	5/16/08	97	3
46	3/12/19	1		9/20/10	102	3
47	1/25/18	5		7/31/09		3
13	5/22/17	67	west highland whit	11/17/09	79	3
8	8/3/17	97	miniature pinscher	10/12/06	109	3
8	8/16/17	82	miniature schnauze	4/10/09	89	3
42	1/16/19	3		9/11/04	161	3
38	6/24/17	77	bichon	4/1/07	111	4
23	2/27/18	67	west Highland Whi	6/20/05	142	4
48	1/16/18	69	keeshond	9/7/07	113	1
5	5/22/17	1		7/23/09	83	3
4	5/22/17	1		8/15/09	70	3
22	8/12/17	17		2/14/08	103	4
	12/12/17	75	Shih tzu	6/12/06	127	3
20	2/1/18	4		7/2/10	80	2
33	12/1/18	74	havanese	10/10/10	87	4

20	8/14/18	62	standard poodle	6/21/07	123	3
38	2/27/18	17		5/25/10	82	4
38	10/30/18	17		3/19/11	80	3
40	7/31/18	82	mini schnauzer	9/4/10	83	3
26	9/3/18	2	Spanish Water Dog	7/11/05	147	4
	2/27/18	94	Dachshund	5/22/10	82	3
		1		4/18/07	110	3
47	1/24/18	97	Minature pinscher	5/25/10	82	4
46	1/21/18	1		2/6/10	85	3
	9/7/18	15		4/15/10	90	1
43	1/28/18	67	west Highland Whi	5/5/05	141	4
38	7/7/17	68	maltese	8/11/09	84	3
7	11/8/17	2	Biewer Terrier	6/14/07	114	3
34		84	schnauzer	8/30/10	80	4
38	9/6/19	17		1/23/11	93	1
38	4/17/17	6		4/10/06	121	4
17	5/23/17	1		3/5/06	123	3
30	1/20/19	3		3/10/11	81	3
15	6/28/17	83	schnauzer	11/25/04	140	4
43	2/26/18	94	Dachshund	6/1/06	133	4
1	1/26/18	84	schnauzer	10/16/08	100	4
	2/8/18	17		6/30/07	115	3
43	5/22/17	84	Chihuahua	2/3/07	112	3
38	7/20/17	1		1/7/03	163	3
32	2/8/18	83	Schnauzer	4/10/09	95	4
7	3/5/18	17		3/20/09	96	3
32	2/10/18	33	malamute	7/2/08	105	3
9	9/28/17	1		5/16/09		3
	3/1/19	3		3/27/10	96	4
23	9/13/17	22	boxer	8/11/08	98	4
17	3/12/19	82	miniature schnauze	6/25/09	106	4
42	5/12/18	37	irish setter	5/17/10	85	3
49	9/12/17	64	miniature poodle	12/3/01	179	3
14	2/9/18	33	Alaskan Malamute	3/4/93		
9	12/3/17	27	doberman pinscher	12/11/09	93	3
18	6/14/17	65	shetland sheepdog	7/18/05	132	4
7	8/24/17	67	west Highland Whi	9/11/09	84	3
18	6/21/17	34	Siberian husky	5/9/08	98	4
21	5/22/18	92	pembroke welsh co	6/1/07	120	4
49	6/5/17	59	English springer	5/2/09	86	3
38	11/23/17	67	west Highland Terri	8/23/08	100	4
38	8/2/17	37	irish setter	9/1/07	108	4
28	7/28/17	5		5/17/07	109	3
38	8/19/17	1		12/1/08	93	3
38	6/17/19	82	miniature schnauze	1/8/09	114	3

43	5/22/17	97	miniature pinscher	2/17/06	124	4
		4		1/1/07	109	4
		97	miniature pinscher	3/18/09	88	4
25	6/5/17	77	bichon Frese	4/1/09	87	4
27	6/5/17	1		6/19/06	120	4
34	6/19/17	51	german shorthairec	6/13/01	181	3
42	2/7/18	83	schnauzer	11/1/09	99	3
16	2/27/18	97	miniature pinscher	1/1/04	163	3
40	8/2/17	37	irish setter	8/12/08	96	1
38	2/27/18	84	Chihuahua	6/5/09	93	3
3	5/22/17	2		5/11/09	85	4
30	10/18/17	15		5/25/09	100	2
23	3/22/19	17		10/10/10	90	3
33	3/25/19	1		1/17/11	88	4
48	3/11/19	92	pembroke Welsh Co	12/12/10	111	2
46	9/28/17	1		8/1/07	109	4
33	6/19/17	26	vizsla	10/18/08	93	3
38	5/10/17	51	german shorthairec	2/21/07	114	3
38	2/7/18	60	portuguese Water I	12/5/04	147	3
38	10/31/19	1		4/25/08	127	3
33	6/11/19	94	dachshund	11/23/10	92	4
23	2/5/18	1		6/6/09	93	3
	1/27/18	6		11/18/08	87	3
47	6/7/18	84	Chihuahua	10/8/09	93	3
44	2/10/18	1		12/10/09		3
30	10/9/17	17		10/30/07	120	3
49	2/27/18	1		5/1/09	46	4
30	8/14/18	2	Polish Lowland She	5/12/10	90	4
38	1/11/19	1		5/8/10	105	3
		3		11/4/09	88	3
34	5/10/17	6		5/25/08	96	3
33	1/13/18	1		2/1/02	167	3
9	5/22/17	67	west Highland Whi	5/10/06	121	4
33	9/5/17	17		6/11/06	124	3
4	5/22/17	1		9/10/08	92	4
40	10/31/19	17		1/5/08	127	4
38	4/3/19	68	maltese	1/1/09	120	4
48	3/22/18	1		12/10/09	97	3
38	9/11/17	17		7/21/07	111	3
38	5/25/17	1		4/1/08	98	3
34	9/14/18	47	bullmastiff	6/1/07	112	4
24	9/23/18	7	boston terrier	4/12/10	90	4
30	7/26/17	96	welsh terrier	1/11/08	103	3
44	3/19/18	1		9/10/08	103	4
46	6/14/17	82	miniature schnauze	10/16/08	93	3

4	6/13/17	97	miniature pinscher	8/16/04	154	4
7	8/3/17	97	miniature pinscher	1/31/99	167	3
30	1/28/18	76	Lhasa apso	3/6/07	119	3
21	5/24/17	91	jack russell terrier	1/21/09	89	4
		97	Miniature pinscher	11/8/06	117	4
30	5/22/17	2		9/8/08	93	3
46	8/11/17	1		3/23/09	85	3
3	1/24/18	82	miniature schnauze	5/19/06	128	4
5	5/9/17	2	Xoloitzcuintle (Mex	4/1/08	103	3
24	7/27/17	17		10/6/08	95	4
5	6/5/17	81	Australian cattle do	5/1/08	89	3
	2/27/18	64	miniature poodle	6/4/05	153	3
43	8/9/18	1		1/1/10	93	4
30	10/19/18	6		4/5/10	91	3
48	1/15/19	3		10/4/06	136	3
	4/14/18	33	Alaskan malamute	10/10/06		2
35	2/7/18	82	miniature schnauze	8/21/09	91	4
38		1		3/21/08	87	4
1	3/13/19	1		9/3/08	115	4
30	2/7/18	82	miniature schnauze	7/4/06	129	4
4	4/14/18	1		10/9/09	91	3
	2/8/18	82	miniature schnauze	3/1/06	132	4
4	3/11/19	68	maltese	8/27/08	115	4
11	2/5/18	6		2/5/08	109	3
11	7/11/17	4		12/21/05	127	3
12	6/22/18	92	Welsh pembroke Co	5/31/09	98	4
8	2/26/18	82	miniature schnauze	7/14/08	104	4
46	5/11/17	5		9/20/08	93	4
43	5/23/17	72	yorkshire Terrier	3/10/08	99	3
20	2/26/18	77	bichon Frise	10/20/08	101	3
20	3/11/19	1		10/14/07	126	4
2	5/20/17	1		10/10/07		4
13	3/26/19	1		8/7/08	116	4
16	9/6/17	1		2/2/07	115	3
23	9/24/18	5		2/17/10	104	3
38	5/20/18	71	cavalier King Charle	4/19/09	98	4
32	1/25/18	1		5/31/05	139	3
33	3/11/19	7	Boston Terrier	9/4/06	139	3
33	2/7/18	1		6/13/08	104	3
	5/22/17	101	brussels griffon	1/11/08	103	3
38	7/29/18	97	Miniature Pinscher	12/8/08	116	4
9	8/2/17	36	english setter	11/1/06	106	4
38	3/12/19	67	west Highland Terri	1/8/10	99	3
4	2/12/18	17		9/23/08	90	4
	4/26/17	5		6/20/07	107	1

48	12/5/18	17		2/7/10	95	4
46	5/22/17	97	miniature pinscher	4/28/08	98	3
15	6/5/17	1		6/9/05	132	3
30	6/26/17	17		6/2/08	95	4
43	3/11/19	1		4/4/10	96	3
30	2/7/18	63	toy poodle	5/16/05	154	3
38	1/4/18	1		7/18/08	103	4
20	5/25/17	97	miniature pinscher	2/29/08	99	3
28	1/25/18	64	miniature poodle	3/9/09	107	2
26	6/5/17	2	Belgian Tervuren	4/3/08	99	3
40	2/5/18	3		7/7/07	116	4
38	2/5/18	2	Norfolk Terrier	5/3/08	106	3
34	5/22/17	1		4/5/06	122	3
38	8/4/17	6		3/23/08	102	3
3	8/2/17	17		9/5/06	120	3
30	2/26/18	94	Miniature dachshur	6/1/06	128	3
4	8/15/18	1		1/1/09	103	3
28	2/5/18	97	miniature pinscher	10/15/08	100	4
34	5/10/17	17		1/27/08	101	4
		82	miniature schnauze	12/19/08	97	3
19	5/22/17	6		8/1/06	114	3
9	4/28/18	70	cairn Terrier	4/5/07	121	4
7	4/17/17	1		3/5/07	110	3
43	5/22/17	1		4/3/07	110	3
	5/22/17	72	yorkshire terrier	7/27/06	117	4
35	9/13/17	6		12/1/07	106	
49	8/9/17	82	miniature schnauze	12/29/07	117	4
21	5/11/18	1		6/4/09	96	4
38	3/14/19	3		4/3/08	133	3
11	2/27/18	66	beagle	1/29/09	99	3
49	2/27/18	94	dachshund	1/26/08	110	3
21	6/12/18	94	dachshund	6/23/09	96	4
18	5/22/17	34	Siberian husky	11/28/07	115	3
32	8/3/17	72	yorkshire Terrier	4/1/07	113	3
34	6/5/17	15		8/19/07	107	4
	8/2/19	2		1/14/09	116	3
38	8/29/17	17		5/12/08	100	2
38	6/14/17	1		6/14/05	133	4
46	8/3/17	63	toy poodle	9/25/05	131	3
21	5/28/18	67	west Highland Whi	9/1/04	153	3
	11/24/18	56	border collie	11/24/08	109	4
40	8/1/17	28	rottweiler	2/18/08	102	4
4	1/3/18	1		3/1/07	116	4
1	3/13/18	73	scottish terrier	12/26/06	124	3
32	8/28/17	1		12/22/02	165	4

38	7/12/17	1		12/12/06	117	4
18	11/9/17	3		10/30/04		3
30	1/25/19	75	Shih tzu	10/26/09	101	1
2	5/22/17	1		3/12/08	99	3
38	12/10/19	97	Miniature pinscher	5/21/10	103	3
43	9/11/17	1		4/7/06	126	3
		7	boston terrier	6/5/07	9	4
2	7/13/17	63	toy poodle	5/7/08	99	4
39	1/24/19	99	basset hound	5/24/09	105	3
14	9/12/17	67	west highland whit	4/16/07	114	3
23	6/16/17	59	English springer Sp	6/26/07	108	4
5	7/27/17	17		1/5/08	103	1
18	5/9/17	94	dachshund (Dapple	5/8/05	145	4
46	6/7/17	1		6/6/07	109	3
19	2/2/18	73	scottish Terrier	2/24/07	120	4
9	11/7/17	3		8/20/08	100	3
21	4/16/18	63	toy poodle	7/12/07	130	3
38	4/17/17	28	rottweiler	12/20/07	102	3
4	7/22/18	1		8/28/08	108	3
13	4/9/18	17		1/17/09	100	3
46	1/16/19	3		10/24/09	123	4
33	3/4/19	1		11/20/09	100	3
48	2/28/18	49	Old english sheepd	4/23/07	119	3
13	6/5/17	75	Shih tzu	2/19/08	100	4
22	7/12/17	94	dachshund	10/1/07	106	3
3	5/29/18	4		7/8/08	119	3
43	9/20/17	67	west Highland Whi	2/18/05	140	4
46	6/30/19	6		12/26/09	103	3
5	9/5/17	1		6/29/06	124	4
13	2/28/18	1		10/20/08	103	3
14	1/22/18	1		5/1/08	107	3
7	5/20/17	70	Cairn terrier	6/1/06	132	4
8	4/23/18	17		11/3/07	112	4
15	8/31/17	6		3/3/06	126	3
30	2/27/18	71	cavalier King Charle	2/16/08	110	3
30	11/6/17	6		11/24/06	120	4
26	1/4/18	78	pomeranian	7/8/06	127	3
42	7/25/17	1		1/1/08	97	4
17	11/3/18	72	yorkshire Terriers	4/10/08	116	3
28	10/29/17	82	miniature schnauze	6/19/08	101	3
14	3/12/19	67	west Highland Whi	8/15/09	103	3
38	5/31/17	67	west Highland Whi	2/11/06	124	3
1	7/29/19	68	maltese	8/15/07	132	4
13	8/23/17	94	Miniature Long Hai	7/24/05	134	4
42	2/16/18	1		9/8/07	114	4

4	8/17/18	2	Wirehaired Pointing	3/17/09	97	2
38	11/19/17	1		8/22/05	135	4
	5/3/17	5		6/1/06	121	4
30	2/7/18	1		2/14/08	109	4
30	6/13/18	1		6/10/08	109	3
		82	miniature schnauze	7/7/07	123	4
46	10/11/18	17		5/26/08	114	3
18	11/1/19	1		11/1/09	109	3
	2/27/18	82	mini schnauzer	4/22/08	107	4
13	6/5/17	17		8/21/07	105	3
43	7/13/17	17		1/3/08	103	2
19	9/11/17	82	miniature schnauze	10/14/04	144	3
13	5/22/17	1		3/27/07	111	4
38	7/25/17	91	jack russell Terrier	8/5/05	132	4
20	2/27/18	1		5/24/08	116	3
18	8/30/19	92	Pembroke Welsh Co	4/27/09	113	3
	9/27/17	2	Redbone Coonhoun	5/15/07	113	4
5	7/18/17	17		12/3/05	128	1
34	4/22/19	22	boxer	7/4/08	115	3
20	5/24/17	1		4/1/00	195	4
38	1/16/19	1		5/25/07	141	4
48	1/6/18	82	miniature schnauze	5/24/08	103	3
42	2/5/18	98	basenji	1/4/07	122	3
20	11/15/17	82	miniature schnauze	1/19/07	119	3
30	7/26/17	101	brussels Griffon	11/9/05	129	3
26	2/8/18	78	pomeranian	3/1/08	108	4
30	1/8/19	1		10/21/08	124	3
33	5/22/17	78	pomeranian	12/1/04	138	3
		1		7/11/06	119	4
20	6/8/17	76	Lhasa apso	4/3/05	135	4
47	2/15/18	5		7/31/06	131	1
43	2/27/18	1		6/5/08	106	4
20	4/28/19	1		6/9/09	107	3
43	2/5/18	1		4/1/07	119	3
6	5/20/17	72	yorkshire Terrier	3/12/06	123	3
13	6/5/17	92	pembroke Welsh Co	4/30/07	110	3
9	7/20/17	1		5/1/06	123	3
21	2/26/18	1		5/11/08	104	3
	4/8/18	54	rough collie	9/15/07	128	4
38	7/27/17	1		5/1/05	135	4
18	1/9/18	57	cocker spaniel	11/7/06	123	3
47	2/8/18	1		6/15/07	117	4
	8/16/18	17		3/27/08	114	3
8	5/20/17	77	bichon Frise	6/16/04	155	4
		5		12/14/05	133	3

38	8/8/17	34	Siberian husky	5/1/07	112	3
38	8/31/17	92	pembroke welsh co	10/14/06	121	3
4	3/20/18	63	toy poodle	3/15/07	121	3
48	2/27/18	77	bichon Frise	2/12/08	109	3
2	5/22/17	1		9/16/06	119	3
43	9/11/17	2	Rat Terrier	2/4/06	128	4
	8/11/17	3		9/20/02	168	3
18	9/20/17	1		11/11/04	142	3
43	12/21/18	17		1/1/07	121	4
20	10/16/19	82	miniature schnauze	6/1/09	113	4
9	5/22/17	1		3/17/07	111	4
33	3/28/18	73	scottish Terrier	4/8/03		4
42	3/5/18	84	schnauzer	11/15/04	149	3
21	3/1/18	37	irish setter	3/28/08	108	2
8	7/2/17	97	miniature pinscher	7/24/07	108	4
38	12/27/17	92	pembroke Welsh Co	9/7/07	112	3
13	2/4/18	73	scottish Terrier	7/17/69		3
38	11/18/18	6		10/17/08	110	3
38	2/7/18	67	west Highland Whi	4/28/91	218	4
30	3/10/18	77	bichon Frise	4/27/07	120	4
13	1/30/18	3		2/14/04	151	3
4	6/10/19	3		6/16/09	109	4
38	1/16/18	1		6/15/03	176	4
20	2/27/18	34	Siberian husky	2/21/08	109	4
48	10/20/17	1		10/14/07	103	2
22	5/3/17	5		5/5/06	121	4
14	7/20/17	57	cocker spaniel	2/3/03	162	3
4	1/8/19	72	yorkshire TERRIER	1/1/07	133	4
11	12/6/19	92	pembroke Welsh Co	1/1/07	133	4
23	4/29/17	82	mini schnauzer	8/14/06	117	3
5	1/24/18	62	standard poodle	2/3/03		3
26	8/12/17	79	tibetan terrier	12/15/05	129	2
30	2/26/18	1		3/26/07	121	4
30	8/25/17	1		4/11/07	113	4
4	1/29/18	1		12/8/07	110	3
13	2/27/18	31	akita	4/15/07	119	4
30	3/8/18	1		1/2/08	115	3
12	8/16/17	34	Siberian husky	12/6/06	117	4
38	10/31/19	72	yorkshire Terrier	2/10/08	129	3
38	2/10/18	2		10/1/07	109	3
38	2/22/18	17		7/11/07	116	3
24	9/5/17	67	west Highland Whi	4/12/04	150	4
1	7/28/17	1		1/1/03	157	3
46	9/27/19	1		2/14/06	152	3
34	2/27/18	1		2/27/06	133	3

18	9/13/17	67	west Highland Whi	6/13/04	148	3
23	3/12/19	82	miniature schnauze	8/1/08	116	3
33	3/12/19	1		4/24/08	120	3
32	9/19/19	3		11/24/07	132	3
38	8/31/19	17		4/10/08	125	4
37	6/30/17	34	Siberian husky	3/28/06	124	4
13	6/15/17	26	vizsla	7/29/05	132	3
7	3/11/19	6		5/10/07	131	4
8	7/12/17	77	bichon Frise	7/1/03	152	4
38	2/8/18	2	Puli	2/5/02	181	4
6	2/15/19	2	Silky Terrier	9/11/06	138	3
21	9/12/17	1		5/27/07	112	4
13	1/25/18	6		7/18/07	115	4
38	8/11/17	1		2/15/06	127	3
16	2/5/18	1		7/1/07	117	4
1	2/27/18	90	Norwich terrier	7/13/07	109	4
40	11/8/19	7	boston terrier	7/9/09	113	4
5	1/30/18	100	Irish Terrier	1/1/07	121	3
38	7/26/19	1		6/12/08	122	4
18	1/17/18	1		8/1/05	138	3
30	6/10/19	62	standard poodle	7/4/08	120	3
38	7/12/18	1		2/13/08	114	3
34	1/25/18	63	toy poodle	8/6/07	114	3
4	6/27/17	17		1/4/07		
38	6/14/19	1		8/26/08	118	3
	5/22/17	74	havanese	5/5/05	133	3
23	2/8/18	82	miniature schnauze	8/18/04		4
34	6/30/17	34	Siberian husky	1/1/06	127	4
41	8/25/19	92	pembroke Welsh Co	5/1/06	148	4
38	8/10/18	64	miniature poodle	12/13/07	117	3
47	11/8/17	16		6/24/05	138	4
4	2/27/18	72	yorkshire Terriers	12/25/06	123	3
13	1/4/18	1		5/17/07	116	4
8	2/26/18	1		7/15/03	164	3
34	5/22/17	97	miniature pinscher	10/18/06	114	3
46	8/3/17	37	irish setter	9/4/06	120	4
30	2/7/18	82	miniature schnauze	6/1/07	122	3
38	6/20/17	74	havanese	11/3/06	116	4
	1/18/19	3		6/3/07	127	4
38	6/29/17	6		12/1/05	133	4
21	2/8/18	33	Alaskan malamute	3/12/00	205	4
9	7/3/18	93	cardigan Welsh Cor	3/29/06	136	2
7	9/28/17	15		1/2/07	119	3
34	12/13/17	92	pembroke Welsh Co	12/5/05	133	3
30	6/18/18	6		10/10/07	130	3

9	8/16/17	82	miniature schnauze	7/1/06	121	4
		2	Redbone Coonhoun	5/1/06	125	4
4	6/5/17	83	schnauzer	9/20/06	117	3
	7/30/17	1		1/13/06	127	4
14	9/11/17	1		11/8/06	119	3
38	5/29/18	1		6/15/06	132	4
38	5/15/17	80	australian shepherc	2/12/04	148	3
22	1/26/18	97	miniature pinscher	12/20/06	122	4
5	12/23/18	1		6/14/06	139	3
33	3/11/19	51	german shorthair P	5/5/06	142	3
34	5/11/17	17		9/1/04	137	2
4	7/12/17	77	bichon Frise	12/13/06	115	4
30	11/5/17	67	west Highland Whi	4/7/07	116	3
24	9/5/17	1		1/1/06	129	3
13	3/11/19	99	basset Hound	7/1/08	117	3
22	2/6/19	92	pembroke welsh co	6/15/08	104	2
	3/11/19	67	west highland whit	6/1/07	130	3
26	3/11/19	1		7/1/08	121	3
38		6		5/5/04	155	3
15	3/11/19	1		2/24/08	122	4
38	3/19/19	3		4/29/04	167	3
23	9/13/17	67	west Highland Terri	1/1/06	130	4
38	4/18/17	84	chihuahua	3/1/06	122	4
	7/27/17	54	rough collie	9/14/06	119	4
33	2/7/18	82	miniature schnauze	4/1/06	131	3
31	2/26/18	1		4/1/06	131	4
30	3/25/18	1		3/1/06	133	3
3	1/28/18	93	cardigan Welsh Cor	3/15/07	119	4
	9/30/17	17		8/24/06	122	4
34	2/27/18	1		3/17/07	120	4
42	12/5/18	1		12/5/05	145	4
		1		11/1/07	122	3
21	2/4/18	82	miniature schnauze	6/27/05	140	4
34	2/26/18	1		12/13/05	135	4
30	4/17/17	77	bichon	5/10/06	120	4
		76	Lhasa apso	2/19/06	114	4
38	7/29/17	69	keeshond	8/2/05	132	4
4	5/22/17	1		4/26/06	122	4
38	9/10/19	91	jack russel Terrier	9/1/08	121	4
47	3/5/19	1		11/1/07	125	4
30	4/18/17	77	bichon Frise	4/14/03	157	4
26	9/9/18	76	LHASA apso	11/9/06	131	4
33	10/31/19	68	maltese	1/15/06	143	4
		82	miniature schnauze	8/5/07	134	4
8	1/3/18	78	pomeranian	1/3/07	109	3

	1/26/18	1		1/1/07	133	4
4	8/2/17	62	standard poodle	4/10/05	136	4
33	3/3/18	1		2/14/07		
38	12/1/19	67	west Highland Whi	6/21/06	151	3
17	8/11/17	77	bichon Frise	5/19/06	124	3
38	6/28/17	7	boston terrier	10/9/05	130	4
38	9/10/18	82	Miniature Schnauze	5/5/07		
38	5/21/19	67	west highland whit	3/27/08	124	3
33	1/24/18	1		4/20/06	130	4
11	2/5/18	91	jack russel	4/24/04	155	4
7	8/19/17	77	bichon FRISE	7/4/05	134	3
20	3/16/19	16		1/1/05		4
8	10/20/17	6		8/6/06	123	4
		1		4/8/01	184	4
38	3/7/18	23	weimaraner	3/23/05	144	3
26	6/5/17	1		1/1/06	127	3
5	9/5/17	71	cavalier King Charle	5/13/06	125	4
35		31	akita	3/13/06	126	4
46	4/9/18	1		1/1/07	125	3
38	5/9/19	3		12/1/05	150	3
11	2/5/18	2		6/6/06	121	3
4	5/22/17	91	jack russell Terrier	5/4/04	145	4
30	10/28/17	1		2/11/06	129	3
49	1/6/18	17		7/4/05	138	4
18	2/27/18	94	Miniature dachshur	9/15/06	126	3
21	8/11/17	80	Australian Shepher	4/1/03	161	3
9	12/7/19	72	yorkshire Terrier	12/6/07	133	3
20	2/21/18	70	cairn terrier	11/24/02	157	3
30	6/17/18	17		1/12/07	126	1
21	2/27/18	97	Miniature pinscher	5/28/05	142	4
18	8/2/17	1		12/12/04	137	3
10	6/5/17	1		11/1/05		
5		1		2/14/05	144	4
4	2/9/18	34	Siberian husky	6/24/06	129	4
30		68	maltese	12/22/06	136	3
24	9/5/17	1		7/1/04	147	4
30	9/8/19	76	Lhasa apso	2/26/08	126	3
0	6/2/18	34	Siberian husky	11/3/06	127	4
8	5/22/17	80	australian shepherc	6/30/05	133	4
30	4/6/18	2	Std Manchester ter	6/30/04	154	4
8	6/5/17	77	bichon	6/6/05		4
23	9/9/18	6		8/1/05	146	3
0	11/6/17	3		1/18/06	131	3
9	2/1/18	74	havanese	12/6/03	170	3
0	11/6/17	3		1/18/06	130	3

37	5/22/17	67	west highland whit	6/14/03	156	4
	1/25/18	62	standard poodle	5/25/05	141	4
34	2/8/18	82	miniature schnauze	3/26/06	132	4
9	5/22/17	1		6/11/05	132	3
46	2/4/18	73	scottish Terrier	7/1/05	140	4
37	3/11/19	1		8/23/06	139	4
6	1/16/18	69	keeshond	5/27/05	129	4
4	3/11/19	6		9/12/05	151	3
38	1/26/18	1		5/15/04	153	4
8	1/17/19	2	Silky Terrier	9/2/02	197	1
8	9/14/17	34	Siberian husky	6/1/04	148	4
4	9/11/17	94	dachshund	4/1/03	136	3
42	9/23/17	2	Toy Manchester Te	7/22/04	147	3
18	1/27/18	70	cairn Terrier	2/11/05	132	3
38	9/18/17	94	dachshund	5/25/05	137	3
47	8/28/17	1		11/1/05	130	4
33	7/31/17	67	west Highland Whi	4/19/05	136	3
46	5/20/17	1		12/1/02	162	4
38		1		5/25/07	133	3
34	10/27/17	1		7/24/03	160	4
43	9/12/17	2	Clumber Spaniel	7/5/05	135	4
32	2/26/18	82	miniature schnauze	7/25/05	140	3
		7	boston terrier	7/25/05	133	3
21	6/5/17	77	bichon Frise	3/7/03		3
47	7/30/17	81	Australian cattle dc	4/15/04	147	3
38	3/13/19	68	maltese	2/5/05	158	4
45	2/7/18	82	miniature schnauze	3/12/05	144	3
38	7/21/17	6		4/21/05	136	4
7	7/20/18	63	toy poodle	7/22/06	132	3
8	3/20/19	72	yorkshire terrier	4/10/07	132	4
25	3/7/19	92	pembroke welsh co	6/21/05	153	4
20	5/19/17	1		2/14/05	136	3
3	6/30/18	2	Coton de Tulear	6/20/06	133	4
30	7/11/17	6		6/28/05	133	4
35		1		5/22/05	133	4
35		94	dachshund	11/9/04		3
34	5/23/17	80	Australian Shepher	10/30/02	163	3
14	8/11/17	68	maltese	4/15/03	161	3
38	11/1/18	94	mini dachshund	9/4/06	133	4
22	10/18/17	1		6/2/03	161	3
38	2/27/18	2	Rat Terrier	12/28/05	135	3
30	3/29/19	1		1/1/07	135	3
47	5/23/17	92	Pembroke Welch C	3/31/05	135	4
30	4/6/18	82	Miniature Schnauze	11/15/05	138	3
38	7/25/17	1		11/24/04	141	3

37	4/4/18	1		1/1/06	138	3
48	11/20/17	3		5/30/95		3
24	9/5/17	63	toy poodle	9/9/04	144	4
38		17		11/5/05	155	4
33	2/26/18	1		11/11/05	136	3
4	11/11/17	84	schnauzer	4/17/02	175	4
19	8/2/17	80	australian shepherc	10/31/03	155	3
42	6/20/17	17		2/14/05	137	4
6	7/21/17	82	miniature schnauze	10/14/04	142	3
47	8/10/18	80	australian shepherc	4/1/06	137	2
38	2/26/18	94	Miniature dachshur	4/18/05	143	4
22	7/11/17	33	Alaskan malamute	8/2/03	155	3
5	8/12/17	3		8/7/04	145	4
4	1/15/19	3		7/1/04	163	3
5	11/9/18	92	pembroke Welsh Co	4/19/04	157	4
13	3/12/19	17		10/31/06	138	4
21	8/29/18	94	dachshund	4/14/06	137	3
21	8/29/17	4		3/21/05	138	3
23	1/30/18	70	cairn Terrier	8/21/04	150	4
6	3/12/19	67	west HIghland Whi	10/23/04	162	3
5	5/20/17	91	Jack Russell Terrier	6/1/04	144	
14	6/20/19	81	Australian cattle dc	1/1/07	139	4
38	6/29/17	60	portuguese Water I	12/21/04	139	4
4	4/27/17	3		11/2/04	136	3
23	5/20/17	1		10/6/03	140	4
33	8/11/17	1	Schnoodle	2/10/05	139	3
20	11/2/18	1		11/5/05	145	3
38	3/7/18	6		7/3/05	130	3
34	4/28/19	1		7/15/06	142	3
9	7/25/17	1		4/1/03	160	4
46	2/26/18	17		5/3/05	143	3
33	9/20/17	6		4/20/04	150	3
38	5/24/17	17		8/3/04	145	3
	7/16/17	36	english setter	9/17/04	143	1
38	4/3/19	67	west Highland Whi	1/14/06	148	3
40	1/4/18	1		3/17/03		3
26	6/18/18	77	bichon Frise	6/19/05	145	4
25	5/22/17	66	beagle	3/23/04	147	3
38	5/19/17	6		4/5/02	170	4
50	9/5/17	94	dachshund	1/1/03	165	3
35	11/21/18	92	Pembroke welsh co	9/21/05	148	4
26	6/4/17	1		12/25/03	115	4
4	9/5/17	67	west highland whit	10/3/03	156	4
38	1/25/19	70	cairn Terrier	7/4/05	151	3
22	5/15/18	26	vizsla	11/20/04	151	4

38	6/2/19	1		11/30/05	151	4
47	8/14/17	1		7/4/03	157	3
17		27	doberman oincher	12/15/04	152	3
		80	australian shepherc	8/16/04	153	3
14	1/27/18	1		10/16/03	160	3
15	2/27/18	1		3/24/04	156	4
	2/5/18	78	pomeranian	6/25/03	177	4
43	7/25/17	84	Chihuahua	8/2/03	156	3
38	6/10/17	1		6/1/03	145	4
26	5/22/17	1		1/28/01	185	4
38	2/2/18	1		3/1/04	156	3
33	5/31/19	17		6/2/05	156	
	6/6/17	67	west Highland Whi	4/8/00	194	3
47	1/25/18	74	havanese	11/8/03	159	4
46	3/1/18	94	dachshund	9/21/01	186	4
16	1/4/18	67	west Highland whit	8/1/03	162	3
46	1/26/18	77	bichon Frise	8/27/02	174	4
38	9/20/18	1		9/4/01	193	3
38	9/28/17	1		5/3/03	164	4
47	7/29/17	17		9/15/02	167	4
5	2/28/18	49	Old english sheepd	3/5/00	85	4
34		92	pembroke welsh co	7/18/02	169	3
4	6/5/18	1		6/5/02	181	4
26	7/1/17	1		12/20/00	187	4
4	5/22/17	1		9/13/01	178	3
18	11/16/17	97	miniature pinscher	3/21/02	178	4
23	7/18/17	15		12/15/01	175	4
39	1/14/18	1		7/4/02	175	3
21	4/8/19	1		4/8/03	81	1
38	5/18/19	1		5/15/00	97	4
30	11/2/19	1		1/23/12	84	3
43	2/18/19	1		10/1/00	102	4
12	7/16/17	37	irish setter	5/12/08	100	3
44	8/2/17	92	pembroke welsh co	7/15/98		3
34	9/7/17	17		1/1/98	106	3
42	12/29/19	1		5/10/00	104	3
	6/5/17	1			120	4
33		6		1/1/07		
	10/24/17	74	Havanese			4
33	8/30/19	64	Mini Poodle	4/14/08		3
13	5/15/17	10		5/15/17	175	4
38	5/16/17	17		1/20/17	89	4
20	5/17/17	17		3/1/17	75	3
		1		5/22/17	135	3
37	5/22/17	2		8/31/07	106	4

	6/5/17	17		5/28/17	109	4
37	6/6/17	72	yorkshire Terrier	4/16/07	123	4
43	6/13/17	1		6/13/17	13	3
18	6/18/17	2		6/18/17	131	4
38	6/20/17	74	havanese	5/6/11	62	3
4	7/6/17	99	basset Hound	3/24/08		4
30	7/6/17	99	Basset hound	1/1/99		
38	7/12/17	6		11/2/17	165	3
13	7/21/17	97	miniature pinscher	7/1/95	109	4
		1		1/1/11	67	3
18	7/24/17	17		1/26/17	67	4
33	7/26/17	67	west Highland terri	2/8/17	139	3
42	7/29/17	5		4/10/10	146	3
	8/1/17	84	schnauzer	8/15/05		3
3	8/2/17	1		8/25/09	84	3
	8/11/17	3		8/12/04	145	3
38	8/22/17	78	pomeranian	12/23/07	105	2
13	9/5/17	71	cavalier KING CHAR	5/13/06	125	4
	9/5/17	32	Great dane	7/28/09	85	3
18	9/12/17	2		3/12/17	55	1
33	10/6/17	6		10/6/17	95	4
34	11/7/17	81	Australian australia	11/12/17	72	4
22	11/8/17	16		3/15/14	33	2
5	11/9/17	70	cairn Terrier	11/10/07	109	3
38	11/26/17	5		6/13/17	55	3
	1/4/18	5		10/31/09		4
4	1/16/18	69	keeshond	4/20/10	80	4
	1/16/18	69	keeshond	10/30/94	151	4
	1/16/18	69	keeshond	5/10/10		3
26	1/17/18	57	american cocker Sp	7/5/04		2
38	1/18/18	28	rottweiler	1/18/18	97	3
8	1/26/18	64	miniature poodle	1/26/18	130	4
48	1/26/18	6		3/1/18	83	3
		2		1/23/98		3
8	2/5/18	77	bichon Frise	6/12/06	129	3
	2/7/18	3		2/7/18	103	3
	2/7/18	3		2/7/16	138	4
34	2/17/18	2	petit basset griffon	6/18/07	117	4
27	2/26/18	1		6/2/18		
13	2/27/18	1		2/2/18	109	3
6	2/27/18	1		3/1/14	156	3
4	2/28/18	37	irish setter	7/16/18	128	4
24	2/28/18	78	pomeranian	5/1/18	106	4
14	3/3/18	49	Old english sheepd	9/15/18	114	3
4	3/7/18	1		1/28/18	110	4

18	3/24/18	67	west Highland Terri	4/4/18	144	3
4	5/9/18	22	boxer	8/11/10	88	4
35	7/7/18	92	pembroke Corgi	2/13/17	126	4
4	8/8/18	80	Australian Shepherd			
42	8/9/18	2		12/27/18		4
18	8/15/18	1		1/21/10	92	3
	9/9/18	56	border collie	5/25/18	89	4
4	9/20/18	1		9/20/18	73	4
8	10/3/18	84	schnauzer	10/3/18	133	4
6	10/15/18	3		1/9/04	163	4
38		1			10	
46	3/5/19	92	pembroke Welsh Co	5/1/19	83	4
17	3/5/19	63	toy poodle	3/2/05	168	3
46	3/11/19	91	jack russell	3/11/19	109	3
38	11/9/19	1		9/2/12	75	4
48	12/13/19	3		8/1/19		

pet_wt	dx_dm	ins_ans	ins_type	instype_othe	ins_freq	ins_units	dm_dxdateformatted
35	1	1	2		1	21	6/10/17
16	1	1	0		1	10	11/20/04
76	1	1	0		1	24	1/9/18
45	1	0					8/10/17
52	1	1	0		1	16	10/1/16
35	1	1	0		1	6	6/26/04
22	1	1	0		1	14	10/5/08
40	1	1	0		1	7	6/26/16
32	1	1	4		1	1	8/24/16
28	1	1	0		1	7	12/12/12
8	1	1	2		1	3	15-May-16
93	1	1	0		1	20	7/1/12
15	1	1	5	Humulin R	2	2	5/1/07
20	1	1	0		1	6	5/2/17
18	1	1	0		1	8	2/1/18
5	1	1	2		1	3	8/5/19
26	1	1	2		1	4	8/17/17
33	1	1	0		1	3	12/26/16
35	1	1	0		1	6	6/22/09
10	1	1	0		1	2	8/11/17
50	1	1	0		1	13	5/1/16
90	1	1	2		1	11	5/1/14
9	1	1	2		1	3	3/1/09
10	1	1	5	Novolin 70/3	1	3	1/26/16
40	1	1	5	Novalin	1	10	12/12/17
62	1	1	0		1	8	10/9/16
60	1	1	5	Protaphane	1	1	12/20/15
13	1	1	2		1	3	3/1/15
22	1	1	2		1	5	7/15/19
86	1	1	0		1	17	8/20/14
12	1	1	0		1	3	4/5/13
25	1	1			1		9/15/12
104	1	1	0		1	17	10/20/12
12	1	1	2		1	4	5/1/09
78	1	1	2		1	13	11/15/11
67	1	1	1		1	8	1/13/17
35	1	1	0		1	6	2/16/08
35	1	1	2		1	6	7/1/18
11	1	1	0		1	3	5/1/09
11.3	1	1	0		1	3	1/1/08
26	1	1	0		1	17	5/24/10
65	1	1	0		1	14	9/26/16
19.5	1	1	0		1	4	4/12/18
42	1	0					9/10/03

65	1	1	2		1	14	12/13/16
37	1	1	2		1	10	7/1/14
18.7	1	1	0		1	2	9/22/16
96	1	1	0		1	17	1/1/14
50	1	1	2		1	9	1/14/09
27	1	1	4		1	2	5/10/04
30	1	1	0		1	10	12/2/17
20	1	1	2		2	2	6/15/16
50	1	0					9/15/08
80	1	1	4		1	10	3/22/12
20	1	0					9/14/18
65	1	1	0		1	29	1/13/11
12	1	1	0		1	4	9/1/08
100	1	1	0		1	35	5/15/14
30	1	1	0		1	7	12/15/15
18	1	1	2		1	2	4/30/15
18	1	1	2		1	6	7/21/16
15	1	1	0		1	2	1/25/17
25	1	1	5	Protaphane	1	6	10/10/16
19.7	1	1	0		1	8	6/1/13
11	1	1	0		1	4	4/13/10
42	1	1	0		1	10	7/4/17
10	1	1	0		1	2	9/18/13
10	1	1	2		1	4	7/10/12
19.4	1	1	0		1	4	1/6/16
19.4	1	1	0		1	4	1/6/16
9	1	1	0		1	2	2/1/11
21	1	1	0		1	6	6/1/15
35	1	1	0		1	10	7/15/16
65	1	1	0		1	14	10/24/16
45	1	1	0		1	20	1/21/11
44	1	1	0		1		10/3/08
	1	1	0		1	11	8/1/11
8	1	1	0		1	1	1/1/17
30	1	1	0		1	14	12/24/13
23	1	1	0		1	8	4/1/17
80	1	1	0		1	23	12/15/16
9	1	1	0		1	5	5/18/16
15	1	1	0		1	6	4/1/08
33	1	1	0		1	6	1/1/17
178	1	1	0		1	4	5/15/15
135	1	1	0		1	33	5/1/17
18	1	1	2		1	6	1/16/13
8.5	1	0					1/2/08
75	1	1	0		1	19	10/12/18

50	1	1	2		1	12	5/15/13
12	1	1	2		1	5	10/3/16
15	1	1	2		2	2	5/1/12
77	1	1	0		1	15	5/25/15
19	1	1	4		1	3	12/1/14
30	1	1	0		0	4	6/1/12
36	1	1	0		1	10	7/2/15
75	1	1	0		1	23	6/21/16
9	1	1	0		1	4	8/14/16
80	1	1	0		1	20	3/1/15
11	1	1	0		1	2	8/20/17
67	1	1	0		1	18	9/1/09
18.4	1	1	0		1	10	1/7/12
27	1	1	0		1	3	6/1/11
35	1	1	0		1	19	2/5/15
17	1	1	0		1	3	5/12/17
58	1	1	0		1	12	10/31/15
25	1	1	0		1	7	12/14/16
119	1	1	0		1	16	10/15/18
9	1	1	5	Caninsulin	1	4	11/1/13
23.3	1	1	0		1	3	11/21/18
10	1	1	2		1	10	6/1/11
120	1	1	0		1	15	2/1/18
40	1	1	0		1	13	4/17/17
88	1	1	0		1	16	4/3/17
35	1	1	2		1	10	11/15/15
45	1	1	2		1	11	6/15/16
23.2	1	1	0		1	6	4/12/17
30	1	1	2		1	15	12/5/15
28	1	1	0		1	10	2/10/17
15	1	1	0		1	2	7/31/17
40	1	1	4		1	2	10/9/18
65	1	1	0		1	12	7/10/18
23	1	1	0		1	4	1/2/11
90	1	1	4		1	4	4/1/11
30	1	1	0		1	15	7/14/14
19	1	1	0		1	5	8/1/09
20	1	1	0		1	5	6/7/17
50	1	1	0		1	6	1/1/15
150	1	1	0		1	28	7/1/16
12	1	1	2		1	6	3/14/15
13	1	1	0		1	9	1/24/12
9	1	1	0		1	5	4/1/13
26	1	1	0		1	15	1/5/16
34.1	1	1	2		1	9	1/16/15

100	1	0					5/16/17
90	1	1	2		1	25	9/1/14
40	1	1	2		1	7	1/9/18
93	1	1	0		1	35	7/1/12
22	1	1	0		1	12	8/11/12
12.5	1	1	0		1	2	1/4/18
58	1	1	5	Novilin 70/3(	1	35	5/13/10
29	1	1	2		1	2	11/3/17
65	1	1	2		1	10	1/15/16
14	1	1	0		1	10	3/2/15
12.5	1	1	0		1	9	7/1/13
8	1	1	0		1	5	1/1/10
18	1	1	0		1	9	12/1/15
10	1	1	2		1	5	1/1/16
17	1	1	0		0	14	6/15/97
22	1	1	0		1	3	10/12/16
13	1	1	2		1	5	10/1/16
17	1	1	2		1	4	5/5/17
38	1	1	0		1	5	5/16/17
45	1	1	2		1	13	8/1/17
83	1	1	0		1	18	1/1/17
63.2	1	1	2		1	16	12/19/18
101	1	1	0		1	35	3/1/12
15	1	1	0		1	4	12/27/15
20	1	1	0		1	7	2/10/17
10	1	1	5	Versulin	1	3	1/20/11
75	1	1	0		1	10	11/1/17
125	1	1	0		1	30	1/11/17
24	1	1	2		1	10	10/10/16
13.2	1	1	0		1	3	7/1/17
10	1	1	0		1	4	2/1/13
26	1	1	0		1	4	11/1/12
22.2	1	1	2		1	6	12/29/17
22	1	1	2		1	3	4/15/16
60	1	1	0		1	18	11/15/16
24	1	1	4		1	5	4/1/12
17	1	1	0		1	8	4/16/11
50	1	1	0		1	5	9/1/16
12	1	1	0		1	6	4/1/10
64.5	1	1	2		1	17	11/17/17
93	1	1	0		1	16	2/23/17
17	1	1	5	Tresiba	0	7	3/23/16
13	1	1	0		1	6	3/5/13
28	1	1	0		1	6	5/9/12
15	1	1	2		1	4	10/26/17

40	1	1	0		1	8	12/8/18
21	1	1	0		1	6	3/1/12
17	1	1	0		1	8	4/2/13
19	1	1	0		1	11	2/15/13
15	1	1	0		1	6	4/1/13
14	1	1	0		1	4	2/5/10
16	1	1	0		1	8	4/1/17
	1	1	0		1	10	8/2/13
15	1	1	0		1	3	2/1/16
19	1	1	0		1	11	5/20/15
55	1	1	2		1	18	5/30/15
32	1	1	0		1	11	10/1/13
43	1	1	2		1	3	10/5/16
80	1	1	0		1	12	2/21/17
105	1	1	4		1	13	2/9/16
17	1	1	0		1	3	6/15/15
24	1	1	2		1	8	11/6/17
14.78	1	1	0		1	2	8/31/16
	1	1	5	caninsulin	1	4	6/6/12
14	1	1	0		1	8	1/1/17
65	1	1	0		1	19	4/12/16
19.5	1	1	0		1	4	3/10/13
16.7	1	1	0		1	5	8/1/11
13	1	1	0		1	3	2/6/17
85	1	1	0		1	10	8/1/14
26	1	1	0		1	5	11/1/14
63	1	1	2		1	14	2/2/18
27	1	1	0		1	8	5/1/14
18	1	1	0		1	5	5/8/18
7.5	1	1	2		1	3	2/2/18
36	1	1	2		1	7	1/16/19
32	1	1	0		1	8	9/14/16
25	1	1	0		1	8	6/1/16
60	1	1	2		1	16	3/18/15
77	1	1	5	Humulin 70/	1	22	10/17/16
31	1	1	0		1	9	3/20/15
14	1	1	0		1	6	10/1/09
10	1	1	5	Tresiba and I	0	17	1/13/16
80	1	0					1/1/15
65	1	1	0		1	9	9/1/15
30	1	1	0		1	16	9/1/14
18	1	1	2		1	6	12/28/15
18	1	1	2		1	3	5/5/17
15	1	1	2		1	2	7/9/18
6	1	1	0		1	1	12/4/13

65	1	1	2	1	15	1/1/15
86	1	1	0	1	18	11/5/15
12	1	1	2	1	5	8/22/17
50	1	1	0	1	9	5/22/15
9.5	1	1	2	1	4	12/14/16
21	1	1	0	1	9	6/17/12
22	1	1	0	1	10	8/5/13
35	1	1	2	1	8	5/1/15
30	1	1	0	1	21	5/15/12
20	1	1	2	1	10	1/1/17
70	1	1	2	1	15	4/3/17
30	1	1	0	1	6	1/15/15
16	1	1	2	1	4	6/15/17
72	1	1	2	1	32	12/10/16
21	1	1	2	1	6	7/24/17
15.5	1	1	0	1	4	3/10/14
11.6	1	1	0	1	5	6/1/12
56	1	1	0	1	5	2/16/15
25	1	1	0	1	7	2/3/16
17	1	1	4	1	2	5/17/12
80	1	1	2	1	25	1/1/13
66	1	1	2	1	10	4/28/17
19	1	1	0	1	4	6/23/18
10	1	1	0	1	3	3/2/17
	1	1	0	1	8	1/1/17
98	1	1	2	1	20	1/14/19
55	1	1	0	1	25	4/15/13
27	1	1	0	1	16	5/12/15
28	1	1	0	1	10	4/19/18
25	1	1	0	1	11	12/13/10
94	1	1	0	1	16	6/10/17
12	1	1	2	1	4	6/26/18
97	1	1	2	1	14	1/24/18
15	1	1	2	1	6	7/1/15
49	1	1	0	1	16	4/1/14
	1	1	0	1	6	6/1/09
28.5	1	1	2	1	2	12/12/19
	1	1	0	1	10	1/1/17
28	1	1	2	1	10	4/6/16
72.12	1	1	2	1	25	6/5/14
11	1	1	0	1	4	5/2/17
12.5	1	1	0	1	2	1/6/18
30	1	1	0	1	8	6/1/15
11	1	1	0	2	3	12/1/16
20	1	1	0	1	12	1/1/17

60	1	1	0		1	29	1/1/16
65	1	1	1		1	9	8/2/17
23	1	1	0		1	8	4/15/14
19	1	1	0		1	13	6/10/15
15	1	1	2		1	4	10/17/17
15	1	1	0		1	5	1/1/17
70	1	1	0		1	20	8/1/16
19	1	1	2		1	5	10/17/17
75	1	1	0		1	26	12/21/14
11	1	1	0		1	5	1/1/18
120	1	1	0		1	24	1/4/19
18	1	1	0		1		5/1/10
71	1	1	0		1	18	10/19/16
54	1	1	2		1	16	5/22/17
71	1	1	4		1	6	10/12/16
45	1	1	0		1	9	11/17/10
32	1	1	2		1	7	10/28/14
9	1	1	2		1	3	3/1/16
23	1	1	0		1	7	8/22/16
22	1	1	0		1	8	4/1/15
42	1	1	0		1	11	8/6/15
15	1	1	0		1	6	5/1/19
15	1	1	0		1	10	11/28/16
57.6	1	1	0		1	10	5/5/17
48	1	1	0		1	11	12/27/13
16	1	1	4		1	4	1/15/17
22	1	1	0		1	7	8/29/16
16	1	1	2		1	8	11/16/17
67	1	1	2		1	7	1/9/17
26	1	1	0		1	12	10/3/15
71	1	1	0		1	14	2/9/18
34	1	1	4		1	2	12/25/16
21	1	0					4/17/17
12	1	1	0		1	4	4/1/14
13	1	1	0		1	4	10/5/16
14	1	1	2		1	7	3/8/12
11.4	1	1	0		1	4	10/1/14
15	1	1	0		1	5	12/28/12
36	1	1	2		1	14	3/18/15
14.8	1	1	2		1	4	2/1/17
22	1	1	2		1	8	2/27/17
68	1	1	5	Novalin N	1	16	9/1/15
23	1	1	0		1	10	1/1/14
45	1	1	0		1	10	1/24/18
28	1	1	0		1	14	5/10/18

6.8	1	1	0		1	7	1/20/15
80	1	1	2		1	11	12/27/17
70	1	1	0		1	5	10/26/18
9	1	0					4/16/18
35	1	1	2		1	12	2/21/13
21	1	1	5	Caninsulin	1	6	1/3/18
20	1	1	5	Caninsulin	1	12	12/5/14
16	1	1	2		1	3	1/12/18
16	1	1	2		1	4	10/5/17
90	1	1	5	Caninsulin	1	17	12/16/17
16	1	1	0		1	6	1/6/13
9.5	1	1	0		1	2	4/21/17
14	1	1	2		1	6	3/1/15
9	1	0					5/19/18
75	1	1	0		1	12	10/15/18
14	1	1	0		1	5	1/1/14
80	1	1	0		1	28	11/27/13
22	1	1	2		0	9	12/4/18
16	1	1	0		1	7	8/23/12
14.1	1	1	2		1	4	3/1/14
9	1	1	2		1	4	7/20/16
92	1	1	2		1	26	4/3/15
13.8	1	1	0		1	8	11/8/14
16	1	1	0		1	4	10/12/10
14	1	1	0		1	6	1/15/17
70	1	1	0		1	22	1/1/17
74	1	1	0		1	19	4/16/16
18	1	1	2		1	4	3/2/17
14	1	1	2		1	4	1/11/18
80	1	0					6/1/16
20	1	1	2		1	12	4/16/17
90	1	1	4		1	8	3/9/18
18	1	1	0		1	2	10/1/09
	1	1	1		0	8	12/31/00
70	1	1	2		1	15	10/21/17
18	1	1	0		1	2	6/1/13
19.4	1	1	0		1	2	7/26/17
42	1	1	2		1	8	4/1/16
30	1	1	0		1	10	5/1/15
88	1	1	2		1	17	4/4/17
20	1	1	0		1	4	7/29/16
64	1	1	0		1	18	8/12/15
43	1	1	0		1	12	5/1/15
20	1	1	0		1	9	11/16/16
20	1	1	0		1	11	1/1/17

10	1	0					2/14/14
60	1	0					1/1/15
18	1	1	2		1	5	3/18/17
22	1	1	0		1	10	4/1/17
12.6	1	1	2		1	5	6/19/14
54	1	1	0		1	35	6/13/09
9	1	1	2		1	3	11/1/17
15	1	1	0		1	4	1/1/12
75	1	1	0		1	35	8/16/16
5.8	1	1	2		1	2	6/9/17
32	1	1	0		1	5	5/18/17
60	1	1	0		1	11	6/1/17
90	1	1	4		1	4	10/26/18
45	1	1	2		1	8	2/5/19
29	1	1	0		1	8	1/6/19
25	1	1	0		1	5	8/28/15
52	1	1	0		2	20	11/15/16
86	1	1	0		1	16	3/25/15
65	1	1	0		1	16	1/10/13
22	1	1	0		1	9	6/1/16
26	1	1	2		1	3	1/2/19
21	1	1	0		1	4	7/19/17
12	1	1	5	Caninsulin	1	8	1/5/17
8	1	1	2		1	4	11/27/17
23.6	1	1	2		1	4	2/7/18
70	1	1	0		1	20	12/30/15
16	1	1	5	Novolin 70/3	1	8	7/1/17
50	1	1	2		1	6	7/12/18
10	1	1	2		1	5	7/10/18
20	1	1	5	Caninsulin	1	3	1/9/18
28	1	1	0		1	14	8/1/16
52	1	1	0		1	16	4/11/10
15	1	1	0		1	5	7/23/14
70	1	1	4		1	10	9/1/14
20	1	1	0		1	6	12/5/16
80	1	1	0		1	20	4/1/16
10	1	1	2		1	2	4/6/17
18	1	1	0		1	5	3/22/18
87	1	1	0		1	17	11/1/15
12	1	1	2		1	4	7/15/16
108	1	1	0		2	8	9/15/15
19.2	1	1	2		1	9	7/30/18
25	1	1	0		1	9	5/1/16
22	1	1	0		1	13	12/30/16
15.9	1	1	2		1	8	2/9/17

11.4	1	1	5	Vetsulin	1	3	12/12/12
25	1	1	0		1	9	5/30/07
26	1	1	0		1	7	7/4/15
25	1	1					5/22/17
9.5	1	1	2		1	3	3/15/15
70	1	1	0		1	20	1/15/17
65	1	1	2		1	8	8/5/17
13	1	1	2		1	5	10/10/14
15.5	1	1	0		1	4	8/25/16
64	1	1	2		1	16	3/1/17
45	1	1	0		1	11	10/1/16
13	1	1	2		1	6	11/4/13
55	1	1	0		1	15	6/6/18
38	1	1	2		1	7	9/14/18
19	1	1	0		1	5	3/15/15
65	1	1	5	Caninsulin	1	6	3/27/15
15	1	1	2		1	2	2/7/18
13	1	1	0		1	6	9/11/16
15	1	1	2		1	5	2/27/17
11	1	1				3	1/1/15
20	1	1	2		1	4	4/10/18
17	1	1	0		1	6	9/1/14
12	1	1	0		1	6	3/1/17
29	1	1	2		1	10	8/10/16
65	1	1	0		1	9	6/27/14
25	1	1	2		1	12	12/6/17
19	1	1	0		1	5	1/20/17
27	1	1	2		1	6	4/1/17
7	1	1	2		1	5	9/19/16
20	1	1	0		1	9	5/10/17
28	1	1	0		1	9	5/5/16
25	1	1	0		1	13	5/3/16
19	1	1	0		1	8	3/1/17
27	1	1	0		1	15	9/1/15
28	1	1	0		1	5	9/19/18
30	1	1	2		1	7	11/21/17
9.6	1	1	0		1	5	1/3/14
20	1	1	2		1	7	4/13/15
29	1	1	0		1	10	1/21/17
18	1	1	2		1	8	8/24/16
10.5	1	1	0		1	3	7/29/17
52	1	1	2		1	15	7/1/15
23	1	1	2		1	8	9/10/18
74.4	1	1	0		1	25	5/27/17
31	1	1	0		1	6	2/25/16

70	1	1	2		1	13	10/19/18
20	1	1	2		1	4	1/8/17
44	1	1	2		1	11	2/20/14
78.5	1	1	2		1	30	2/21/17
11	1	1	0		1	8	12/26/18
11	1	1	0		1	5	2/7/14
12	1	1	2		1	8	4/18/17
16	1	1	4		1	3	12/1/16
11	1	1	5		1	2	12/12/17
52	1	1	2		1	9	1/25/17
18	1	1	2		1	2	5/2/16
17	1	1	0		1	5	2/28/17
24	1	1	0		1	14	2/2/15
36.5	1	1	0		1	14	1/20/17
73	1	1	0		1	11	7/6/15
14	1	1	0		1	12	4/1/15
22.3	1	1	2		1	7	11/1/17
19	1	1	0		1	6	8/22/17
75	1	1	0		1	27	12/10/16
22	1	1	2		1	6	11/4/17
15	1	1	0		1	5	6/20/15
16	1	1	2		1	4	3/1/16
15	1	1	2		1	7	2/1/16
31	1	1	0		1	13	3/1/16
12	1	1	5	Caninsulin	1	4	6/26/15
11	1	1	2		1	2	11/1/16
15	1	0					12/1/16
65	1	1	0		1	6	5/8/18
15	1	1	2		1	5	3/15/17
50	1	1	2		1	13	1/11/18
12.8	1	1	2		1	4	1/9/17
46	1	1	2		1	10	6/10/18
75	1	1	0		1	16	11/16/16
18	1	1	2		1	10	3/20/16
77.8	1	1	0		1	18	8/9/16
25	1	1	0		1	15	1/8/18
68	1	1	0		1	14	5/7/17
25	1	1	0		1	9	6/14/14
12.5	1	1	0		1	8	9/25/14
20.8	1	1	0		1	9	9/1/13
49	1	1	2		1	11	11/24/17
60	1	1	0		1	24	2/18/17
38	1	1	2		1	24	3/1/16
39	1	1	2		1	13	1/1/16
16	1	1	0		1	7	1/1/12

22	1	1	0		1	8	12/28/15
20	1	1	2		1	1	11/26/13
28	1	1	2		1	6	12/1/18
22	1	1	0		1	6	4/21/17
14.5	1	1	2		0	5	7/2/19
23	1	1	2		1	4	5/22/15
17	1	1	0		1	8	7/19/16
12.6	1	1	2		1	3	6/22/17
85	1	1	0		1	26	7/9/18
36	1	1	0		1	12	6/5/16
38	1	0					8/19/16
85	1	1	0		1	2	3/1/17
7	1	1	0		1	2	7/4/14
28	1	1	0		1	7	8/6/16
22	1	1	0		1	8	4/26/16
17	1	1	2		1	3	10/22/17
11	1	0					9/12/16
120	1	1	0		1	28	3/1/17
20	1	1	0		1	5	11/8/17
95	1	1	0		1	16	4/5/18
19	1	1	0		1	1	1/10/19
33	1	1	2		1	5	2/6/19
86	1	0					7/11/16
18	1	1	2		1	4	5/12/17
23	1	1	0		1	5	12/23/16
65	1	1	0		1	19	10/1/17
16	1	1	0		1	6	5/15/14
33.2	1	1	0		1	10	3/27/19
15	1	1	2		1	6	10/1/15
24	1	1	2		1	4	1/25/18
80	1	0					8/12/17
30	1	1	5	Noviln n	1	10	9/15/15
63	1	1	2		1	27	2/16/17
42	1	1	0		1	14	6/18/15
27	1	1	0		1	10	6/4/17
22	1	1	0		1	5	3/14/16
12.8	1	1	0		1	4	11/1/15
17.5	1	1	2		1	5	5/1/17
	1	1	2		1	3	8/16/17
28	1	1	2		1	6	10/27/17
25	1	1	2		1	5	12/23/18
25	1	1	0		1	9	7/1/15
9.4	1	1	0		1	6	1/1/17
14	1	1	0		1	3	12/23/14
33	1	1	0		1	12	2/6/17

48	1	1	0		1	6	8/16/18
30	1	1	2		1	5	1/23/15
18	1	1	0		1	8	11/6/15
17	1	1	0		1	11	8/1/17
10	1	1	0		1	1	12/2/17
18	1	1	0		1	8	1/1/17
80	1	1	2		1	24	11/22/17
50	1	1	0		1	15	4/30/19
	1	1	1		1	16	10/23/17
65	1	1	0		1	25	2/22/17
60	1	1	5	Don't know j	1	14	7/7/17
17.9	1	1	0		1	7	4/18/14
51	1	1	0		1	16	10/1/16
16	1	1	0		1	6	2/10/15
119.8	1	1	2		1	12	11/30/17
33	1	1	0		1	5	11/3/18
70	1	1	0		1	24	11/28/16
68	1	1	2		1	14	7/2/15
53	1	1	2		1	15	2/2/18
16	1	1	4		1	1	11/1/09
53	1	1	2		1	12	1/1/17
29	1	0					1/4/18
42	1	1	0		1	14	8/16/16
22.4	1	1	2		1	12	9/6/16
20	1	1	0		1	6	7/1/15
9	1	1	0		1	3	10/24/17
12	1	1	0		1	6	6/15/18
14.3	1	1	0		1	7	7/30/14
18	1	1	0		1	4	3/9/16
16	1	1	2		1	6	12/1/14
28	1	0					3/30/16
12	1	1	0		1	5	2/7/18
13.4	1	1	2		1	6	2/13/19
15	1	1	0		1	5	12/9/16
11	1	1	0		1	3	11/22/15
40	1	1	2		1	9	1/9/17
50	1	1	2		1	12	1/15/16
12	1	1	0		1	2	1/26/18
65	1	1	5	Caninsulin	1	10	6/1/17
16	1	1	1		1	2	1/20/15
25	1	1	0		1	15	8/1/16
14	1	1	0		1	4	3/20/17
87	1	1	0		1	23	1/1/18
17	1	1	0		1	5	4/1/14
24	1	1	0		1	8	10/1/15

62	1	1	2		1	14	2/16/17
32	1	1	0		1	11	8/1/16
15	1	1	2		1	4	1/1/17
27	1	1	2		1	7	12/7/17
15	1	1	0		1	6	7/21/16
20	1	1	0		1	7	12/15/15
22	1	1	5	Caninsulin	1	20	8/1/12
13	1	1	1		1	3	9/25/14
75	1	1	0		1	20	11/15/16
10	1	1	2		1	4	4/17/19
11	1	1	0		1	4	2/1/17
20	1	1	0		1		3/5/13
10	1	1	0		1	3	10/15/14
61	1	1	2		1	12	2/27/18
8.8	1	1	0		1	2	6/26/17
32	1	1	2		1	8	8/12/17
40	1	1	0		0		7/1/79
42	1	1	2		1	12	10/1/18
14	1	1	0		1	7	4/12/01
11	1	1	0		1	2	4/11/17
19.5	1	1	2		1	12	2/1/14
14.17	1	1	5	toujeo	1	3	6/6/19
17	1	0					6/6/13
75	1	1	0		1	17	2/13/18
13	1	1	0		1	1	10/10/17
10	1	1	2		1	3	5/5/16
31	1	1	2		1	13	2/3/13
13	1	1	2		1	5	1/1/17
40	1	0					1/1/17
20	1	1	0		1	5	8/15/16
55	1	1	2		0		2/4/13
25	1	1	0		1	11	12/20/15
16	1	1	0		1	4	4/5/17
25.4	1	1	0		1	7	4/25/17
32	1	1	0		1	12	12/23/17
83	1	1	0		1	12	5/15/17
38	1	1	0		1	5	2/1/18
70	1	1	0		1	15	1/10/17
16	1	1	2		1	5	3/24/18
13	1	1	5	Novolin	1	9	11/15/17
96	1	1	0		1		9/1/17
22	1	1	0		1	12	6/9/14
75	1	1	0		1	6	3/1/13
89	1	1	0		1	26	4/15/16
40	1	0					5/1/16

25	1	1	2	1	9	8/17/14
17	1	1	0	1	5	10/6/18
11.4	1	1	0	1	2	6/30/18
19	1	1	0	1	9	2/1/18
38	1	1	0	1	7	6/24/18
55	1	1	2	1	6	6/12/16
51	1	1	2	1	16	10/15/15
19.5	1	1	0	1	8	8/1/17
16	1	1	0	1	5	10/1/13
42	1	1	0	1	5	5/10/12
13	1	1	4	1	3	12/16/16
10	1	1	0	1	4	9/4/17
22	1	1	0	1	8	10/26/17
22	1	1	2	1	9	6/1/16
11	1	1	0	1	3	10/20/17
22	1	1	2	1	3	11/1/17
24.2	1	1	2	1	4	11/4/19
48	1	1	0	1	7	5/1/17
20	1	1	2	1	5	10/11/18
11	1	1	0	1	2	12/1/15
11.5	1	1	0	1	3	11/15/18
30	1	1	2	1	4	6/28/18
11	1	1	2	1	4	12/20/17
55	1	1	0	1	22	5/22/17
55	1	1	0	1	11	1/15/19
17.4	1	1	0	1	9	10/1/15
20	1	1	0	1	12	1/15/15
62	1	1	0	1	15	6/1/16
27	1	1	2	1	8	10/4/16
11.3	1	1	0	1	3	5/21/18
92	1	1	0	2	12	12/5/15
16	1	1	1	1	6	6/7/17
45	1	1	0	1	11	11/1/17
17.4	1	1	0	1	6	1/2/14
13.5	1	1	0	1	3	4/8/17
65	1	1	0	1	15	3/1/17
17	1	1	2	1	10	11/26/17
12	1	1	0	1	7	5/2/17
15	1	1	2	1	2	12/1/17
14	1	1	0	1	6	6/1/16
70	1	1	0	1	7	9/15/10
38	1	1	0	1	6	10/1/16
64	1	1	0	1	17	7/13/17
34	1	1	0	1	10	6/15/16
28	1	1	0	1	7	4/23/18

16	1	1	0		1	5	1/15/17
80	1	1	0		1	24	11/15/16
17	1	1	2		1	3	4/7/17
20	1	1	5	Caninsulin	1	11	7/31/16
15.8	1	1	0		1	5	5/26/17
12	1	1	2		0	7	1/1/17
53	1	1	2		1	13	9/1/14
13	1	1	0		1	5	7/9/17
8.6	1	1	2		1	2	1/1/17
	1	1	4		1	4	12/1/16
54	1	1	0		1	12	4/1/15
16	1	1	2		1	3	7/12/17
20	1	1	0		1	3	11/5/17
25	1	1	2		1	9	8/3/16
61	1	1	2		1	15	2/4/19
24	1	0					2/5/19
26	1	1	4		1	2	1/26/18
14	1	1	2		1	4	2/26/19
29	1	1	0		1	5	1/1/15
18	1	1	0		1	6	10/22/18
20	1	1	0		1	15	12/26/14
20	1	0					9/16/16
10	1	1	0		1	5	12/1/16
60	1	1	5	Caninuslin	1	7	6/16/17
20	1	1	0		1	7	1/1/17
23	1	1	0		1	11	1/1/17
16	1	1	0		1	6	12/1/16
26	1	1	0		1	6	12/21/17
56	1	1	2		1	12	6/5/17
52	1	1	0		1	8	1/4/18
25	1	1	0		1	12	10/1/16
55	1	1	0		1	8	9/1/18
17.8	1	1	0		1	5	4/29/16
14.3	1	1	2		1	4	10/18/16
16.8	1	1	0		1	5	3/18/17
25	1	1	0		1	6	1/1/17
30	1	0					6/15/16
9	1	1	2		1	2	3/22/17
17	1	1	0		1	6	8/1/19
48	1	1	2		1	16	10/10/18
15	1	1	2		1	4	4/1/14
27	1	1	0		1	11	11/1/17
7	1	1	1		1	2	1/10/17
13	1	1	0		1	6	8/1/18
24	1	1	2		1	5	1/3/18

	1	1	5	Caninsulin	1	13	1/9/18
9	1	1	2		1	3	4/20/16
	1	1	0		1	2	2/24/18
20	1	1	0		1	9	7/4/17
24	1	1	0		1	8	6/8/17
15	1	1	0		1	6	10/30/16
	1	1	0		1	6	6/4/18
24.4	1	1	2		1	7	4/29/19
19.6	1	1	0		1	8	5/28/17
12	1	1	0		1	4	6/15/15
130	1	1	0		1	15	9/1/16
55	1	1	2		1	13	3/16/16
19	1	1	2		1	2	10/20/17
33	1	1	0		1	2	7/1/12
80	1	1	1		1		6/15/16
55	1	1	0		1	13	3/29/17
19.5	1	1	0		1	2	8/15/17
95	1	1			1	15	6/16/17
26	1	1	2		1	3	4/6/18
12	1	1	0		1	6	3/9/17
26	1	1	0		1	8	9/15/17
21.6	1	1	0		1	6	8/19/15
20	1	1	1		1	5	6/1/17
60	1	1	0		1	18	10/24/16
12	1	1	2		1	6	1/11/18
65	1	0					8/1/14
9	1	1	2		1	4	4/8/19
22	1	0					4/10/14
78	1	1	0		1	15	5/31/18
10	1	1	2		1	4	10/15/16
90	1	1	0		1	32	5/5/16
	1	1	0		1	4	3/30/17
32.5	1	1	0		1	7	7/15/16
44	1	1	0		1	16	11/24/17
11	1	1	0		1	5	6/5/18
25	1	1	2		1	7	1/1/16
18	1	0					9/5/19
35	1	1	0		1	4	5/25/18
55	1	1	2		1	12	1/20/17
28	1	1	0		1	13	1/21/16
22	1	1	0		1	4	1/1/17
22	1	1	0		1	9	3/1/17
18	1	1	0		1	4	8/19/17
18	1	1	0		1	10	7/8/15
18	1	1	0		1	4	8/24/17

12.6	1	1	0		1	5	1/23/15
12	1	1	2		1	3	1/11/17
20	1	1	0		1	5	11/14/17
30	1	1	0		1	25	2/13/17
35	1	1	0		1	12	3/8/17
12	1	1	5	Novolin N	1	5	5/1/18
52	1	1	0		1	14	2/12/17
24	1	1	2		1	9	6/5/17
39.2	1	1	0		1	9	2/12/16
9	1	0					6/1/14
50	1	1	0		1	13	3/1/16
11	1	1	0		1	8	1/1/15
12	1	1	2		1	4	5/1/16
29	1	1	2		1	12	12/1/16
20	1	1	0		1	9	3/15/17
45.1	1	1	2		1	10	8/28/17
27	1	1	2		1	9	2/18/17
40	1	1	0		1	13	10/4/14
58	1	1	0		1	18	4/1/19
48.8	1	1	0		1	22	6/1/15
55	1	1	2		1	7	5/17/17
23	1	1	0		1	5	6/11/17
25	1	1	2		1	9	6/16/17
11	1	1	0		1	3	1/30/15
70	1	1	2		1	32	3/10/16
7	1	1	0		1	1	1/1/17
16	1	1	0		1	5	2/6/17
20	1	1	2		1	8	3/21/17
10	1	1	0		1	3	6/29/18
6	1	1	0		1	1	3/18/19
32	1	1	2		1	7	6/1/17
99	1	1	0		1	32	2/2/17
22	1	1	0		1	6	6/11/18
	1	1	2		1	12	6/21/17
13	1	1	2		1	5	5/17/17
25	1	1	0		1	8	11/4/16
26	1	1	2		1	9	11/1/14
11	1	1	0		1	5	4/20/15
9	1	1	2		1	4	9/10/18
	1	1	0		1	11	6/15/15
25	1	1	0		1	5	1/22/18
28	1	1	0		1	5	2/1/19
37	1	1	2		1	9	5/5/17
22	1	1	0		1	5	12/21/17
15	1	1	0		1	6	1/1/17

15	1	1	2		1	6	2/17/18
25	1	1	5	Cant rememl	1		7/20/07
9	1	1	0		1	4	11/1/16
70	1	0					1/2/18
74	1	1	2		1	16	1/12/18
8	1	0					7/15/14
65	1	0					1/31/16
53	1	1	0		1	15	5/30/17
27	1	1	2		1	12	2/7/17
38	1	1	0		1	9	7/26/18
9.75	1	1	0		1	5	8/14/17
70	1	1	0		1	12	11/30/15
13.2	1	1	0		1	2	12/6/16
18	1	1	2		1	10	11/1/16
24	1	1	0		1	6	8/24/16
76	1	1	0		1	7	3/7/19
25	1	1	0		1	5	8/24/18
80	1	1	0		1	14	8/1/17
15	1	1	0		1	7	1/11/17
18	1	1	0		1	5	3/16/17
25	1	1	0		1	10	11/3/16
45	1	1	2		1	30	6/12/19
46	1	1	0		1	20	6/12/17
20	1	1	2		1	5	4/25/17
22	1	1	2		1	8	3/31/16
25	1	1	2		1	4	8/9/17
14	1	1	2		1	15	6/11/18
29	1	1	2		1	5	2/10/18
19	1	1	0		1	7	2/28/19
16	1	1	0		1	10	12/1/15
75	1	1	0		1	20	1/13/18
19	1	1	0		1	2	1/1/17
80	1	0					5/1/17
54	1	1	5	Caninsulin	1	10	6/30/17
23	1	1	0		1	10	11/30/18
67	1	1	2		1	23	2/1/16
11.8	1	1	2		1	2	5/21/18
38	1	1	0		1	7	3/3/17
20	1	1	1		1	7	4/5/15
20	1	1	0		1	7	2/1/16
40	1	1	0		1	8	11/20/18
19	1	1	2		1	5	3/22/17
18	1	1	2		1	6	1/11/17
19	1	0					10/18/18
52	1	1	0		1		3/15/18

45	1	1	0		1	12	4/4/19
14	1	1	2		1	7	12/25/16
9	1	1	2		1	2	6/8/18
62	1	1	2		0	18	3/17/18
24	1	1	0		1	13	5/17/17
9	1	0					11/10/17
12	1	1	5	Cannulin	1	4	4/3/17
12	1	1	0		1	6	6/1/17
16	1	1	2		1	5	4/15/17
17	1	1	2		1	3	12/24/14
60	1	1	0		1	6	1/29/18
82.4	1	1	0		1	20	5/23/19
20	1	1	4		1	1	4/4/14
14	1	1	2		1	5	12/10/17
11	1	1	0		1	2	11/1/15
20	1	1	2		1	8	9/28/17
17	1	1	2		1	9	1/1/17
40	1	1	0		1	16	1/20/16
40	1	1	2		1	10	9/26/17
43	1	1	0		1	12	2/20/17
85	1	1	0		1	12	3/1/15
32	1	1	0		1	7	7/20/17
8	1	1	0		1	2	6/20/17
67	1	0					4/1/16
24	1	1	2		1	5	1/1/17
12	1	1	2		1	3	8/23/17
45	1	1	1		1	7	6/1/17
15.6	1	0					1/14/18
17	1	0					4/6/19
17	1	1	0		1	6	11/1/16
6	1	1	0		1	5	8/2/19
24	1	1	2		1	9	5/20/08
73	1	1	2		1	35	5/12/16
41	1	1	0		1	18	3/15/17
70	1	1	0		1	20	2/1/17
55	1	1	2		0	12	12/28/19
15	1	1	0		1	5	1/7/16
	1	1	0		1	2	9/20/17
11	1	1	0		0	2	10/24/17
11	1	1	5		1	3	3/15/19
64	1	1	2		1	17	9/1/15
85	1	1	0		1	23	1/1/12
32.5	1	1	2		1	8	10/14/16
18.2	1	1	2		1	6	2/15/15
26	1	1	0		1	7	.

70	1	1	0		1	20		4/3/17
11.5	1	1	5	Caninsulin	1	7	.	
11	1	1	0		1	5		4/11/17
15	1	1	2		1	2	.	
12	1	1	0		1	5		6/1/09
35	1	1	2		1		.	
	1	0					.	
13.5	1	1	0		1	6		6/1/16
12	1	1	5	Vetmedin	1	5	.	
53	1	0					.	
95	1	1	0		1	13		6/1/15
20	1	1	0		1	13		9/20/14
30	1	1	2		1	6		8/20/08
12	1	1	2		1	9	.	
8	1	0					.	
21	1	1	0		1	15	.	
11	1	1	0		1	5	.	
21	1	1	2		1	5	.	
135	1	0					.	
90	1	0					.	
12	1	1	0		1	3		9/21/17
48	1	1	2		1	8		10/26/17
73	1	0					.	
20	1	1	2		1	6		7/1/00
18	1	1	0		1	34		11/18/14
38	1	1	0				.	
31	1	0						1/1/03
40	1	1					.	
70	1	1	0		1	10	.	
24	1	1	0		1	2	.	
118	1	1	2		1	10		1/2/17
20	1	1	0		1	4		1/13/18
24	1	1	0		1	7		4/19/17
	1	1	4		1	2	.	
15.5	1	1	0		1	5	.	
	1	1	5	Caninsulin	1	12		2/7/18
22	1	1	5	Caninsulin	1	7		1/6/16
30.6	1	1	0		1	4	.	
	1	1	2			13		8/24/17
86	1	1	0		1	35		9/15/17
23.6	1	1	0		1	7		2/14/14
60	1	1	0		1	16		12/8/16
15	1	1	0		1	7		1/2/15
105	1	1	0		1	21		11/15/17
17	1	1	2		1	7		2/28/16

18.6	1	1	2		1	5		1/15/18
	1	0					.	
32	1	0						3/15/14
50	1	1					.	
22	1	1	2		1			1/10/08
95	1	1	2		1	18	.	
37	1	1	5	caninsulin	1	4		2/16/12
14.5	1	1	1		1	3		6/11/18
12.4	1	1	2		1	5		8/8/18
21	1	1	2		1	7	.	
16	1	1	2		1	8	.	
21	1	1	2		1	5		2/27/19
10	1	1	0		1	5	.	
21	1	1	0		2	11		10/1/13
22	1	1	0		1	7	.	
	1	1	0		1	9		3/3/15

Age at DM Dx	Month of DMDx
3.00	6.00
5.00	11.00
8.00	1.00
16.00	8.00
38.00	10.00
45.00	6.00
58.00	10.00
62.00	6.00
73.00	
74.00	12.00
75.00	5.00
77.00	7.00
86.00	5.00
88.00	5.00
94.00	
96.00	
105.00	8.00
158.00	12.00
158.00	
159.00	8.00
182.00	
207.00	
216.00	
232.00	1.00
241.00	
267.00	10.00
267.00	12.00
365.00	
440.00	
443.00	8.00
485.00	4.00
502.00	9.00
507.00	10.00
513.00	5.00
604.00	
664.00	1.00
686.00	2.00
715.00	
720.00	5.00
730.00	1.00
783.00	5.00
839.00	9.00
851.00	
856.00	9.00

882.00	12.00
892.00	7.00
911.00	9.00
945.00	1.00
968.00	1.00
972.00	5.00
980.00	
1006.00	6.00
1006.00	
1042.00	
1075.00	
1078.00	1.00
1096.00	9.00
1113.00	5.00
1113.00	12.00
1124.00	
1128.00	7.00
1141.00	1.00
1147.00	10.00
1157.00	6.00
1198.00	4.00
1199.00	7.00
1205.00	9.00
1208.00	7.00
1212.00	1.00
1212.00	1.00
1214.00	2.00
1220.00	
1228.00	7.00
1230.00	10.00
1242.00	1.00
1244.00	10.00
1248.00	8.00
1278.00	1.00
1304.00	12.00
1311.00	4.00
1359.00	12.00
1366.00	5.00
1367.00	4.00
1371.00	1.00
1383.00	5.00
1397.00	
1400.00	1.00
1416.00	1.00
1427.00	

1433.00	5.00
1445.00	10.00
1457.00	5.00
1491.00	5.00
1517.00	12.00
1522.00	
1523.00	
1524.00	6.00
1531.00	
1551.00	3.00
1558.00	8.00
1575.00	9.00
1590.00	
1601.00	
1608.00	
1612.00	5.00
1614.00	
1637.00	
1672.00	
1687.00	11.00
1700.00	
1715.00	6.00
1715.00	
1724.00	4.00
1734.00	4.00
1737.00	11.00
1739.00	6.00
1746.00	4.00
1747.00	
1749.00	2.00
1759.00	7.00
1765.00	
1773.00	
1791.00	1.00
1812.00	4.00
1816.00	7.00
1826.00	8.00
1826.00	6.00
1826.00	1.00
1827.00	7.00
1839.00	3.00
1840.00	1.00
1857.00	4.00
1857.00	1.00
1862.00	1.00

1865.00	5.00
1874.00	9.00
1881.00	1.00
1884.00	7.00
1884.00	
1910.00	
1914.00	5.00
1915.00	
1926.00	1.00
1928.00	3.00
1939.00	7.00
1955.00	1.00
1969.00	12.00
1989.00	1.00
2003.00	6.00
2007.00	10.00
2010.00	
2011.00	5.00
2024.00	5.00
2027.00	8.00
2038.00	1.00
2046.00	
2054.00	
2061.00	12.00
2070.00	2.00
2071.00	1.00
2088.00	11.00
2097.00	1.00
2099.00	10.00
2124.00	7.00
2129.00	2.00
2131.00	11.00
2133.00	
2138.00	4.00
2139.00	11.00
2146.00	4.00
2150.00	4.00
2153.00	9.00
2162.00	4.00
2164.00	
2167.00	2.00
2173.00	
2174.00	3.00
2177.00	5.00
2185.00	10.00

2187.00	
2191.00	3.00
2192.00	4.00
2192.00	2.00
2192.00	4.00
2192.00	2.00
2192.00	
2196.00	8.00
2198.00	2.00
2210.00	
2211.00	5.00
2213.00	10.00
2220.00	10.00
2223.00	2.00
2223.00	
2224.00	
2229.00	11.00
2243.00	8.00
2246.00	
2248.00	
2253.00	4.00
2260.00	
2265.00	8.00
2268.00	2.00
2269.00	8.00
2276.00	11.00
2285.00	2.00
2288.00	5.00
2296.00	
2297.00	
2315.00	
2317.00	9.00
2317.00	6.00
2324.00	3.00
2328.00	10.00
2329.00	3.00
2329.00	
2333.00	1.00
2334.00	1.00
2342.00	9.00
2344.00	9.00
2352.00	12.00
2354.00	5.00
2370.00	
2374.00	12.00

2377.00	1.00
2379.00	11.00
2381.00	8.00
2381.00	5.00
2388.00	12.00
2390.00	
2391.00	8.00
2414.00	5.00
2418.00	5.00
2418.00	1.00
2428.00	4.00
2437.00	1.00
2440.00	6.00
2447.00	
2462.00	7.00
2463.00	3.00
2470.00	6.00
2482.00	
2483.00	2.00
2483.00	
2485.00	1.00
2490.00	4.00
2491.00	
2496.00	3.00
2498.00	1.00
2504.00	
2510.00	4.00
2514.00	5.00
2514.00	
2516.00	12.00
2520.00	6.00
2521.00	
2522.00	1.00
2525.00	7.00
2527.00	4.00
2532.00	6.00
2536.00	
2542.00	1.00
2543.00	
2546.00	6.00
2549.00	
2553.00	1.00
2556.00	6.00
2557.00	12.00
2557.00	

2558.00	1.00
2564.00	
2568.00	4.00
2570.00	6.00
2577.00	10.00
2590.00	1.00
2600.00	8.00
2613.00	10.00
2622.00	12.00
2625.00	1.00
2625.00	
2635.00	5.00
2636.00	
2646.00	5.00
2647.00	10.00
2653.00	11.00
2660.00	10.00
2663.00	3.00
2663.00	8.00
2671.00	4.00
2674.00	8.00
2677.00	
2679.00	11.00
2682.00	5.00
2684.00	12.00
2685.00	1.00
2693.00	8.00
2693.00	
2695.00	1.00
2696.00	10.00
2699.00	
2704.00	12.00
2708.00	4.00
2728.00	4.00
2735.00	10.00
2735.00	
2740.00	10.00
2748.00	12.00
2749.00	3.00
2750.00	2.00
2753.00	2.00
2756.00	9.00
2760.00	1.00
2763.00	1.00
2769.00	

2770.00	
2773.00	12.00
2778.00	
2781.00	
2782.00	
2783.00	1.00
2788.00	12.00
2789.00	1.00
2798.00	10.00
2802.00	
2803.00	1.00
2810.00	4.00
2817.00	3.00
2819.00	
2822.00	
2823.00	1.00
2824.00	11.00
2826.00	
2828.00	8.00
2830.00	3.00
2834.00	7.00
2834.00	4.00
2835.00	11.00
2835.00	10.00
2837.00	1.00
2844.00	1.00
2845.00	4.00
2847.00	3.00
2847.00	
2851.00	6.00
2852.00	
2853.00	
2859.00	10.00
2859.00	12.00
2871.00	10.00
2875.00	6.00
2875.00	7.00
2884.00	4.00
2891.00	
2894.00	4.00
2897.00	7.00
2902.00	8.00
2906.00	5.00
2907.00	11.00
2915.00	

2919.00	2.00
2922.00	1.00
2922.00	3.00
2922.00	4.00
2922.00	6.00
2922.00	6.00
2922.00	11.00
2922.00	1.00
2926.00	8.00
2926.00	6.00
2929.00	5.00
2929.00	6.00
2938.00	
2941.00	
2947.00	
2949.00	8.00
2950.00	11.00
2954.00	3.00
2958.00	1.00
2959.00	
2962.00	
2965.00	7.00
2970.00	1.00
2972.00	
2981.00	2.00
2983.00	12.00
2983.00	7.00
2983.00	
2985.00	
2988.00	1.00
2990.00	8.00
2991.00	4.00
2996.00	7.00
3004.00	9.00
3008.00	12.00
3009.00	
3017.00	
3024.00	
3025.00	11.00
3027.00	7.00
3028.00	
3031.00	
3033.00	5.00
3033.00	
3038.00	2.00

3040.00	12.00
3041.00	5.00
3042.00	7.00
3043.00	5.00
3049.00	3.00
3051.00	1.00
3057.00	8.00
3066.00	10.00
3068.00	8.00
3068.00	3.00
3075.00	10.00
3075.00	11.00
3078.00	
3084.00	
3084.00	
3090.00	
3092.00	2.00
3096.00	9.00
3099.00	
3103.00	1.00
3105.00	
3106.00	9.00
3108.00	
3109.00	8.00
3110.00	6.00
3111.00	
3112.00	1.00
3115.00	4.00
3115.00	9.00
3124.00	5.00
3126.00	
3128.00	5.00
3128.00	
3133.00	9.00
3136.00	
3138.00	
3139.00	1.00
3143.00	
3144.00	1.00
3148.00	8.00
3155.00	
3164.00	7.00
3167.00	
3168.00	5.00
3172.00	2.00

3176.00	
3177.00	1.00
3178.00	2.00
3186.00	2.00
3188.00	
3189.00	2.00
3196.00	4.00
3198.00	12.00
3200.00	12.00
3219.00	1.00
3222.00	5.00
3223.00	2.00
3225.00	2.00
3225.00	1.00
3226.00	7.00
3226.00	4.00
3226.00	
3233.00	8.00
3240.00	12.00
3242.00	
3245.00	6.00
3253.00	
3255.00	2.00
3255.00	3.00
3256.00	6.00
3258.00	11.00
3260.00	12.00
3260.00	
3268.00	
3269.00	1.00
3271.00	1.00
3274.00	
3276.00	11.00
3276.00	3.00
3278.00	8.00
3281.00	
3282.00	5.00
3287.00	6.00
3287.00	9.00
3287.00	
3287.00	
3288.00	2.00
3288.00	3.00
3293.00	
3297.00	1.00

3303.00	12.00
3314.00	11.00
3323.00	
3327.00	4.00
3329.00	
3332.00	5.00
3332.00	7.00
3333.00	6.00
3333.00	
3338.00	6.00
3342.00	8.00
3343.00	3.00
3344.00	7.00
3349.00	8.00
3349.00	4.00
3350.00	10.00
3350.00	
3359.00	3.00
3359.00	
3365.00	
3365.00	
3365.00	
3367.00	7.00
3370.00	5.00
3371.00	12.00
3372.00	
3373.00	5.00
3378.00	
3381.00	10.00
3384.00	1.00
3390.00	8.00
3393.00	9.00
3393.00	
3394.00	6.00
3396.00	6.00
3398.00	3.00
3403.00	11.00
3408.00	5.00
3415.00	
3417.00	10.00
3417.00	
3427.00	7.00
3427.00	
3439.00	12.00
3439.00	2.00

3439.00	
3441.00	1.00
3445.00	11.00
3456.00	8.00
3462.00	
3466.00	1.00
3467.00	
3467.00	
3471.00	10.00
3473.00	2.00
3473.00	7.00
3473.00	4.00
3476.00	10.00
3476.00	2.00
3477.00	11.00
3477.00	
3485.00	11.00
3498.00	7.00
3500.00	
3501.00	11.00
3509.00	
3512.00	1.00
3512.00	8.00
3518.00	9.00
3521.00	7.00
3524.00	10.00
3524.00	
3528.00	7.00
3529.00	3.00
3529.00	12.00
3530.00	3.00
3534.00	2.00
3536.00	
3540.00	12.00
3542.00	11.00
3542.00	1.00
3546.00	1.00
3547.00	1.00
3547.00	
3551.00	1.00
3555.00	8.00
3566.00	3.00
3567.00	
3576.00	4.00
3578.00	10.00

3579.00	2.00
3579.00	8.00
3580.00	
3586.00	12.00
3596.00	7.00
3601.00	12.00
3603.00	8.00
3605.00	9.00
3606.00	
3607.00	
3609.00	2.00
3619.00	
3621.00	10.00
3623.00	2.00
3625.00	6.00
3627.00	8.00
3636.00	7.00
3636.00	
3637.00	4.00
3637.00	
3640.00	2.00
3642.00	
3644.00	6.00
3645.00	2.00
3649.00	10.00
3653.00	5.00
3653.00	2.00
3653.00	
3653.00	
3654.00	8.00
3654.00	2.00
3657.00	12.00
3663.00	4.00
3667.00	4.00
3668.00	12.00
3683.00	5.00
3683.00	2.00
3688.00	1.00
3695.00	
3698.00	11.00
3705.00	9.00
3710.00	6.00
3712.00	3.00
3713.00	
3716.00	5.00

3717.00	8.00
3718.00	
3719.00	
3722.00	
3727.00	
3729.00	6.00
3730.00	10.00
3736.00	
3745.00	10.00
3747.00	5.00
3749.00	
3753.00	9.00
3753.00	10.00
3759.00	6.00
3764.00	10.00
3764.00	11.00
3770.00	
3773.00	5.00
3773.00	
3774.00	12.00
3786.00	
3788.00	
3789.00	12.00
3791.00	5.00
3794.00	
3801.00	10.00
3802.00	1.00
3804.00	6.00
3809.00	
3812.00	
3816.00	12.00
3817.00	6.00
3821.00	11.00
3824.00	1.00
3825.00	4.00
3831.00	3.00
3831.00	11.00
3833.00	5.00
3834.00	
3835.00	6.00
3839.00	9.00
3839.00	
3845.00	7.00
3845.00	6.00
3848.00	

3851.00	1.00
3851.00	11.00
3852.00	4.00
3852.00	7.00
3852.00	5.00
3853.00	
3854.00	9.00
3854.00	7.00
3854.00	
3863.00	
3864.00	4.00
3864.00	7.00
3865.00	11.00
3867.00	8.00
3870.00	
3887.00	
3892.00	
3892.00	
3893.00	1.00
3893.00	
3893.00	
3911.00	9.00
3928.00	12.00
3928.00	6.00
3928.00	1.00
3928.00	1.00
3928.00	
3934.00	12.00
3938.00	6.00
3946.00	1.00
3953.00	
3957.00	
3959.00	4.00
3962.00	10.00
3965.00	3.00
3969.00	1.00
3970.00	6.00
3983.00	3.00
3986.00	
3996.00	
4005.00	4.00
4010.00	
4013.00	
4014.00	
4018.00	1.00

4026.00	1.00
4028.00	4.00
4028.00	2.00
4031.00	
4038.00	6.00
4039.00	10.00
4048.00	
4050.00	
4056.00	5.00
4069.00	6.00
4077.00	9.00
4092.00	
4093.00	10.00
4102.00	7.00
4102.00	6.00
4105.00	3.00
4112.00	8.00
4113.00	6.00
4113.00	
4116.00	
4119.00	9.00
4124.00	8.00
4128.00	6.00
4130.00	10.00
4136.00	1.00
4140.00	8.00
4141.00	
4155.00	4.00
4157.00	
4158.00	10.00
4162.00	5.00
4167.00	3.00
4169.00	7.00
4171.00	11.00
4183.00	
4201.00	1.00
4209.00	
4221.00	
4222.00	1.00
4222.00	
4227.00	1.00
4230.00	
4231.00	8.00
4232.00	7.00
4236.00	8.00

4241.00	1.00
4249.00	1.00
4251.00	11.00
4265.00	2.00
4268.00	3.00
4269.00	
4279.00	2.00
4284.00	
4290.00	2.00
4290.00	
4291.00	3.00
4293.00	1.00
4301.00	5.00
4311.00	12.00
4312.00	3.00
4318.00	8.00
4323.00	2.00
4325.00	10.00
4329.00	
4330.00	6.00
4334.00	5.00
4339.00	6.00
4344.00	6.00
4347.00	1.00
4347.00	3.00
4348.00	
4349.00	2.00
4352.00	3.00
4360.00	
4360.00	
4363.00	
4371.00	2.00
4374.00	
4376.00	6.00
4378.00	5.00
4378.00	11.00
4385.00	11.00
4388.00	4.00
4389.00	
4396.00	6.00
4408.00	1.00
4414.00	
4418.00	5.00
4419.00	
4421.00	1.00

4430.00	
4434.00	7.00
4436.00	11.00
4441.00	
4445.00	1.00
4472.00	7.00
4475.00	1.00
4488.00	5.00
4499.00	2.00
4499.00	
4501.00	8.00
4503.00	11.00
4504.00	12.00
4506.00	
4510.00	
4510.00	
4515.00	
4516.00	8.00
4526.00	1.00
4527.00	
4538.00	11.00
4545.00	
4556.00	6.00
4557.00	4.00
4560.00	3.00
4563.00	8.00
4601.00	
4605.00	2.00
4611.00	
4627.00	12.00
4638.00	1.00
4639.00	1.00
4654.00	5.00
4669.00	6.00
4703.00	
4704.00	2.00
4719.00	
4728.00	3.00
4748.00	4.00
4779.00	2.00
4808.00	
4836.00	3.00
4849.00	1.00
4854.00	
4863.00	

4873.00	
4923.00	12.00
4923.00	
4961.00	
4962.00	5.00
4979.00	11.00
5031.00	4.00
5052.00	6.00
5067.00	4.00
5078.00	12.00
5082.00	1.00
5103.00	
5109.00	4.00
5146.00	12.00
5154.00	11.00
5172.00	9.00
5241.00	1.00
5251.00	
5260.00	9.00
5272.00	2.00
5474.00	3.00
5481.00	7.00
5494.00	
5581.00	4.00
5589.00	1.00
5634.00	8.00
5647.00	6.00
5673.00	1.00
5842.00	
2748.00	
2788.00	
2922.00	5.00
	3.00
	2.00
	1.00
3915.00	9.00
	10.00
3987.00	
